# Impacts of mitochondrial dysfunction on axonal microtubule bundles as a potential mechanism of neurodegeneration

**DOI:** 10.3389/fnins.2025.1631752

**Published:** 2025-08-19

**Authors:** Scott Murray-Cors, Milli Owens, Yu-Ting Liew, Maureece Day, William Cairns, Andreas Prokop

**Affiliations:** School of Biology, Manchester Academic Health Science Centre, Faculty of Biology, Medicine and Health, The University of Manchester, Manchester, United Kingdom

**Keywords:** *Drosophila*, microtubules, reactive oxygen species, mitochondria, neurodegeneration

## Abstract

Mitochondrial dysfunction is an important cause for neurodegeneration, often associated with dyshomeostasis of reactive oxygen species, i.e., oxidative stress. However, apart from ATP production, mitochondria have many other functions the aberration of which may impact neurons in very different ways. Oxidative stress can cause the deterioration of axonal microtubule bundles, thus critically affecting the highways for life-sustaining transport and providing a potential path to neurodegeneration. We recently found that aberrant transport of mitochondria can have this effect by causing oxidative stress. We therefore asked which aberrations of mitochondrial physiology might impact microtubules, which of these might explain the observed consequences of aberrant mitochondrial transport, and whether mitochondria-induced microtubule phenotypes are always mediated by oxidative stress. Using one consistent *Drosophila* primary neuron system, we studied functional loss of 13 different mitochondrial factors known to be detrimental to neurons *in vivo*. Losses of five factors caused MT damage, namely pyruvate dehydrogenase A, succinate dehydrogenase A, adenine nucleotide translocase, frataxin and superoxide dismutase 2. All involved oxidative stress, hence supported the path from mitochondria via oxidative stress to microtubule deterioration; of these, we discuss superoxide dismutase 2 as potential candidate explaining effects of mitochondrial transport aberration. Six of the remaining factors not causing microtubule damage were important mitochondrial morphogenesis regulators, suggesting efficient protection mechanisms preventing oxidative stress upon mitochondrial contortion.

## Significance statement

We used one consistent *Drosophila* primary neuron system to study the deficiencies of 13 mitochondrial factors known to be detrimental to neurons *in vivo*. Five factors triggered axonal microtubule bundle decay mediated by oxidative stress, thus establishing a potential mechanism linking dysfunctional mitochondria to neurodegeneration. Six factors were important morphogenesis regulators of mitochondria and their cristae, and none of them affected microtubule bundles, suggesting efficient protection mechanisms preventing oxidative stress upon mitochondrial contortion.

## Introduction

**FIGURE 1:**
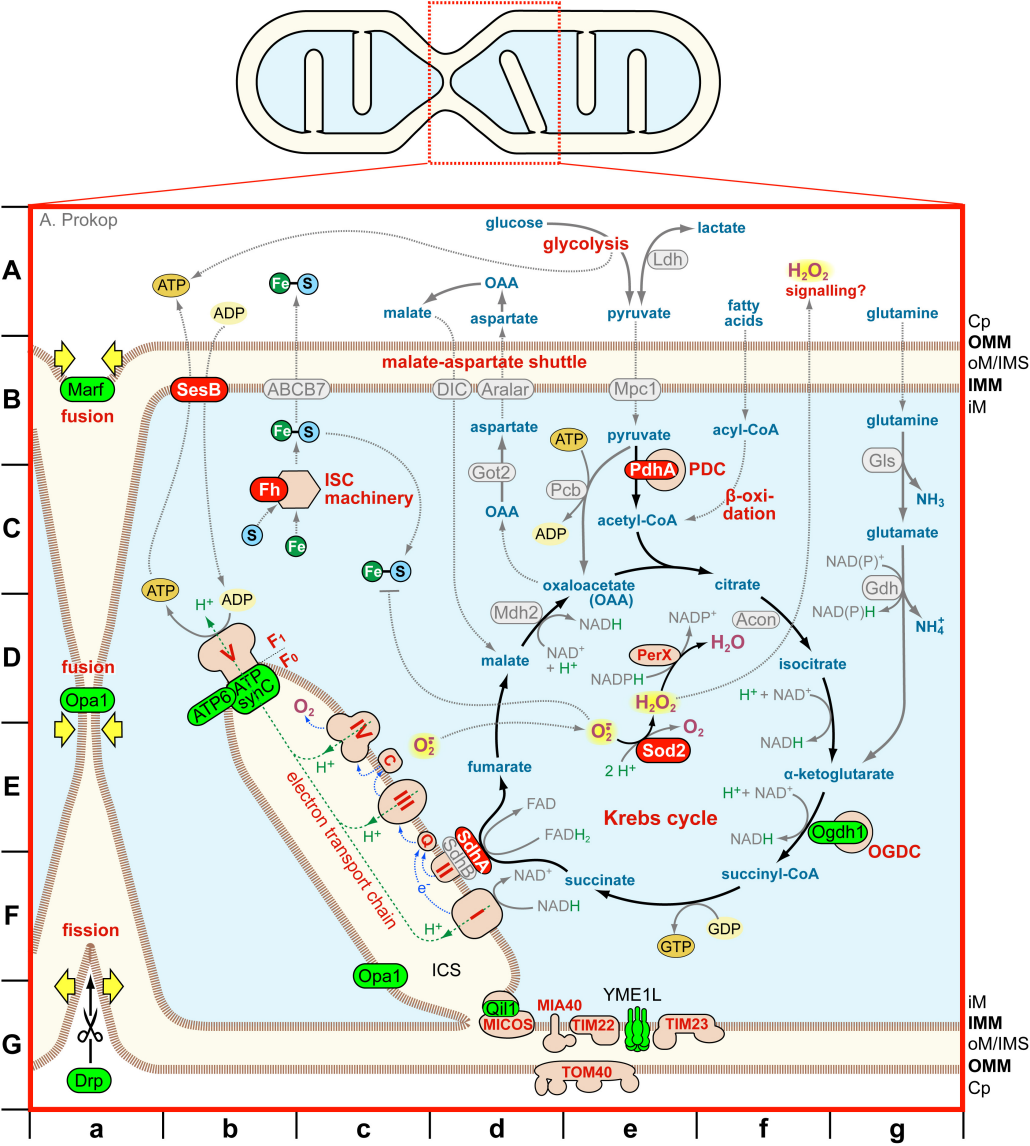
Processes and components referred to in the text that mediate mitochondrial physiology and dynamics. The sketch on top represents a mitochondrion during fusion/fission, with the red box indicating the position of the close-up shown below. Different compartments and membranes are colour-coded: cytoplasm (Cp) in white, outer mitochondrial matrix/inter-membrane space (oM/IMS) and inter-cisternal space (ICS) in light beige, inner mitochondrial matrix in light blue, inner and outer mitochondrial membranes (IMM, OMM) as stippled brown lines; for further acronyms see the dedicated abbreviation list. Protein complexes are shown in pink with red outlines; identified proteins are either in light green/dark red with black outline (proteins manipulated in this study without/with MT-curling), or in light-grey with grey outline (mentioned in the text but not investigated here). Organic and inorganic components are colour coded as follows: organic metabolites in blue, protons in green, oxygen derivatives in magenta (those being ROS highlighted in yellow), co-enzymes of redox reactions in grey, iron (Fe) in dark-green circles, sulphur (S) in blue circles, ATP/GTP in dark orange circles, ADP/GDP in light orange circles. Chemical reactions are shown as solid black or grey arrows, spatial translocations as stippled grey arrows. Letters on the left and at the bottom outside the box are grid coordinates referred to in the text (chevron followed by italics letters).

Neurodegenerative disorders are an important socioeconomic challenge to modern ageing societies (GBD 2016 Neurology Collaborators, 2019). One major cellular cause often highlighted in this context is the dysregulation of mitochondria (Wang et al., 2021), especially the excessive generation or inappropriate release of harmful reactive oxygen species (ROS) causing oxidative stress as a side product of the electron transfer chain (ETC) which drives oxidative phosphorylation (Figure 1; OXPHOS; Zorov et al., 2014).

However, mitochondria are far more than the cell’s powerhouse. For example, astrocytes, activated immune cells, tumour cells or cells during migration often circumvent the resource-efficient but slow process of oxidative phosphorylation; they instead switch to aerobic glycolysis as resource-hungry but fast and locally available means of ATP generation, which also churns out pyruvate molecules as metabolic building blocks or turning them into lactate as secondary energy source (Bhattacharya et al., 2020; Magistretti and Allaman, 2018). It was even reported that mitochondria in neuronal axons often act as ATP sink rather than source (Hirabayashi et al., 2024). Ever more roles of mitochondria are coming to light (Pfanner et al., 2019). For example, they are essential to produce iron-sulphur clusters as obligatory components of many enzymes in mitochondria, the cytoplasm or nucleus (Marelja et al., 2018; Shi et al., 2021). Mitochondria are discussed as calcium-buffering stores at synapses (Walters and Usachev, 2023), play key roles in programmed cell death involving the release of signals such as cytochrome c or mitochondrial DNA (Bonora et al., 2022; Glover et al., 2024; Khatun et al., 2024) and display direct contacts with other organelles or with each other as means of cross-regulation (Picard et al., 2015; Voeltz et al., 2024). Furthermore, mitochondria appear to respond to oxidative and metabolic states or requirements of cells by adapting their activities, changing shape, sending signals to the cytoplasm, positioning themselves into subcellular locations as extreme as the tips of filopodia or even transiting between cells; in this way they seem to help maintain cellular homeostasis or change the activity states of cells (Borcherding and Brestoff, 2023; D’Angelo et al., 2023; Marlar-Pavey et al., 2025; Palma et al., 2020; Sturm et al., 2024).

**FIGURE 2:**
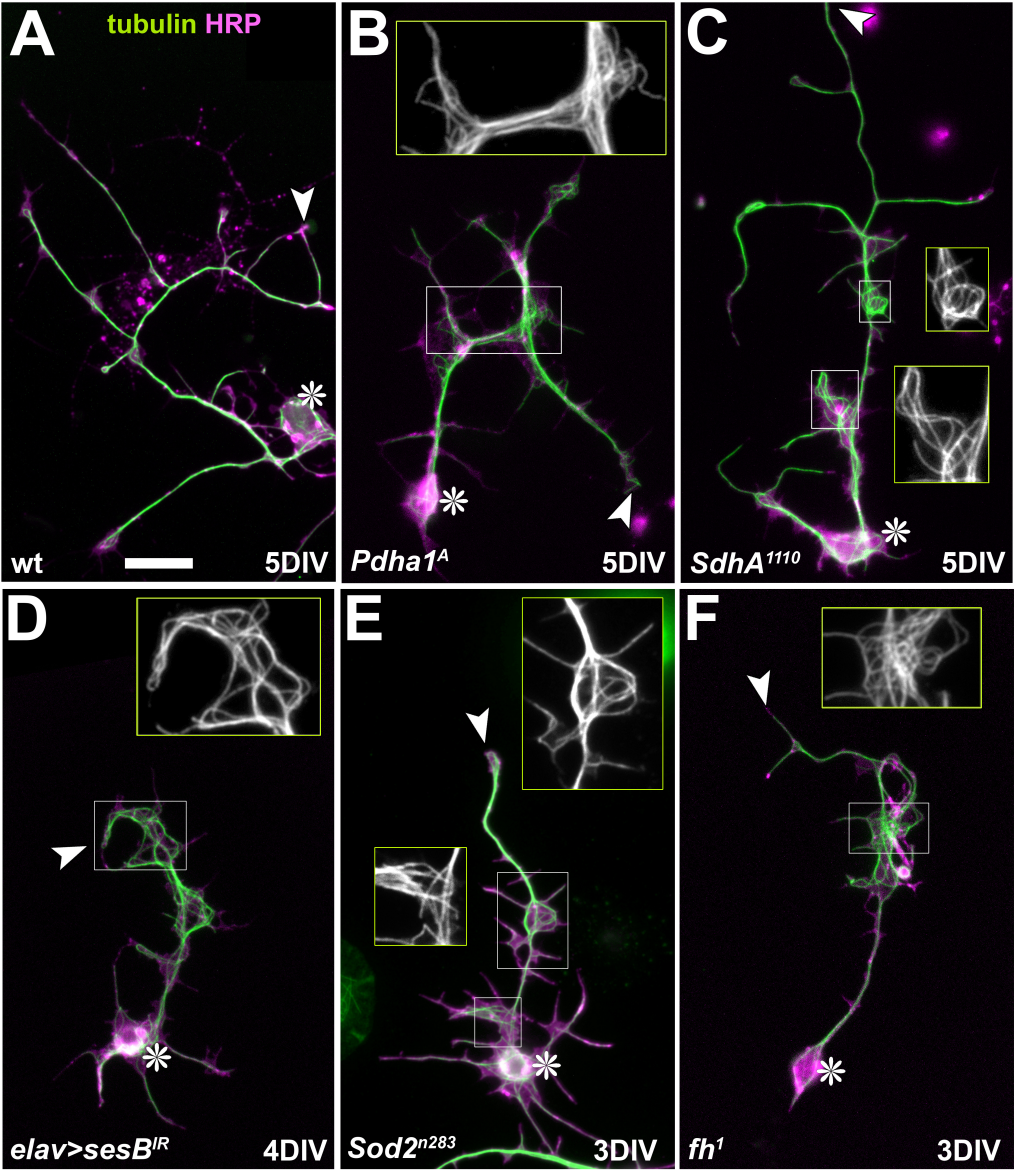
Representative images of MT-curling observed in mutant neurons. **(A–F)** *Drosophila* primary neurons of different genotypes (indicated bottom left; see text) cultured for 3, 4 or 5 days *in vitro* (DIV) stained for tubulin (green) and the neuronal surface marker HRP (magenta). Cell bodies are indicated by asterisks, axon tips by arrow heads, and emboxed areas are shown as two-fold magnified insets (tubulin channel only). Scale bar in A represents 20 μm in all images.

It is conceivable that harmful ROS production can be the outcome when aspects of this complex mitochondrial physiology become derailed (see Discussion). To add to this spectrum of possibilities, we recently observed in *Drosophila* primary neurons that even axonal transport deficits of mitochondria, including their complete absence from axons, lead to axonal ROS dyshomeostasis (Liew et al., 2025). That study further showed that axonal ROS dyshomeostasis caused severe disintegration of axonal microtubule (MT) bundles which tend to display as areas of chaotic MT-curling (Figure 2; see also Shields et al., 2025). ROS may therefore provide a potential link between the dysfunction or mislocalization of mitochondria and MT bundles. This may provide a potential mechanism explaining mitochondria-induced neurodegeneration, because MT bundles provide the essential lifeline of axons: they run uninterrupted from the neuronal cell bodies to the axonal tips serving as the highways for cargo transport required to sustain axonal cell biology; interrupting these bundles turns them into an axon’s Achilles heel on path to degeneration (Okenve-Ramos et al., 2024; Prokop, 2021; Smith et al., 2023).

Based on these observations, we therefore asked (1) whether different forms of mitochondrial dysfunction can lead to MT-curling, (2) whether this is always mediated by harmful ROS, and (3) how the absence of mitochondria from axons might lead to MT-curling. To address these questions, we carried out a pilot study systematically assessing the functional loss of 13 well-conserved mitochondrial proteins in one consistent *Drosophila* primary neuron system. Importantly, all results were compared to reports in the wider literature that will be discussed in detail.

## Results

### Experimental strategy

To address our question, we selected 13 candidate genes primarily based on their reported links to neurodegeneration, published information about their debilitating effects on *Drosophila* neurons *in vivo*, and the availability of established genetic tools for their manipulation (detailed rationales provided at start of each section; Supplementary Table 1; Brischigliaro et al., 2023; Marelja et al., 2018). To validate results, we aimed to deplete the function of each gene using at least two independent approaches, which included different loss-of-function mutant alleles, combinations of mutant alleles with deficiencies uncovering these genes, or the knock-down of genes using the targeted expression of interference RNA constructs with the pan-neuronal driver line *elav-Gal4* (see Methods). To achieve a level playing field for our analyses, all studies were carried out in one consistent *Drosophila* primary neuron system by harvesting neurons from either mutant embryos or embryos displaying gene knock-down. We used MT-curling as one consistent readout, for the underlying regulation of which we have longstanding experience (Hahn et al., 2021; Liew et al., 2025; Qu et al., 2017). Since most loss-of-function mutations used in this study are homozygous lethal, mothers have to be heterozygous and their eggs may contain deposited mRNA or protein from the healthy gene copy (referred to as maternal component) which can mask mutant phenotypes for a while. Based on our experience, a culture period of 5 days *in vitro* (DIV) is a reliable length to achieve full phenotypic penetrance (Liew et al., 2025) and was therefore chosen as standard for most analyses. However, since phenotypes can often display as early as 6 hrs *in vitro*; Gonçalves-Pimentel et al., 2011; Qu et al., 2022), some follow-up experiments were done at shorter culture periods once phenotypes were established.

### Loss of *PdhA* causes ROS-dependent MT-curling

We began our investigation with examples of factors that contribute to Krebs cycle function, starting with the pyruvate dehydrogenase complex (PDC) as the gate keeper which feeds the Krebs cycle with acetyl-CoA (Figure 1 > *A-C/e*; letters behind the chevron indicate the grid coordinates provided in the figure). PDC is composed of multiples of three enzymes (E1-3) converting pyruvate and CoA-SH into acetyl-CoA and CO_2_ whilst generating NADH; PDHA (pyruvate dehydrogenase E1 subunit α1) is an obligatory subunit of the E1 enzyme which catalyses the first reaction step (Magistretti and Allaman, 2018; Naifeh et al., 2024; Patel et al., 2014).

In humans, PDHA1 mutations constitute ∼80% of cases of Leigh disease displaying with severe early-onset neurodegenerative symptoms, PDC enzyme activity reduced to 30%, and lactic acidosis with ∼4-fold increase in pyruvate and lactate plasma levels (Foucher and Tubben, 2024; Gopal et al., 2023). Rare patients with PDHB mutations show strong clinical overlap with PDHA mutant cases (Patel et al., 2012). In *Drosophila*, *in vivo* studies showed that Pdha1-deficient photoreceptor cells degenerate when challenged with light-induced activation (Jaiswal et al., 2015). Also, strong knock-down of Pdhb (the β-subunit of E1) displayed clear neurodegenerative phenotypes (Dung et al., 2018).

**FIGURE 3:**
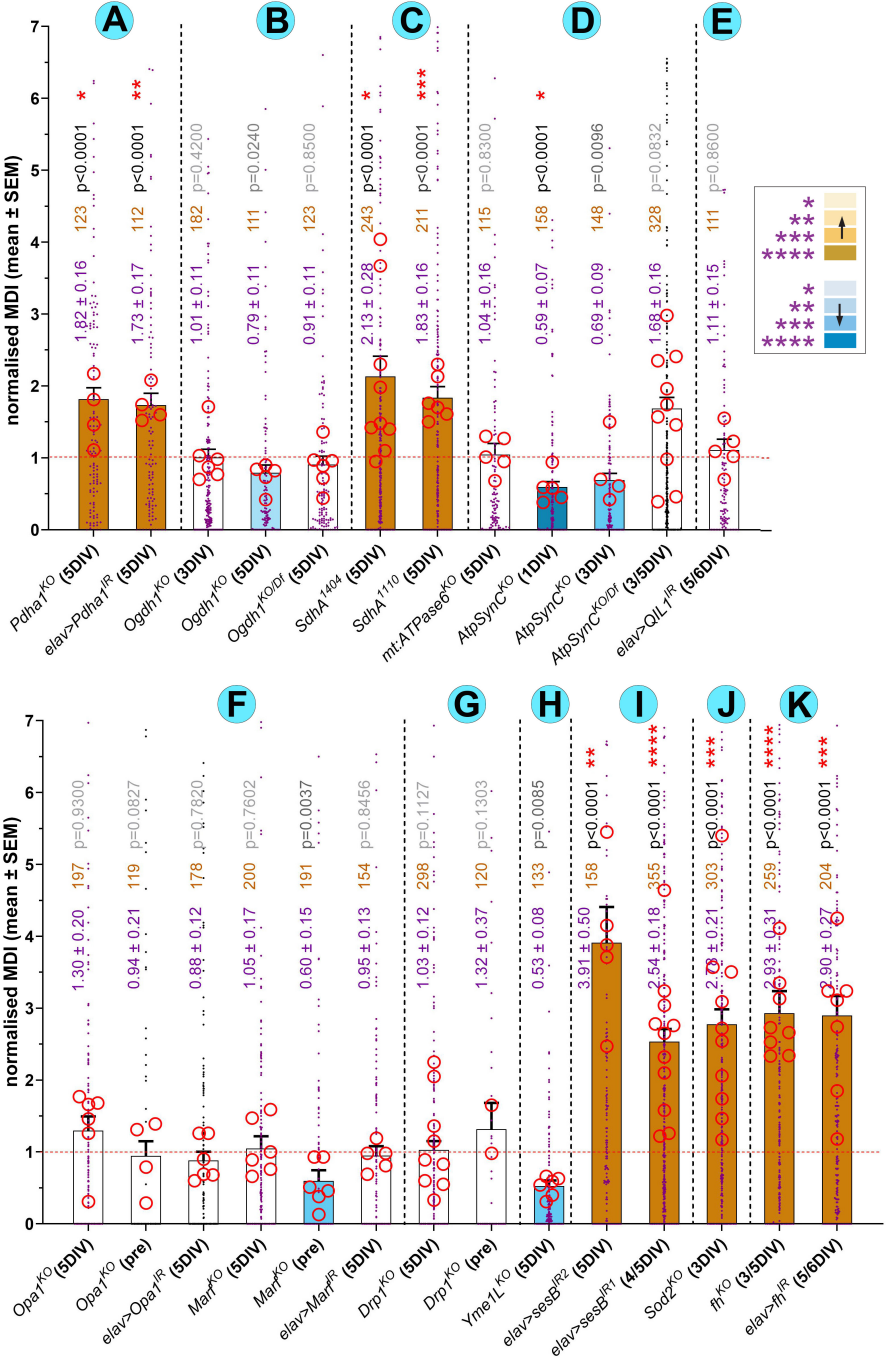
Quantification of MT-curling observed upon functional deficiency of factors involved in mitochondrial physiology. (A–K) Bars indicating the degree of MT-curling where genotypes are grouped and separated by stippled vertical lines as they are presented in dedicated sections in the main text; only in one case there is an additional subdivision separating fusion (F) from fission (G) factors. Bars indicate the degree of MT-curling indicated in blue font as the mean ± SEM MDI (MT disorganization index: the size of axonal areas displaying MT-curling relative to axon length); data are normalized to internal wild-type controls of each experiment (horizontal red dashed line); single data points are shown as blue dots and the means of repeats (independent coverslips from usually two to three independent replicates) are shown as red circles with their statistical significance (established using *t-tests*) indicated as red asterisks (*P ≤ 0.05; **P ≤ 0.01; ***P ≤ 0.001; ****P ≤ 0.0001); numbers of assessed neurons are shown in orange, their *p* values relative to controls (established by Mann-Whitney tests) in black or grey; the grey-to-black intensity of statistical values and the brown/blue bar colour (see inset) reflect the respective degrees of significance relative to wild-type.

To study loss of PDC function in primary neurons, we generated *Drosophila* primary neurons (see Methods) from embryos homozygous for the previously reported loss-of-function mutant allele *Pdha1^A^* (Liu et al., 2017; Yamamoto et al., 2014) or displaying nervous system-specific knock-down of Pdha1 (Huang et al., 2022). In both cases, primary neurons showed a robust increase in MT-curling when analysed at 5 DIV (Figures 2B, 3A), clearly confirming our hypothesis that axonal MT bundles can be affected by mitochondrial dysregulation.

**FIGURE 4:**
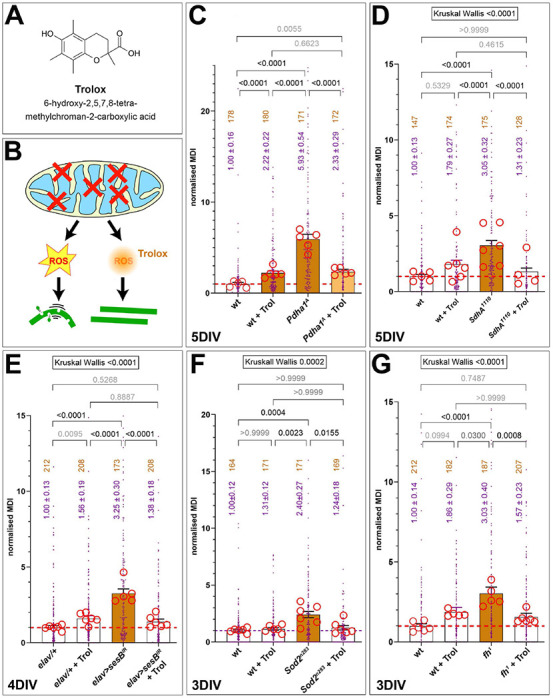
The phenotypes of all MT-curl-inducing conditions are rescued by Trolox. **(A)** The chemical structure of Trolox. **(B)** The design of Trolox experiments: mitochondrial phenotypes (red X) cause ROS (yellow star) which, in turn, causes curling and potential damage of MTs (left); upon application of Trolox (right), ROS is quenched and MT-curling abolished although the original mitochondrial phenotype is still present. **(C–G)** Each graph represents one set of experiments composed of wild-type without Trolox (wt), wt with Trolox (+ Trol) and the respective loss-of-function mutant condition (as indicated in **C–G**) without/with Trolox. Graphs are organized as explained in the legend of Figure 3. Kruskall-Wallis test results are indicated on top, Dunn’s multiple comparison results are indicated above bars; the intensity of brown fill-colour of bars reflects the degree of statistical significance relative to wild-type control.

To test whether the phenotype of *Pdha1^A^* mutant primary neurons was ROS-dependent, we supplied the culture medium for the entire culture period with 100 μM Trolox (6-hydroxy-2,5,7,8-tetramethylchroman-2-carboxylic acid). Trolox is a vitamin E analogue that acts as a ROS-scavenger; it inhibits fatty acid peroxidation and quenches singlet oxygen and superoxide and was reported to have beneficial antioxidant effects in neuronal models of neurodegeneration (Chow et al., 1994; Janc and Müller, 2014). In Pdha1-deficient neurons, Trolox clearly suppressed the MT-curling phenotype (Figure 4C) suggesting that harmful ROS dyshomeostasis mediates the phenotype. PdhA loss provides therefore an example where mitochondrial dysfunction causes ROS-mediated MT-curling.

### Loss of Ogdh1 seems not to cause excessive ROS

Another Krebs cycle component we selected is the oxoglutarate dehydrogenase complex (OGDC) which converts α-ketoglutarate (aka oxoglutarate) to succinyl-CoA (Figure 1 > *EF/fg*). Ogdh1/OGDH encodes the E1 component of the complex, which catalyses the initial step of the reaction (Nemeria et al., 2021).

In humans, OGDH deficiency causes movement disorders and hyperlactatemia (Yap et al., 2021b). In *Drosophila*, the *Ogdh1*^*MI*06026–TG4.1^ loss-of-function allele is embryonic lethal, and likewise when loss of Ogdh1 is restricted to the nervous system; its knock-down in photoreceptors causes progressive loss of synaptic transmission (Yap et al., 2021a; Yap et al., 2021b; Yoon et al., 2017; Supplementary Table 1).

We therefore assessed primary neurons carrying the lethal *Ogdh1*^*MI*06026–TG4.1^ mutant allele in homozygosis or over deficiency. These neurons displayed no obvious increase in MT-curling at 5 DIV, in one set of experiments even a potential bias to reduce MT-curling (Figure 3B).

Therefore, although loss of Ogdh1 is a lethal conditions, no MT phenotypes became apparent in neurons during the 5 day culture period.

### SdhA deficiency causes MT-curling suppressed by Trolox

As a further Krebs cycle component we selected succinate dehydrogenase (SDH) which is an enzymatic complex formed by at least four distinct constituent subunits A to D. It participates in the Krebs cycle catalyzing the step from succinate to fumarate (Figure 1 > *EF/cd*). However, SDH is a special case in that it forms not only part of the Krebs cycle but also constitutes complex II of the ETC passing on electrons from its enzymatic reaction via ubiquinone to complex III (Figure 1 > *EF/cd*; Al-Rasheed and Tarjan, 2018; Rutter et al., 2010).

In humans, SDHA mutations cause neurodegeneration, muscle weakness and tumor formation (Al-Rasheed and Tarjan, 2018; Hadrava Vanova et al., 2020; Patel et al., 2012; Rutter et al., 2010). The *Drosophila SdhA*^1110^ and *SdhA*^1404^ mutant alleles reduce SDH activity substantially causing recessive larval lethality; upon mosaic analysis, photoreceptor cells homozygous for these alleles showed gradual synapse loss (Mast et al., 2008). Furthermore, heterozygous deficiency of *SdhA* strongly enhances premature death of flies lacking the SDH assembly factor Sirup/SDHAF4 (Van Vranken et al., 2014) and *SdhA*^1110^ genetically interacts with the Parkinson gene *park* enhancing its motility deficits (O’Hanlon et al., 2022).

Our analyses of primary neurons homozygous for the above-mentioned *SdhA*^1404^ or *SdhA*^1110^ mutant alleles, revealed a robust MT-curling phenotype at 5 DIV (Figures 2C, 3C). To test whether the phenotype of *SdhA*-deficient primary neurons was ROS-dependent, we applied the ROS-scavenger Trolox (Chow et al., 1994) which resulted in robust suppression of the MT phenotype (Figure 4D). Functional loss of SdhA is therefore another condition demonstrating deteriorating impacts of mitochondrial dysfunction on axonal MT bundles mediated by oxidative stress.

### Loss of ATP synthase does not induce MT-curling

We next focused on complex V of the ETC, also known as ATP synthase. ATP synthase is a multi-subunit complex clustered at the tips of cristae. Its channel-forming transmembrane sub-complex F_0_ permits the flow of protons across the inner membrane which provides the energy that drives ATPase activity of the enzymatic matrix-facing sub-complex F_1_ (Figure 1 > *D/b*). APT synthase also plays important roles in cristae morphogenesis by mediating the extreme membrane curvature of their tips (Guo et al., 2017; Hahn et al., 2016; Zhou et al., 2015), and it is proposed to form a component of the mitochondrial permeability transition pore (mPTP; Bonora et al., 2022).

Here, we chose two key components of the F_0_ complex crucial for ATP synthase function: ATP5MC1-3 (ATPsynC in flies) constitutes the multimeric proton-conducting c-ring, whereas mitochondrially encoded MT-ATP6 (mt:ATPase6 in fly) closely associates with the c-ring and is required for its proton-conducting ability (Guo et al., 2017; Hahn et al., 2016; Zhou et al., 2015).

In humans, the dystonia-linked ATP5MC3^*N*106*K*^ mutation causes a reduction in ATP production and oxygen consumption (Neilson et al., 2022), and MT-ATP6 mutations are linked to severe neurological conditions (Dautant et al., 2018; Galber et al., 2021). In *Drosophila*, loss of ATPsynC (*ATPsynC*^*KG*01914^) causes a reduction in mitochondrial cristae and animals die as larvae after a prolonged developmental block (Lovero et al., 2018). Furthermore, ubiquitous expression of ATPsynC^*N*102*K*^ (mimicking human ATP5MC3^*N*106*K*^) caused strongly reduced ATP synthase activity coupled to lethality (Neilson et al., 2022). Individuals carrying the strong *mt:ATPase6^1^* loss-of-function allele (a G116E point mutation) were reported to be almost 100% mutant (homoplamic) leading to total loss of ATP synthase activity and aberrant mitochondria with severely rounded cristae; surprisingly, flies can eclose but display a vastly reduced adult lifespan (Celotto et al., 2011; Celotto et al., 2006; Demine et al., 2019). We similarly observed that *mt:ATPase6^1^* mutant flies showed severely reduced mobility already at 2 weeks and a strong tendency to display seizures.

Our analyses of neurons homozygous for the above-mentioned lethal null mutant allele *ATPsynC*^*KG*01914^ (Lovero et al., 2018) revealed no obvious increase in MT-curling compared to wild-type controls at 3 or 5 DIV; in some experiments it even caused a reduction in curling (Figure 3D). Likewise, *mt: ATP6^1^* mutant neurons failed to reveal any increases in MT-curling at 5 DIV (Figure 3D). The combined outcome for both gene deficiencies strongly suggests that the loss of ATPase function, known to affect OXPHOS and cristae formation, seems not to cause harmful ROS, at least during the culture period assessed.

### Depletion of QIL1 or fission and fusion factors does not cause MT-curling within 5 days

Surprised that the expected aberration of cristae upon loss of ATP synthase seems not to be a ROS-inducing condition, we challenged this finding further. For this, we studied other factors involved in cristae formation, namely the MICOS complex and Opa1. The MICOS complex is located at the neck of cristae (cristae junctions; Figure 1 > *G/d*) required for cristae formation, the stabilization of cristae junctions, and the assembly of various protein complexes at this site (Mukherjee et al., 2021). Upon loss of the QIL1/MICOS13 subunit, the entire MICOS complex fails to form, causing reduced ETC activity and severe cristae aberrations (Mukherjee et al., 2021).

In humans, QIL1 mutations are linked to diseases with neurodegenerative traits (for example COXPD37; omim.org #618329). In *Drosophila*, knock-down of QIL1 in the nervous system and muscles reduced expression to under 25% accompanied by severe aberration of mitochondria, an increase in mitophagy, but no obvious induction of cell death (Guarani et al., 2015; Wang et al., 2020). We used the same knock-down construct in primary *Drosophila* neurons, but no obvious MT-curling phenotype was detectable at 5 DIV (Figure 3E).

OPA1 is also positioned at the base of cristae and known to cause their disruption when dysfunctional (Figure 1 > *G/c*; Quintana-Cabrera and Scorrano, 2023). However, OPA1 has an additional function in that it also regulates mitochondrial fusion (Figure 1 > *D/a*). We therefore extended our study by including a second pro-fusion factor MFN (Figure 1 > *B/a*) and the pro-fission factor DNM1L (Figure 1 > *G/a*; Quintana-Cabrera and Scorrano, 2023).

**FIGURE 5:**
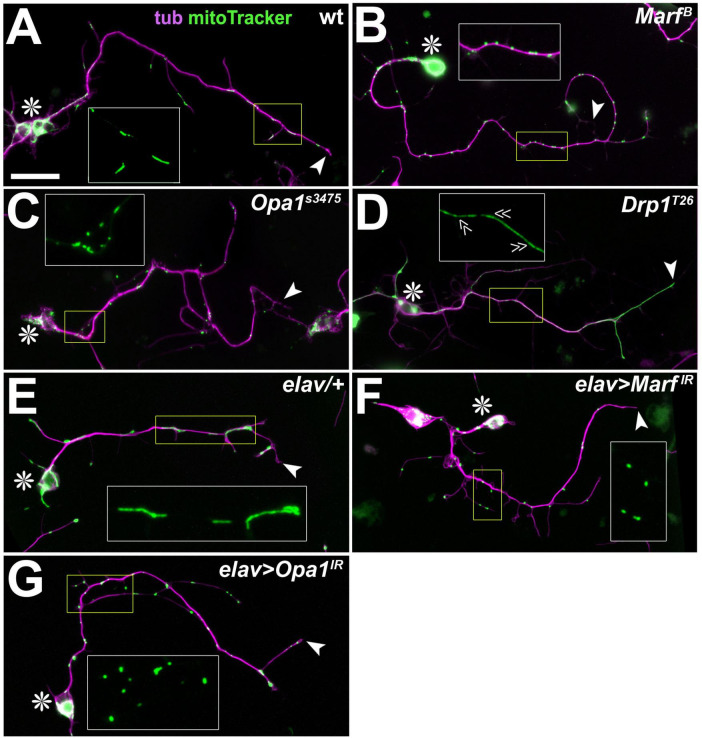
Genetic manipulations impairing mitochondrial fission/fusion processes. Neurons at 5 days *in vitro* (DIV) and stained with anti-tubulin (tub, magenta) and mitoTracker (green); they are wild-type **(wt; A)** or deficient for mitochondrial pre-fusion factors Marf **(B, F)** and Opa1 **(C, G)** and the mitochondrial pre-fission factor Drp1 **(D)**; asterisks indicate cell bodies, arrow heads axon tips, yellow emboxed areas are shown as 2-fold enlarged insets (green channel only); the scale bar in A represents 30 μm in A–D and 15 μm in E–G; note that mitochondria tend to appear as dashed lines in controls **(A)**, as sparse dots upon loss of fusion **(B, C)** and as a continuous structure excluded from many side branches upon loss of fission (“double-chevrons” in D). Quantification of MT-curling phenotypes under these mutant conditions are given in Figure 3.

In humans, all three pro-fission and -fusion factors have been linked to neurodegeneration (Supplementary Table 1; Chen W. et al., 2023), and in *Drosophila* their losses cause lethality. However, studies of the human, mammalian or fly genes draw an inconclusive picture as to whether the pathologies involve harmful ROS production (Supplementary Table 1; see section “Discussion”). We therefore assessed losses of Opa1 (lethal *Opa1*^s3475^ null allele and *elav* > *Opa1*^*IR*^), the MFN orthologue Marf (lethal *Marf^B^* null allele and *elav* > *Marf^IR^*) and the DNM1L orthologue Drp1 (lethal *Drp1*^*T*26^ null allele) in *Drosophila* primary neurons. We observed no MT-curling at 5 DIV, even when using pre-culture to exclude potential maternal rescue (see section “Methods”; Figures 3F, G, 5B–D, F, G). Functional loss of the three factors in the assessed neurons was clearly indicated by fragmented mitochondria when depleting Opa1 or Marf (Figures 5A–C, E–G) and long stretches of continuous mitoTracker-labelled structures along primary axons of Drp-deficient neurons (Figure 5D) as similarly reported for mammalian neurons (Berthet et al., 2014; Uo et al., 2009; Yu et al., 2011).

Taken together, all six factors involved in mitochondrial morphogenesis or cristae formation assessed in our standardized neuron system do not induce MT-curling during the 5 day culture period, suggesting that contorted mitochondria are well protected from harmful ROS dyshomeostasis (see Discussion).

### Loss of YME1L causes a robust reduction in MT-curling

The remaining factors addressed by our study cover a diverse range of further mitochondrial functions. For example, YME1L1 forms a homo-hexameric i-AAA protease which localizes in the vicinity of the translocase complexes of the outer and inner mitochondrial membranes (TOMM and TIMM; Figure 1 > *G/e*); YME1L1 plays important roles in proteolytic protein turn-over and the regulation of mitochondrial import and maturation (Kan et al., 2024).

Human patients homozygous for a hypomorphic YME1L1 mutation display onset of degeneration at childhood accompanied by an increase in lactate/pyruvate ratio indicative of glycolysis upregulation (Hartmann et al., 2016). In mice, ubiquitous knock-out is lethal due to heart dysfunction (Wai et al., 2015), and nervous system-specific loss causes late-onset neurodegeneration and aberration of mitochondrial morphology and transport (Sprenger et al., 2019). Similarly, also *YME1L^del^* flies display neurodegeneration of photoreceptors, reduced locomotion and premature death at adult stage, correlating with severe mitochondrial pathology including reduced and malformed cristae and electron-dense inclusions likely due to unfolded protein stress (Qi et al., 2016).

Surprisingly, we found that primary neurons homozygous for the protein null allele *YME1L^del^* displayed a consistent drastic reduction in MT-curling to about half of the values observed in parallel wild-type controls at 5 DIV (Figure 3H). These findings might suggest a reduction in ROS production below base levels present in control neurons. Baseline levels of ROS in *Drosophila* primary neurons might be slightly elevated because they are grown in an environment with higher oxygen levels than experienced *in vivo.* The primary neurons might therefore have some bias for default MT-curling which could be reduced when some natural sources of ROS production are removed. This same effect is not observed upon Trolox application (Figure 4), which may be because Trolox represents a rather crude and non-discriminatory intervention with potential negative side effects.

### Loss of non-redundant ANT in neurons causes MT-curling

Adenine nucleotide translocase (ANT) is a highly abundant mitochondrial protein in the inner mitochondrial membrane that acts as an ADP/ATP antiporter (“SesB” in Figure 1 > *B/b*); it is also considered to be a component of the low-conductance mPTP helping to avoid mitochondrial calcium stress, and to mediate proton leakage involved in mitochondrial uncoupling (Bround et al., 2020; Chen Y. et al., 2023; Demine et al., 2019).

In humans, ANTs are discussed in the context of neurodegenerative diseases including AD and PD (Chen Y. et al., 2023). However, there are 4 functionally redundant ANT genes, of which the well-studied ANT1/SLC25A4 gene is expressed in brain, heart, muscles, lung and testis (linked to cardiomyopathy; OMIM #103220) and the poorly researched ANT3/SLC25A6 gene is ubiquitously expressed; very little is known about ANT2/SLC25A5 and ANT4/SLC25A31 (Chen Y. et al., 2023). In *Drosophila*, ANTs are encoded by the *sesB* gene (highly expressed in the nervous system) and the paralogous *Ant2* gene (expressed at very low levels; Supplementary Material 1; Li et al., 2022). Therefore, loss of SesB can be considered a total loss of ANT function in the nervous system; *in vivo* studies in *Drosophila* larvae or flies revealed decreased ATP production, reduced calcium response of mitochondria, elevated peroxide levels, clear signs of age-related neurodegeneration and enhanced autophagy (Celotto et al., 2006; DeVorkin et al., 2014; Terhzaz et al., 2010; Vartiainen et al., 2014).

In agreement with these *in vivo* findings, knock-down of *sesB* with two independent constructs showed a strong increase in MT-curling in primary neurons at 4 or 5 DIV (Figures 2D, 3I). When treated with Trolox, the MT-curling was reduced to control levels, suggesting harmful ROS dyshomeostasis as the curl-inducing factor (Figure 4E). This presents another example where mitochondrial dysfunction can affect neurons through impacting their axonal MT bundles in a ROS-dependent manner.

### Sod2 deficiency causes ROS-dependent MT-curling

The mitochondrial manganese-containing superoxide dismutase SOD2 converts the highly toxic but short-lived superoxide (the main ETC-derived ROS; Andreyev et al., 2005; Murphy, 2008; Zorov et al., 2014) into the less aggressive H_2_O_2_ (Figure 1 > *DE/e*) that is longer-lived and believed to diffuse into the cytoplasm contributing to local signaling (Figure 1 > A/f; Palma et al., 2020).

Human SOD2 mutations are suggested to link to neurodegenerative diseases and conditions (Flynn and Melov, 2013; Houldsworth, 2024), although these associations are less clear than the ALS-links of SOD1 (Kim et al., 2020). SOD2 knock-out mice display increased apoptosis and urine acidity, cardiomyopathy and die after birth (Kokoszka et al., 2001; Li et al., 1995; Melov et al., 1998). If restricting SOD2 loss to the nervous system, severe neurodegeneration is observed (Melov et al., 1999; Oh et al., 2012; Qi et al., 2003). In the *Drosophila* brain, heterozygosity for *Sod2^Δ2^* and *Sod2*^*n*64^ causes precocious axon decay and MT-curling (Shields et al., 2025), and the *Sod2*^*n*283^ allele caused reduced life span (rescued by hypoxia), early onset neurodegeneration at tissue and behavioral levels, enhanced apoptosis of brain cells, and sensitivity to oxidative stress (Dias-Santagata et al., 2007; Duttaroy et al., 2003; Paul et al., 2007; Piazza et al., 2009; Vrailas-Mortimer et al., 2011; Wicks et al., 2009).

We therefore used the *Sod2*^*n*283^ mutant allele in homozygosis in primary neuron culture at 3 DIV. These experiments revealed a strong increase in MT-curling, which was suppressed by Trolox, indicating involvement of harmful ROS (Figure 2E, 3J, 4F). Very similar results were recently reported by others using the *Sod2^Δ2^* and *Sod2*^*n*64^ mutant alleles, clearly demonstrating validity of our findings (Shields et al., 2025) and identifying Sod2 deficiency as another example of mitochondrial dyshomeostasis that causes ROS-dependent MT-curling.

### Loss of frataxin causes ROS-dependent MT-curling

FRATAXIN (FXN) is an iron-binding mitochondrial protein involved in the early steps of iron-sulphur cluster formation (ISCs; Figure 1 > C/b; Monfort et al., 2022). Ion-sulphur clusters are essential functional components of many proteins in mitochondria (including many ETC components), the cytoplasm and nucleus (Vallières et al., 2024). Although iron-sulphur cluster maturation and assembly into cytosolic and nuclear proteins takes place in the cytoplasm, the early steps always depend on mitochondria, making FXN a key factor in this functional context (Fan et al., 2022; Marelja et al., 2018; Marquez et al., 2023; Shi et al., 2021).

In humans, FXN mutations link to Friedreich’s ataxia as the most common form of autosomal recessive ataxia displaying with severe neurodegenerative pathology (Delatycki et al., 2000; Williams and De Jesus, 2024). In mouse models, this pathology is reproduced and correlates with iron accumulations and oxidative stress (Al-Mahdawi et al., 2006; Simon et al., 2004).

In *Drosophila*, Frataxin (Fh) loss causes strong neurodegeneration with dying-back symptoms of peripheral axons, aberrant mitochondrial appearance, enhanced mitophagy, increased iron uptake in mitochondria of the nervous system, and higher sensitivity to iron intake (Chen et al., 2016; Edenharter et al., 2018; Llorens et al., 2007; Navarro et al., 2015; Shidara and Hollenbeck, 2010). A reduced lifespan of flies was also observed when knocking down *frataxin* specifically in neurons (Anderson et al., 2005). From all these studies in flies, there are contradicting opinions as to whether Frataxin loss induces ROS (Marelja et al., 2018).

Employing genetic tools used for the above-mentioned *in vivo* experiments (Supplementary Table 1; the lethal S136R point mutation *fh*^1^ and *elav* > *fh*^*RNAi.A*2^) in primary neurons, we found a strong increase in MT-curling at 3, 5 and 6 DIV (Figures 2F, 3K). When applying Trolox, we found a clear reduction of the phenotype down to control levels (Figures 4G), indicating harmful ROS as the mediating factor.

Taken together, 5 out of 13 factors clearly caused ROS-dependent MT-curling establishing MT bundle deterioration as a potential mechanisms leading from mitochondrial dysfunction to neurodegeneration.

### Harmful ROS triggered by loss of Fh or SesB affects Eb1 amounts at MT plus ends

**FIGURE 6:**
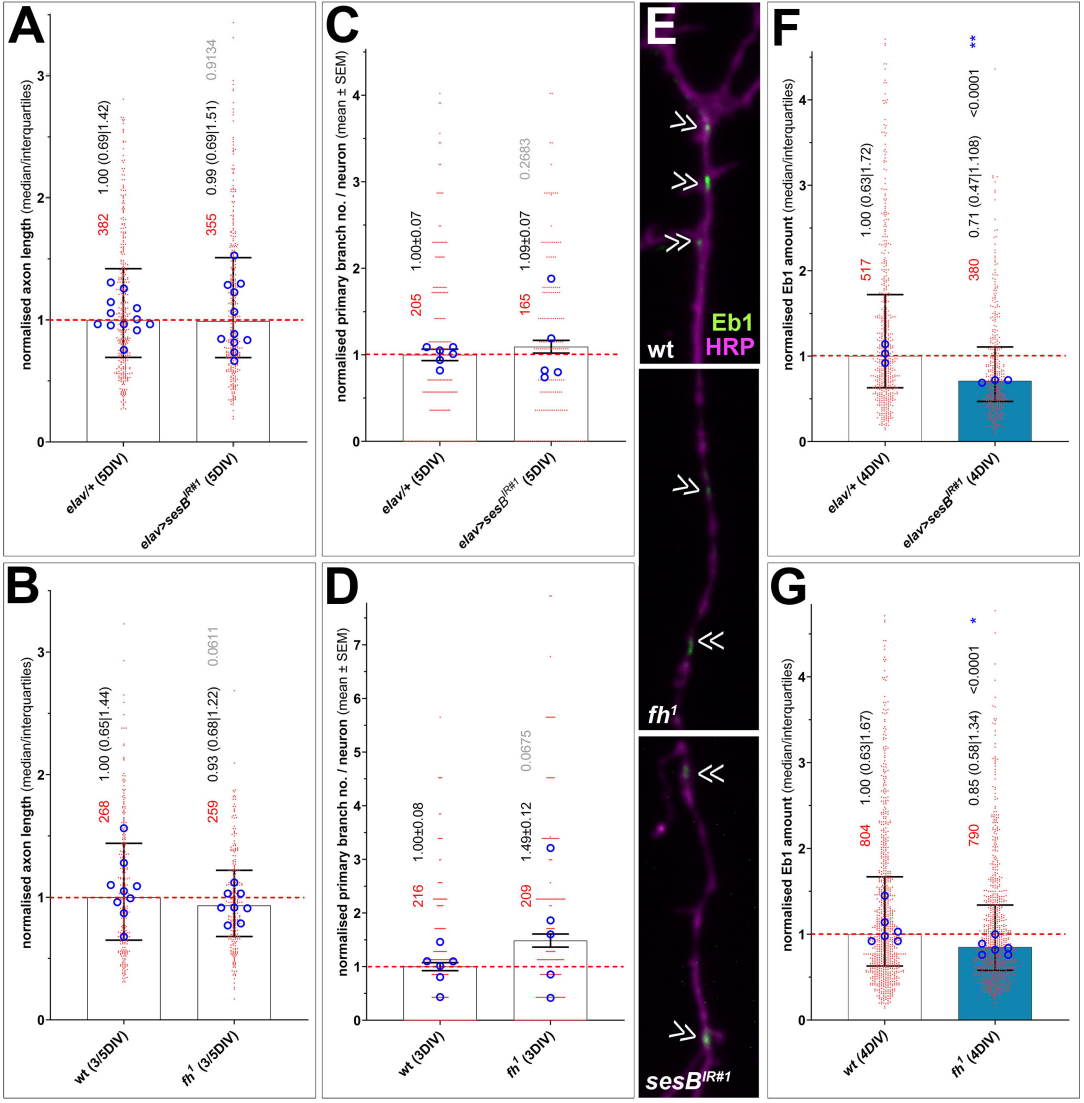
Functional losses of Fh and SesB do not affect neuronal morphology but trigger loss of Eb1 from polymerizing MT tips. Quantification of axon growth **(A, B)**, axon branching (number of primary neurites; **C, D)** and Eb1 amounts at MT plus ends **(E-G)** in neurons with loss of SesB (top) or Fh (bottom); images in E show axonal segments (stained for Eb1 in green and HRP in magenta); chevrons indicate Eb1 comets. In the graphs, bars indicate the median with quartile ranges or mean ± SEM (numerical values shown in black font); data are normalized to internal wild-type controls of each experiment (stippled line); single data points are shown as red dots (p values relative to controls established by Mann-Whitney tests are shown in black or grey) and the means of replicates (independent coverslips from usually two experimental repeats) are shown as blue circles with their statistical significance established using *t-tests* indicated as blue asterisks (*P ≤ 0.05; **P ≤ 0.01); numbers of assessed neurons are shown in red; the dark-blue bar colour reflects the degree of significance.

To test whether ROS produced upon loss of mitochondrial factors has an impact on other cell parameters, we used primary neurons mutant for *fh*^1^ or with *elav-Gal4*-driven knock-down of *sesB* and assessed their morphological parameters. Neither axon length nor branch patterns (number of primary neurites) appeared affected (Figure 6). To assess whether other MT parameters were changed upon loss of these factors, we assessed the amount of Eb1 as an indicator of MT polymerization (Hahn et al., 2021) and found a robust reduction in both cases. This reduction aligns with previous publications reporting reduced MT polymerization upon ROS increase (Conze et al., 2025; Shields et al., 2025).

## Discussion

### Only certain forms of mitochondrial aberrations impact axonal MT bundles

**FIGURE 7:**
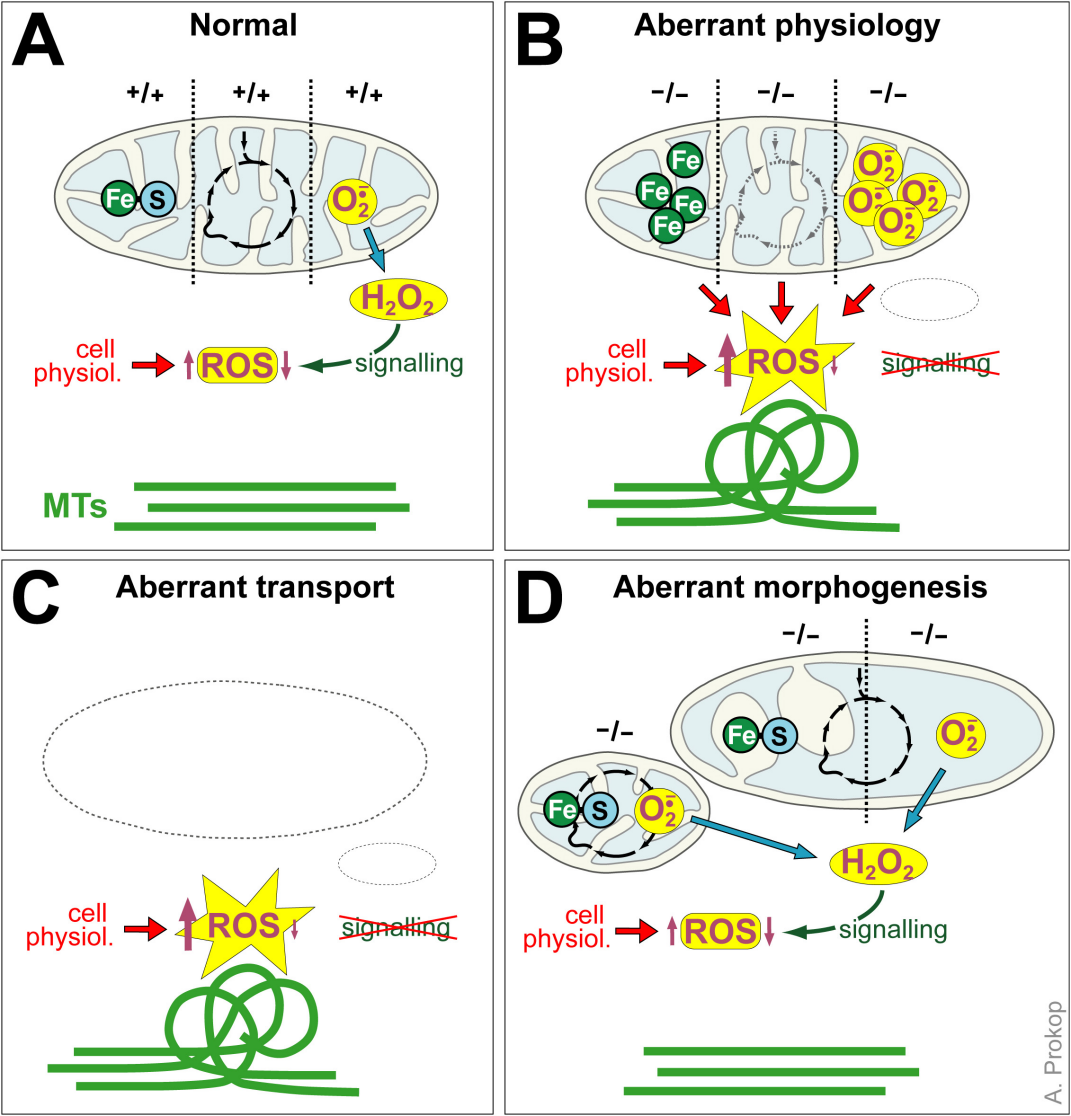
Potential interpretations of our findings. **(A)** In wild-type neurons, mitochondria generate iron-sulphur clusters (Fe-S), perform Krebs cycle metabolism (black circle of arrows) and superoxide is metabolised by Sod2 into hyperoxide which triggers signaling in the cytoplasm that helps keeping ROS in balance (up- and down-arrows) and leaves MT bundles unaffected (straight green lines). **(B)** Mutations that affect iron-sulphur production (leading to iron accumulation), that derail Krebs cycle metabolism (potentially leading to aberrant ETC function) or prevent the break-down of hyperoxide may all lead to harmful ROS in the cytoplasm surrounding mitochondria which can cause local MT-curling. **(C)** If mitochondria are absent from axons or wrongly positioned, Sod2-mediated hyperoxide signaling is absent, thus destabilizing ROS homeostasis. **(D)** Contorted mitochondria (here shown fragmented mitochondria from lack of fusion on the left, mitochondria with enlarged or missing cristae on the right) seem well protected and maintain key physiological properties that uphold ROS homeostasis in the cytoplasm.

Here we used one consistent *Drosophila* primary neuron system to apply over 30 different genetic and experimental manipulations to study 13 genes important for mitochondrial physiology, the loss of which has been reported to be detrimental to neurons *in vivo* (Supplementary Table 1). We asked (1) whether any of these assessed mitochondrial dysfunctions can affect axonal MT bundles, (2) whether such effects are mediated by harmful ROS, and (3) through which mechanisms even the absence of mitochondria might be able to cause MT-curling (see section “Introduction”). Since MT bundles provide the lifelines of axons (see section “Introduction”), potential pathological links from mitochondria to MTs might provide new mechanistic explanations for neurodegeneration. We chose the *Drosophila* primary neuron model because it has proven instrumental in the past when addressing other complex cell biological phenomena of axon biology, and was successfully used to demonstrate the fundamental principal that ROS causes MT-curling (Gonçalves-Pimentel et al., 2011; Hahn et al., 2021; Liew et al., 2025; Prokop et al., 2013; Qu et al., 2022; Shields et al., 2025; Voelzmann et al., 2024). Echoing this tradition, also our current approach delivered meaningful results:

Firstly, deficiencies of 5 out of 13 genes caused MT-curling, and all five conditions could be rescued by Trolox suggesting involvement of harmful ROS. Whilst being in line with the generally accepted view that certain mitochondrial aberrations can cause oxidative stress, they also clearly confirm that this impacts MT-bundles as a down-stream effect (Figure 7B). MT bundle deterioration provides therefore a potential mechanism that links mitochondrial dysfunction to axon degeneration. The consistency of our findings might also suggest MT-curling as a complementary, easy-to-access readout for harmful ROS generation, although further validation including direct ROS measurements in the narrow axonal cytoplasm would be required – but achieving this goal will be a highly challenging task.

Secondly, the results were surprising in that 8 out of the 13 gene deficiencies showed no obvious increases in MT-curling (Ogdh, QIL1, Opa1, Marf, Drp1, ATPsynC, mt:ATPase6, YME1L; Figure 3) despite their established lethality or severely debilitating effects *in vivo*. It was even more surprising that, except Ogdh and YME1L, all these factors are morphogenesis regulators of mitochondria and/or their cristae. Our finding suggests therefore that morphogenetic aberration is a condition that mitochondria can compensate for (at least for several days), potentially through the various stress-response mechanisms that are being reported (Figure 7D; Patergnani et al., 2022; Picca et al., 2023).

We feel that the approach taken here has delivered promising results, and the genetically amenable *Drosophila* neurons used provide the means to extend the study to an even wider scope of mitochondrial genes. Using primary neurons for this study has the advantage that we can deduce cell-autonomous effects directly caused within neuron, but it cannot cover for effects that are more indirect, for example originate in glia cells; this can only be explored in *in vivo* or co-culture studies. It will also be important to test whether our findings apply to vertebrate neurons, capitalizing on the fact that mitochondrial genes and MT regulators are highly conserved across species (Supplementary Material 2; Brischigliaro et al., 2023; Prokop et al., 2013), and the established knowledge that MT-curling occurs in mouse neurons where it can also be induced by oxidative stress (Liew et al., 2025; Smith et al., 2023).

However, as will be discussed in the next section (see also Supplementary Table 1), our mining of the literature regarding the knowledge about the 13 genes in *Drosophila in vivo* as well as their orthologues in vertebrate and human cells, often revealed inconsistent results as to whether ROS is involved in their pathological phenotypes. These contradictions are likely due to the highly diverse cellular models used which often have very different metabolic footprints, such as liver cells with extreme metabolism rates or cancer cells which tend to switch off oxidative phosphorylation. Findings are therefore difficult to extrapolate between cellular models, and their integration into conceptual understanding is limited. This is different for our results which were all obtained in one standardized neuron model where consistent findings can establish the necessary confidence to deduce concepts. It is then even more ensuring that reports in the literature can be found for all 13 genes that support our findings, as will be discussed in the following.

### Potential mechanisms that cause harmful ROS production upon loss of the 5 identified genes

We find ROS-induced MT-curling upon loss of Pdha1, although ROS seems not to feature in the current Pdha1-related *Drosophila* literature. However, ROS was reported from mammalian studies. Skin fibroblasts derived from PDHA-mutant patients showed increased mitochondrial but not cytoplasmic ROS levels (Glushakova et al., 2011), whereas increased cytoplasmic ROS levels were observed in PDHA1-deficient rat fibroblasts where Krebs cycle activity was partially maintained by glutamine-derived α-ketoglutarate (Figure 1 > A–E/g; Wang et al., 2019). In studies of PDH-deficient mouse skeletal muscle, the ETC displayed low efficiency which was compensated for by increased ETC activity - a potentially powerful constellation to cause electron leakage and generate ROS (Gopal et al., 2023). In our culture model, loss of Pdha1 might have even stronger impact on Krebs cycle activity and ETC because the loss of PdhA-derived pyruvate cannot be replenished from β-oxidation (Figure 1 > *C/f*), since fatty acids are virtually absent from the culture medium (Else, 2020; Prokop et al., 2012; Schneider, 1964). Another potential mechanism for ROS generation could be the build-up of lactate (Figure 1 > *A/e*) which causes harmful hyper-lactylation of proteins (Chapp et al., 2021; Wang et al., 2024; Yang et al., 2024). However, build-up of lactate is highly unlikely in *Drosophila* primary neurons which are grown in lactate-free culture medium (Schneider, 1964). In our view, impacts of Krebs cycle aberration on the ETC are the most likely reason for harmful ROS generation upon PDHA deficiency.

SdhA-linked pathology in *Drosophila* has been linked to ROS *in vivo*, which agrees with our findings. For example, antioxidants could reduce synapse loss in *SdhA*^1110^ and *SdhA*^1404^ mutant photoreceptors (Mast et al., 2008), and SdhA seems to be part of the Cnc/Nrf2-mediated oxidative stress response pathway (Tsakiri et al., 2019a). For vertebrates, we found reports of ROS elevation for loss of subunits B, C and D (Goffrini et al., 2009; Guzy et al., 2008; Hadrava Vanova et al., 2020; Ishii et al., 2011; Li et al., 2019; Owens et al., 2012), but there were also arguments against ROS. For example, SDHB-deficient chromaffin cells had reduced oxygen consumption (Kuèková et al., 2020), SDH was suggested to act as a ROS sensor dampening Krebs cycle and ETC activity upon elevated H_2_O_2_ levels (Nulton-Persson et al., 2003; Nulton-Persson and Szweda, 2001). Furthermore, complex II is often missing from respirasome super-complexes where complex I can directly reduce ubiquinone (“Q” in Figure 1 > *EF/cd;* Enriquez and Lenaz, 2014; Hadrava Vanova et al., 2020; Waltz et al., 2024). Regarding the mechanisms of ROS production, Krebs cycle aberration is not a very likely cause since there are various compensatory pathways. For example, SDHB loss in mouse chromaffin cells causes depletion of malate (Figure 1 > *D/d*), but Krebs cycle activity is upheld by pyruvate carboxylase-derived oxaloacetate (“Pcb” in Figure 1 > C/de; Lussey-Lepoutre et al., 2015). The malate-aspartate shuttle (Figure 1 > B/cd; Broeks et al., 2021; Koch et al., 2024), which seems present in *Drosophila* (Curcio et al., 2020; Karp et al., 2017), could even ensure malate dehydrogenase-mediated NADH production (“Mdh2” in Figure 1 > *D/d*). In our view, the most attractive explanation is the suggestion that SDHA loss impairs SDH complex formation in ways that leave the iron-sulphur clusters of SDHB exposed, thus providing a potential source for harmful ROS-production (Hadrava Vanova et al., 2020; Lemarie et al., 2011).

Our results for SesB align with *in vivo* reports for *Drosophila* that its loss causes elevated peroxide levels (Celotto et al., 2006; DeVorkin et al., 2014; Terhzaz et al., 2010; Vartiainen et al., 2014). This is even clearer from mammalian studies. For example, ANT1/2-deficient mouse myoblasts displayed ETC dysfunction as well as reduced glutathione levels and mitochondrial peroxidase activities (Flierl et al., 2022). Knock-down of ANT2 in MCF-7 cells caused significant increase in ROS (Kretova et al., 2014), isolated mitochondria from ANT1-deficient mice had significantly increased hydrogen peroxide production (Esposito et al., 1999), ANT1 overexpression in rat heart protects from ROS damage (Klumpe et al., 2016), and ETC components and activities are upregulated in ANT1/2 double-mutant mouse liver cells (Kokoszka et al., 2004). This said, the ETC was downregulated in ANT1-deficient mouse muscle fibers (Graham et al., 1997) and ANT1 loss was even suggested to be beneficial for cell stresses (Lee et al., 2009), but these studies did not address the potential redundancy of ANT genes. In our view, the most likely cause of ROS production is the dysfunctional ETC in combination with the reduced ability of mitochondrial uncoupling and mPTP impairment, of which the latter is likely to cause calcium stress (Chen Y. et al., 2023) with expected knock-on effects on mitochondrial metabolism including ETC dysregulation (Rossi et al., 2019).

As mentioned before, the potential involvement of ROS in *fh*-linked phenotypes in *Drosophila* is controversially reported and debated (Marelja et al., 2018), but our data clearly demonstrate oxidative stress upon loss of Fh function. Various mechanisms could explain potential ROS increase upon loss of Frataxin. Firstly, the failure of proper iron-sulphur cluster formation will impact on many mitochondrial proteins especially of the ETC which might cause increased electron leakage, hence ROS production. Secondly, Frataxin-deficient mitochondria displayed increased iron levels (Chen et al., 2016; Navarro et al., 2015) which, in turn, inactivated Sod2 with the respective knock-on effects on MTs (Marelja et al., 2018). High iron levels also trigger ferrotopsis-related ROS-inducing processes including the Fenton reaction which generates highly reactive hydroxyl radicals, also observed in Friedreich’s ataxia (Anderson et al., 2008; Costa et al., 2023; La Rosa et al., 2021). In our view, likely all these mechanisms may contribute to our findings.

Our results for Sod2 appear the easiest to explain and align well with very recent data generated in the same *Drosophila* primary neuron system (Shields et al., 2025). Mitochondria of mice lacking SOD2 display reduced respiration, a sensitized transition pore, increased proton leakage, as well as reduced expression of Krebs cycle and ETC enzymes (including SDH and ACONITASE; “Acon” in Figure 1 > *D/f*); knock-out mice display, oxidative stress and genomic DNA damage (Kokoszka et al., 2001; Li et al., 1995; Melov et al., 1998). Increased cytoplasmic ROS levels upon SOD2 loss are likely the consequence of risen intra-mitochondrial superoxide which fails to convert to H_2_O_2_ and reacts with iron-sulphur clusters (Figure 1 > *CD/c*), thus damaging ETC components and enhancing electron leakage (Palma et al., 2020). Further impact may derive from SOD2-mediated production of H_2_O_2_ which is far more diffusive and long-lived than superoxide and a known signaling molecule diffusing to the surrounding cytoplasm (Figure 1 > *A/f*); since SOD2 activity is regulated by the metabolic and redox state of mitochondria it could act as an integrating sensor, and its H_2_O_2_-mediated signaling might help to maintain ROS homeostasis in the surrounding cytoplasm (Palma et al., 2020; Figure 7A).

Taken together, the five MT-curl-inducing conditions (Pdha1, SdhA, Fh, SesB) seem to cause oxidative stress through pathological aberrations of intra-mitochondrial processes leading to leakage of harmful ROS which, in turn, affects MTs in their surrounding. However, of these, Sod2’s potential role as an inducer of beneficial H_2_O_2_-mediated signaling to the surrounding cytoplasm (Palma et al., 2020) might mean that not only Sod2 deficiency, but also the absence of entire mitochondria would deprive axons of this signaling and destabilize ROS homeostasis (Figure 7C). In our view, Sod2 is therefore a potential candidate that might explain MT-curling in the absence of mitochondrial transport (see section “Introduction”) (Liew et al., 2025).

### Non-curl-inducing conditions

Eight out of 13 gene deficiencies failed to induce MT-curling. Functional loss of ATPsynC or YME1L even showed signs of reduced MT-curling (Figures 3G,H) suggesting a potential drop in ROS levels below baseline as was discussed.

In agreement with our findings for Ogdh1, also OGDHL-deficient human neuroblastoma cells were shown to reduce oxygen consumption (Yap et al., 2021a). OGDH loss affects the Krebs cycle with downstream effects like causing mTORC1 activation (Yoon et al., 2017), although cycle activity can be partly maintained: for example via import of malate (Figure 1 > AB/c; Allen et al., 2016; Yoon et al., 2017) or via methionine catabolism to generate succinyl-CoA (not shown; Yap et al., 2021a). Notably, OGDH was shown to be inactivated through glutathionylation in response to heightened H_2_O_2_ levels, thus acting as a ROS sensor that can down-regulate NADH production and OXPHOS (Applegate et al., 2008; Nulton-Persson et al., 2003; Nulton-Persson and Szweda, 2001). Loss of Ogdh1 might mimic this silencing effect, expected to cause a reduction or at least no increase in ROS production, as seen in our studies.

Consistent with the lack of MT-curling we observed upon QIL1 knock-down, also MICOS13-deficient cells were reported to display reduced oxygen consumption (Guarani et al., 2015; Kishita et al., 2020) and no studies seem to suggest ROS involvement (Gödiker et al., 2018; Guarani et al., 2016; Russell et al., 2019; Zeharia et al., 2016).

Matters are less clear for the fission and fusion factors. In *Drosophila*, Drp1 loss decreases lifespan (Rana et al., 2017), but there seem to be no reports of ROS-induced neurodegeneration; instead Drp1 deficiency was shown to rescue longevity in a proteasome-deficient model (Tsakiri et al., 2019b), and dominant-negative Drp1 was beneficial in ALS models (Altanbyek et al., 2016). The *Opa1*^*s*3475^ mutant allele was shown to cause elevated ROS, shorter lifespan and necrosis of support cells in the fly eye (Tang et al., 2009; Yarosh et al., 2008), and Opa1 knock-down caused axon degeneration (Cao et al., 2017). In other reports, *Opa1*^*s*3475^ reduced toxicity in a *Drosophila* Alzheimer model (DuBoff et al., 2012), and *Opa1* knock-down in muscles increased lifespan and improved locomotor activity (Tapia et al., 2021). Similarly, functional loss of Marf caused precocious axon degeneration (Cao et al., 2017), enhances decline in spastic paraplegia models (Fowler and O’Sullivan, 2016), triggers ROS-increase in nephrocytes (Zhu et al., 2024) and leads to ER stress and fragmentation (Debattisti et al., 2014). But it was also shown to rescue frataxin-induced glial degeneration (Edenharter et al., 2018), alleviate ROS-mediated rhabdomere degeneration in Huntington’s disease models (Campesan et al., 2023), reduce pink/parkin-induced ER stress in a fly Parkinson model (Celardo et al., 2016; but see Basso et al., 2018) and increase lifespan and locomotor activity (Rana et al., 2017; Tapia et al., 2021). Also in mammalian studies, some report ROS increase upon mitochondrial fragmentation caused by OPA1 or MFN loss (Ježek et al., 2018; Millet et al., 2016; Zhang et al., 2017), whereas others report that DNM1L loss causes proton leakage and a reduction in ROS (Galloway et al., 2012; Kolac et al., 2023), that OPA1-deficient mouse embryonic fibroblasts and hepatocytes have reduced ROS (Lee et al., 2023; Liang et al., 2024), that MFN1-deficient myocytes display normal mitochondrial physiology and improved ROS tolerance (Papanicolaou et al., 2012), and that loss of MFN2 in macrophages have decreased ROS production (Tur et al., 2020). Our experiments for all these factors consistently show lack of MT-curling arguing against ROS increase. We propose that local maintenance mechanisms including mitochondrial protease systems and mitochondria-derived vesicles might be able to keep mitochondrial physiology in balance for an extended period (Misgeld and Schwarz, 2017).

Lack of MT-curling upon functional deficiency of ATP synthase aligns with findings in the literature. For example, blocking mouse ATP synthase affects respirasome assembly and metabolically protects neurons against cellular stresses (Formentini et al., 2014; García-Aguilar and Cuezva, 2018), and cells carrying the dystonia-linked ATP5MC3^*N*106*K*^ mutation display reduced ATP production and oxygen consumption (Neilson et al., 2022). The surprising survival of *mt:ATPase6^1^* mutant individuals into adult flies was explained by mitochondrial uncoupling (Demine et al., 2019) and glycolytic ATP production (Figure 1 > A/de; Celotto et al., 2011), none of which would suggest ROS production. However, ROS overproduction has been reported for neurodegeneration-linked human MT-ATP6 mutations (Dautant et al., 2018; Galber et al., 2021), in one case involving overproduction of SOD1 and 2 (Geromel et al., 2001). Such effects might relate to roles of the ATP synthase in forming the high-conductance mitochondrial permeability transition pore (mPTP; Bonora et al., 2022) causing late-onset effects not covered by our experimental schedule.

As explained in the Results section, also loss of Yme1L causes late onset of ROS and neurodegeneration in fly and mouse alike, whereas we find that loss of Yme1L causes a strong reduction of MT-curling at 5 DIV. Our results are perhaps best explained by findings in yeast, where loss of *Yme1* causes a severe drop in levels of various ETC components expected to decrease activities of complexes II, III and IV (Kan et al., 2022) and, hence, reduce ROS production. We propose therefore that the loss of Yme1 protease activity causes reduced processing of ETC components leading to early ROS reduction, whereas its roles in mitochondrial quality control, i.e., to remove protein aggregates, become more relevant over a longer time period, potentially masked by other maintenance mechanisms including the shedding of mitochondria-derived vesicles (Held and Houtkooper, 2015; Mattedi et al., 2023; Misgeld and Schwarz, 2017), thus explaining late-onset ROS not seen in our cultures.

### Conclusions and future directions

The approach taken here was clearly able to answer the posed questions regarding the ROS-mediated impact of mitochondrial dysfunction on MT bundles. This suggests axonal MT bundles as potential downstream targets in mitochondrial pathology, that would provide a mechanism for axon degeneration. Our data might also suggest Sod2 as a potential candidate explaining MT bundle deterioration upon mitochondrial absence. Further thought-provoking observations were made, such as the absence of MT-curling when affecting mitochondrial morphogenesis, or the strong reduction of curling upon loss of QIL1. Importantly, the *Drosophila* primary neuron system is well-suited to validate findings and test deduced hypotheses efficiently, for example using double- or triple-mutant conditions to clarify compensatory metabolic pathways. Furthermore, findings can be easily validated *in vivo* using the same genetic tools as in culture. This provides promising means to refine our understanding of the role of mitochondria in axons.

## Methods

### Genetic strategies and fly lines

The wild-type control used throughout the project was the *Drosophila melanogaster* Oregan R strain. Most mutant or transgenic fly stocks were sourced from the Bloomington *Drosophila* Stock Centre (BDSC stock number provided in brackets). Loss-of-function mutant strains were (in alphabetical order): *ATPsynC^KO^* (*ATPsynC*^*KG*01914^; P-element insertion in non-coding 5’ exon generating a protein null; BDSC#13923; Lovero et al., 2018); *ATPsynC^Df^* (*Df(3R)Exel6218*; uncovering ATPsynC; BDSC#7696; Parks et al., 2004); *ATP6^KO^* (*mt:ATPase6^1^*; lethal G116E point mutation; BDSC#95253; Celotto et al., 2006); *Drp1^KO^* (*Drp1*^*T*26^; lethal allele; BDSC#3662; Verstreken et al., 2005); *fh^KO^* (*fh*^1^; lethal S136R point mutation; BDSC#67161; Chen et al., 2016); *Marf^KO^* (*Marf^B^*; amorphic allele; BDSC#67154; Sandoval et al., 2014); *Ogdh1^Df^* (*Df(3L)Exel7253*; BDSC#7938; Ryder et al., 2007); *Odgh1^KO^* (*Mi{Trojan-GAL4.1}Ogdh1*^*MI*06026–TG4.1^ aka *dOgdh-T2A-Gal4*; BDSC#77497; Yoon et al., 2017); *Opa1^KO^* (*Opa1*^*s*3475^ aka *Opa1*^*ex*2^; BL #12188; strong loss of function due to P-element insertion; Spradling et al., 1999; Yarosh et al., 2008); *Pdha1^KO^* (*Pdha1^A^* aka *l(1)G0334A*; lethal G126E point mutation; BDSC#52370; Yamamoto et al., 2014); *SdhA*^1404^ (lethal V445E point mutation; BDSC#81120; Mast et al., 2008); *SdhA*^1110^ (lethal E288K point mutation; BDSC#51659; Mast et al., 2008); *Sod2^KO^* (*Sod2*^*n*283^; BL#34060; 167bp deletion removing part of the first exon and intron; Duttaroy et al., 2003); *YME1L^KO^* (*YME1L^del^*; 2kb deletion removing most of the coding region; BDSC#95273; Qi et al., 2016). Knock-down experiments were performed using the Gal4/UAS system (Elliott and Brand, 2008) employing the second-chromosomal *elav-Gal4* driver (BDSC #8765) in combination with the following transgenic UAS constructs: *fh^IR^* (*P{UAS-fh.RNAi.A}2*; BDSC#24620; Anderson et al., 2005); *Marf^IR^* (*HMC03883*; BL#55189; Perkins et al., 2015); *Opa1^IR^* (*HMS00349*; BDSC#32358; Perkins et al., 2015); *Pdha^IR^* (*P{TRiP.HMC04032}*; BDSC#55345; Perkins et al., 2015); *QIL1^IR^* (*P{TRiP.GLC01383}*; expression reduced to about 25%; loss of cristae junctions; BDSC#44364; Guarani et al., 2015; Perkins et al., 2015); *sesB*^*IR*1^ (*P{TRiP.HMS01549};* BDSC#36661; Perkins et al., 2015); *sesB*^*IR*2^ (*P{TRiP.JF01528};* BDSC#31077; Perkins et al., 2015). Green balancer chromosomes used to identify mutant or construct-expressing embryos were readily available *FM7*, *CyO or TM3* balancers carrying *twi-Gal4 or Kr-Gal4* in combination with *UAS-GFP* or carrying a *Df-GFP* fusion construct (Casso et al., 2000; Halfon et al., 2002; Le et al., 2006).

### *Drosophila* primary cell culture

*Drosophila* primary neuron cultures were performed as published previously (Prokop et al., 2012; Voelzmann and Sánchez-Soriano, 2022). In brief, stage 11 embryos were treated for 1 min with bleach to remove the chorion, sterilized for ∼30 s in 70% ethanol, washed in sterile Schneider’s/FCS, and eventually homogenized with micro-pestles in 1.5 ml centrifuge tubes containing 21 embryos per 100 μl dispersion medium and left to incubate for 5 min at 37°C. Cells were washed with Schneider’s medium (Gibco), spun down for 4 mins at 650 g, supernatant was removed and cells re-suspended in 90 μl of Schneider’s medium containing 20% fetal calf serum (Gibco). 30 μl drops were placed on cover slips. Cells were allowed to adhere for ∼2 hrs on cover slips coated with a 5 μg/ml solution of concanavalin A and then grown as a hanging drop culture for several days at 26°C as indicated in each experiment as days *in vitro* (DIV). To abolish maternal product deposited by heterozygous mothers in their oocytes (Prokop, 2013), we used a pre-culture strategy (Prokop et al., 2012; Sánchez-Soriano et al., 2010) where cells were kept for 5 days in a tube before they were plated on a coverslip [indicated as “(pre)” in Figure 3].

In some experiments, cells were treated with 100 μM Trolox (Sigma) throughout the entire culture period; to make pre-dilutions, Trolox solid was dissolved in culture medium (using rigorous shaking and warming to 37°C) achieving 10 mM concentration; the pre-dilution was then further diluted 1:100 in culture medium, sterile-filtered and used to culture the cells.

### Staining procedures

Cultured neurons on coverslip were fixed for 30 min using a drop of 4% paraformaldehyde (PFA) and 0.05% glutardialdehyde in 0.05M phosphate buffer (PB; pH 7–7.2). Cells were washed with 0.5% Tergitol solution in 0.05M PB for 20 min (one exchange). The cells were incubated for 2 hrs in a 200 μl drop of anti-tubulin primary antibodies diluted 1:500 in PB (clone DM1A, mouse, Sigma or clone YOL1/34, rat, Antibodies.com). Cells were washed with PBS and then incubated for 1.5 h with secondary FITC- or Cy3-conjugated anti-mouse antibodies (donkey, Jackson ImmunoResearch, 1:200 in PBS). To image cell morphology and identify neurons, cells were co-labelled with TRITC-, Alexa647-, FITC- or Atto647N-conjugated anti-HRP (goat, Jackson Immuno Research, 1:100). For Eb1 comet analysis, cells were fixed for 10 min in the freezer with pre-cooled Plus-Tip Fixative (90% methanol, 3% formaldehyde, 5 mM sodium carbonate, pH 9), then washed with PBS and stained as described above with anti-DmEb1 (gift from H. Ohkura; rabbit, 1:500; Elliott et al., 2005). For the visualization of mitochondria, cell cultures were incubated with 1 μM MitoTracker Red CMXRos (Invitrogen; Klionsky et al., 2012) for 30 min at room temperature (RT); stock solutions were prepared from 50 μg lyophilized solid dissolved in 94 μl of DMSO of which 2 μl were added to 2 ml of growth medium. Following incubation, cultures were then fixed and stained following the procedures below. Specimens were embedded in ProLong Gold Antifade mounting medium (Invitrogen) on microscope slides. The embedded slides were left to dry overnight in the dark before imaging.

### Imaging and data analysis

Neurons were visualized using a compound fluorescence microscope (BX50WI or BX51; Olympus) and images of single neurons were captured using nijiBlueBox and the MatrixVision mvBlueFox3-M2 2124G camera at 100x magnification. Images were analysed using the FIJI/ImageJ 1.54p software. To determine the degree of MT disorganization in axons we used the “MT disorganization index” (MDI; Qu et al., 2017): the axon length (from cell body to tip of the most distant microtubule) was measured using the segmented line tool; area of disorganization was measured using the freehand selection tool; this value was then divided by the the product of axon length multiplied with 0.5 μm (arbitrary axon diameter, thus approximating the expected area of the axon if it were not disorganized); for axon branching data, primary neurites containing a microtubule core of at least 10 μm and branching off the longest neurite were counted. For the Eb1 comet analysis, length and mean intensity of the Eb1 comets were measured using the line tool in FIJI; Eb1 amount was calculated by multiplying comet length with mean intensity (Hahn et al., 2021). All data were normalized against their respective controls. In each experiment, usually three slides per genotype were analysed aiming to image ∼30 isolated neurons per slide. Experiments were repeated at least once, data pooled. MDI data were usually not normally distributed but nevertheless plotted as mean ± SEM to avoid misleading median values of zero. Most experiments had only two groups and were assessed using Mann–Whitney Rank Sum tests, experiments with more then two group using Kruskal–Wallis one-way ANOVA with *post hoc* Dunn’s test. Means of single slides were used to generate super-plots (Lord et al., 2020) and assessed using standard *t-tests*. Statistical analyses were performed with Graphpad 10.2.2. The data used for our analyses will be made available on request. Image plates were generated with Photoshop CS6 and illustrations with Illustrator CS.

## References

[B1] Al-Mahdawi S. Pinto R. M. Varshney D. Lawrence L. Lowrie M. B. Hughes S. (2006). GAA repeat expansion mutation mouse models of *Friedreich ataxia* exhibit oxidative stress leading to progressive neuronal and cardiac pathology. *Genomics* 88 580–590. 10.1016/j.ygeno.2006.06.015 16919418 PMC2842930

[B2] Al-Rasheed M. R. H. Tarjan G. (2018). Succinate dehydrogenase complex: An updated review. *Arch. Pathol. Lab. Med.* 142 1564–1570. 10.5858/arpa.2017-0285-RS 30289269

[B3] Allen E. L. Ulanet, Danielle B. Pirman D. Mahoney, Christopher E. (2016). Differential aspartate usage identifies a subset of cancer cells particularly dependent on OGDH. *Cell Rep.* 17 876–890. 10.1016/j.celrep.2016.09.052 27732861

[B4] Altanbyek V. Cha S.-J. Kang G.-U. Im D. S. Lee S. Kim H.-J. (2016). Imbalance of mitochondrial dynamics in *Drosophila* models of amyotrophic lateral sclerosis. *Biochem. Biophys. Res. Commun.* 481 259–264. 10.1016/j.bbrc.2016.10.134 27810362

[B5] Anderson P. R. Kirby K. Hilliker A. J. Phillips J. P. (2005). RNAi-mediated suppression of the mitochondrial iron chaperone, frataxin, in *Drosophila*. *Hum. Mol. Genet.* 14 3397–3405. 10.1093/hmg/ddi367 16203742

[B6] Anderson P. R. Kirby K. Orr W. C. Hilliker A. J. Phillips J. P. (2008). Hydrogen peroxide scavenging rescues frataxin deficiency in a *Drosophila* model of Friedreich’s ataxia. *Proc. Natl. Acad. Sci. U. S. A.* 105 611–616. 10.1073/pnas.0709691105 18184803 PMC2206584

[B7] Andreyev A. Y. Kushnareva Y. E. Starkov A. A. (2005). Mitochondrial metabolism of reactive oxygen species. *Biochem (Moscow)* 70 200–214. 10.1007/s10541-005-0102-7 15807660

[B8] Applegate M. A. B. Humphries K. M. Szweda L. I. (2008). Reversible inhibition of α-ketoglutarate dehydrogenase by hydrogen peroxide: Glutathionylation and protection of lipoic acid. *Biochem* 47 473–478. 10.1021/bi7017464 18081316

[B9] Basso V. Marchesan E. Peggion C. Chakraborty J. von Stockum S. Giacomello M. (2018). Regulation of ER-mitochondria contacts by Parkin via Mfn2. *Pharmacol. Res.* 138 43–56. 10.1016/j.phrs.2018.09.006 30219582

[B10] Berthet A. Margolis E. B. Zhang J. Hsieh I. Zhang J. Hnasko T. S. (2014). Loss of mitochondrial fission depletes axonal mitochondria in midbrain dopamine neurons. *J. Neurosci.* 34 14304–14317. 10.1523/JNEUROSCI.0930-14.2014 25339743 PMC4205554

[B11] Bhattacharya D. Azambuja A. P. Simoes-Costa M. (2020). Metabolic reprogramming promotes neural crest migration via Yap/Tead signaling. *Dev. Cell* 53 199–211.e6. 10.1016/j.devcel.2020.03.005. 32243782 PMC7236757

[B12] Bonora M. Giorgi C. Pinton P. (2022). Molecular mechanisms and consequences of mitochondrial permeability transition. *Nat. Rev. Mol. Cell Biol.* 23 266–285. 10.1038/s41580-021-00433-y 34880425

[B13] Borcherding N. Brestoff J. R. (2023). The power and potential of mitochondria transfer. *Nature* 623 283–291. 10.1038/s41586-023-06537-z 37938702 PMC11590279

[B14] Brischigliaro M. Fernandez-Vizarra E. Viscomi C. (2023). Mitochondrial neurodegeneration: Lessons from *Drosophila melanogaster* models. *Biomolecules* 13:378. 10.3390/biom13020378 36830747 PMC9953451

[B15] Broeks M. H. van Karnebeek C. D. M. Wanders R. J. A. Jans J. J. M. Verhoeven-Duif N. M. (2021). Inborn disorders of the malate aspartate shuttle. *J. Inherit. Metab. Dis.* 44 792–808. 10.1002/jimd.12402 33990986 PMC8362162

[B16] Bround M. J. Bers D. M. Molkentin J. D. (2020). A 20/20 view of ANT function in mitochondrial biology and necrotic cell death. *J. Mol. Cell Cardiol.* 144 A3–A13. 10.1016/j.yjmcc.2020.05.012 32454061 PMC7483807

[B17] Campesan S. del Popolo I. Marcou K. Straatman-Iwanowska A. Repici M. Boytcheva K. V. (2023). Bypassing mitochondrial defects rescues Huntington’s phenotypes in *Drosophila*. *Neurobiol. Dis.* 185:106236. 10.1016/j.nbd.2023.106236 37495179

[B18] Cao X. Wang H. Wang Z. Wang Q. Zhang S. Deng Y. (2017). In vivo imaging reveals mitophagy independence in the maintenance of axonal mitochondria during normal aging. *Aging Cell* 16 1180–1190. 10.1111/acel.12654 28782874 PMC5595681

[B19] Casso D. Ramirez-Weber F. Kornberg T. B. (2000). GFP-tagged balancer chromosomes for *Drosophila melanogaster*. *Mech. Dev.* 91 451–454. 10.1016/s0925-4773(00)00248-3 10704882

[B20] Celardo I. Costa A. C. Lehmann S. Jones C. Wood N. Mencacci N. E. (2016). Mitofusin-mediated ER stress triggers neurodegeneration in pink1/parkin models of Parkinson’s disease. *Cell Death Dis.* 7:e2271. 10.1038/cddis.2016.173 27336715 PMC5143399

[B21] Celotto A. M. Chiu W. K. Van Voorhies W. Palladino M. J. (2011). Modes of metabolic compensation during mitochondrial disease using the *Drosophila* model of ATP6 dysfunction. *PLoS One* 6:e25823. 10.1371/journal.pone.0025823 21991365 PMC3185040

[B22] Celotto A. M. Frank A. C. McGrath S. W. Fergestad T. Voorhies W. A. V. Buttle K. F. (2006). Mitochondrial encephalomyopathy in *Drosophila*. *J. Neurosci.* 26 810–820. 10.1523/jneurosci.4162-05.2006 16421301 PMC6675365

[B23] Chapp A. D. Behnke J. E. Driscoll K. M. Hahka T. LaLonde Z. Shan Z. (2021). Elevated L-lactate promotes major cellular pathologies associated with neurodegenerative diseases. *Neurosci. Bull.* 37 380–384. 10.1007/s12264-020-00611-6 33210187 PMC7955001

[B24] Chen K. Lin G. Haelterman N. A. Ho T. S.-Y. Li T. Li Z. (2016). Loss of Frataxin induces iron toxicity, sphingolipid synthesis, and Pdk1/Mef2 activation, leading to neurodegeneration. *eLife* 5:e16043. 10.7554/eLife.16043 27343351 PMC4956409

[B25] Chen W. Zhao H. Li Y. (2023). Mitochondrial dynamics in health and disease: Mechanisms and potential targets. *Signal Transduct. Target Ther.* 8:333. 10.1038/s41392-023-01547-9 37669960 PMC10480456

[B26] Chen Y. Wu L. Liu J. Ma L. Zhang W. (2023). Adenine nucleotide translocase: Current knowledge in post-translational modifications, regulations and pathological implications for human diseases. *FASEB J.* 37:e22953. 10.1096/fj.202201855RR 37224026

[B27] Chow H. S. Lynch J. J. Rose K. Choi D. W. (1994). Trolox attenuates cortical neuronal injury induced by iron, ultraviolet light, glucose deprivation, or AMPA. *Brain Res.* 639 102–108. 10.1016/0006-8993(94)91769-8 7514085

[B28] Conze C. Trushina N. I. Monteiro-Abreu N. Singh L. Romero D. V. Wienbeuker E. (2025). Redox signaling modulates axonal microtubule organization and induces a specific phosphorylation signature of microtubule-regulating proteins. *Redox Biol.* 83:103626. 10.1016/j.redox.2025.103626 40222271 PMC12019850

[B29] Costa I. Barbosa D. J. Silva V. Benfeito S. Borges F. Remião F. (2023). Research models to study ferroptosis’s impact in neurodegenerative diseases. *Pharmaceutics* 15:1369. 10.3390/pharmaceutics15051369 37242612 PMC10224233

[B30] Curcio R. Lunetti P. Zara V. Ferramosca A. Marra F. Fiermonte G. (2020). *Drosophila melanogaster* mitochondrial carriers: Similarities and differences with the human carriers. *Int. J. Mol. Sci.* 21:6052. 10.3390/ijms21176052 32842667 PMC7504413

[B31] D’Angelo D. Vecellio Reane D. Raffaello A. (2023). Neither too much nor too little: Mitochondrial calcium concentration as a balance between physiological and pathological conditions. *Front. Mol. Biosci.* 10:1336416. 10.3389/fmolb.2023.1336416 38148906 PMC10749936

[B32] Dautant A. Meier T. Hahn A. Tribouillard-Tanvier D. di Rago J.-P. Kucharczyk R. (2018). ATP synthase diseases of mitochondrial genetic origin. *Front. Physiol.* 9:329. 10.3389/fphys.2018.00329 29670542 PMC5893901

[B33] Debattisti V. Pendin D. Ziviani E. Daga A. Scorrano L. (2014). Reduction of endoplasmic reticulum stress attenuates the defects caused by *Drosophila mitofusin* depletion. *J. Cell Biol.* 204 303–312. 10.1083/jcb.201306121 24469638 PMC3912536

[B34] Delatycki M. B. Williamson R. Forrest S. M. (2000). Friedreich ataxia: An overview. *J. Med. Genet.* 37 1–8. 10.1136/jmg.37.1.1 10633128 PMC1734457

[B35] Demine S. Renard P. Arnould T. (2019). Mitochondrial uncoupling: A key controller of biological processes in physiology and diseases. *Cells* 8:795. 10.3390/cells8080795 31366145 PMC6721602

[B36] DeVorkin L. Go N. E. Hou Y.-C. C. Moradian A. Morin G. B. Gorski S. M. (2014). The *Drosophila* effector caspase Dcp-1 regulates mitochondrial dynamics and autophagic flux via SesB. *J. Cell Biol.* 205 477–492. 10.1083/jcb.201303144 24862573 PMC4033768

[B37] Dias-Santagata D. Fulga T. A. Duttaroy A. Feany M. B. (2007). Oxidative stress mediates tau-induced neurodegeneration in *Drosophila*. *J. Clin. Invest.* 117 236–245. 10.1172/JCI28769 17173140 PMC1697799

[B38] DuBoff B. Gotz J. Feany M. B. (2012). Tau promotes neurodegeneration via DRP1 mislocalization in vivo. *Neuron* 75 618–632. 10.1016/j.neuron.2012.06.026 22920254 PMC3428596

[B39] Dung V. M. Suong D. N. A. Okamaoto Y. Hiramatsu Y. Thao D. T. P. Yoshida H. (2018). Neuron-specific knockdown of *Drosophila* PDHB induces reduction of lifespan, deficient locomotive ability, abnormal morphology of motor neuron terminals and photoreceptor axon targeting. *Exp. Cell Res.* 366 92–102. 10.1016/j.yexcr.2018.02.035 29501567

[B40] Duttaroy A. Paul A. Kundu M. Belton A. (2003). A Sod2 null mutation confers severely reduced adult life span in *Drosophila*. *Genetics* 165 2295–2299. 10.1093/genetics/165.4.2295 14704205 PMC1462872

[B41] Edenharter O. Schneuwly S. Navarro J. A. (2018). Mitofusin-dependent ER stress triggers glial dysfunction and nervous system degeneration in a *Drosophila* model of Friedreich’s ataxia. *Front. Mol. Neurosci.* 11:38. 10.3389/fnmol.2018.00038 29563863 PMC5845754

[B42] Elliott D. A. Brand A. H. (2008). The GAL4 system: A versatile system for the expression of genes. *Drosophila*. *Methods Protocols* 420 79–95. 10.1007/978-1-59745-583-1_5 18641942

[B43] Elliott S. L. Cullen C. F. Wrobel N. Kernan M. J. Ohkura H. (2005). EB1 is essential during *Drosophila* development and plays a crucial role in the integrity of chordotonal mechanosensory organs. *Mol. Biol. Cell* 16 891–901. 10.1091/mbc.e04-07-0633 15591130 PMC545920

[B44] Else P. L. (2020). The highly unnatural fatty acid profile of cells in culture. *Prog. Lipid Res.* 77:101017. 10.1016/j.plipres.2019.101017 31809755

[B45] Enriquez J. A. Lenaz G. (2014). Coenzyme Q and the respiratory chain: Coenzyme Q pool and mitochondrial supercomplexes. *Mol. Syndromol.* 5 119–140. 10.1159/000363364 25126045 PMC4112531

[B46] Esposito L. A. Melov S. Panov A. Cottrell B. A. Wallace D. C. (1999). Mitochondrial disease in mouse results in increased oxidative stress. *Proc. Natl. Acad. Sci. U. S. A.* 96 4820–4825. 10.1073/pnas.96.9.4820 10220377 PMC21775

[B47] Fan X. Barshop W. D. Vashisht A. A. Pandey V. Leal S. Rayatpisheh S. (2022). Iron-regulated assembly of the cytosolic iron-sulfur cluster biogenesis machinery. *J. Biol. Chem.* 298:102094. 10.1016/j.jbc.2022.102094 35654137 PMC9243173

[B48] Flierl A. Schriner S. E. Hancock S. Coskun P. E. Wallace D. C. (2022). The mitochondrial adenine nucleotide transporters in myogenesis. *Free Rad. Biol. Med.* 188 312–327. 10.1016/j.freeradbiomed.2022.05.022 35714845

[B49] Flynn J. M. Melov S. (2013). SOD2 in mitochondrial dysfunction and neurodegeneration. *Free Rad. Biol. Med.* 62 4–12. 10.1016/j.freeradbiomed.2013.05.027 23727323 PMC3811078

[B50] Formentini L. Pereira M. P. Sánchez−Cenizo L. Santacatterina F. Lucas J. J. Navarro C. (2014). In vivo inhibition of the mitochondrial H+-ATP synthase in neurons promotes metabolic preconditioning. *EMBO J.* 33 762–778. 10.1002/embj.201386392 24521670 PMC4000092

[B51] Foucher C. Tubben R. (2024). Lactic acidosis [Updated 2023 Jul 17]. In: *StatPearls [Internet]*. Treasure Island, FL: StatPearls Publishing. Available online at: https://www.ncbi.nlm.nih.gov/books/NBK470202

[B52] Fowler P. C. O’Sullivan N. C. (2016). ER-shaping proteins are required for ER and mitochondrial network organization in motor neurons. *Hum. Mol. Genet.* 25 2827–2837. 10.1093/hmg/ddw139 27170313

[B53] Galber C. Carissimi S. Baracca A. Giorgio V. (2021). The ATP synthase deficiency in human diseases. *Life* 11:325. 10.3390/life11040325 33917760 PMC8068106

[B54] Galloway C. A. Lee H. Nejjar S. Jhun B. S. Yu T. Hsu W. (2012). Transgenic control of mitochondrial fission induces mitochondrial uncoupling and relieves diabetic oxidative stress. *Diabetes* 61 2093–2104. 10.2337/db11-1640 22698920 PMC3402299

[B55] García-Aguilar A. Cuezva J. M. (2018). A review of the inhibition of the mitochondrial ATP synthase by IF1 in vivo: Reprogramming energy metabolism and inducing mitohormesis. *Front. Physiol.* 9:1322. 10.3389/fphys.2018.01322 30283362 PMC6156145

[B56] GBD 2016 Neurology Collaborators, (2019). Global, regional, and national burden of neurological disorders, 1990–2016: A systematic analysis for the global burden of disease study 2016. *Lancet Neurol.* 18 459–480. 10.1016/S1474-4422(18)30499-X 30879893 PMC6459001

[B57] Geromel V. Kadhom N. Cebalos-Picot I. Ouari O. Polidori A. Munnich A. (2001). Superoxide-induced massive apoptosis in cultured skin fibroblasts harboring the neurogenic ataxia retinitis pigmentosa (NARP) mutation in the ATPase-6 gene of the mitochondrial DNA. *Hum. Mol. Genet.* 10 1221–1228. 10.1093/hmg/10.11.1221 11371515

[B58] Glover H. L. Schreiner A. Dewson G. Tait S. W. G. (2024). Mitochondria and cell death. *Nat. Cell Biol.* 26 1434–1446. 10.1038/s41556-024-01429-4 38902422

[B59] Glushakova L. G. Judge S. Cruz A. Pourang D. Mathews C. E. Stacpoole P. W. (2011). Increased superoxide accumulation in pyruvate dehydrogenase complex deficient fibroblasts. *Mol. Genet. Metab.* 104 255–260. 10.1016/j.ymgme.2011.07.023 21846590 PMC3205311

[B60] Gödiker J. Grüneberg M. DuChesne I. Reunert J. Rust S. Westermann C. (2018). QIL1-dependent assembly of MICOS complex–lethal mutation in C19ORF70 resulting in liver disease and severe neurological retardation. *J. Hum. Genet.* 63 707–716. 10.1038/s10038-018-0442-y 29618761

[B61] Goffrini P. Ercolino T. Panizza E. Giachè V. Cavone L. Chiarugi A. (2009). Functional study in a yeast model of a novel succinate dehydrogenase subunit B gene germline missense mutation (C191Y) diagnosed in a patient affected by a glomus tumor. *Hum. Mol. Genet.* 18 1860–1868. 10.1093/hmg/ddp102 19261679

[B62] Gonçalves-Pimentel C. Gombos R. Mihály J. Sánchez-Soriano N. Prokop A. (2011). Dissecting regulatory networks of filopodia formation in a *Drosophila* growth cone model. *PLoS One* 6:e18340. 10.1371/journal.pone.0018340 21464901 PMC3065487

[B63] Gopal K. Abdualkader A. M. Li X. Greenwell A. A. Karwi Q. G. Altamimi T. R. (2023). Loss of muscle PDH induces lactic acidosis and adaptive anaplerotic compensation via pyruvate-alanine cycling and glutaminolysis. *J. Biol. Chem.* 299:105375. 10.1016/j.jbc.2023.105375 37865313 PMC10692893

[B64] Graham B. H. Waymire K. G. Cottrell B. Trounce I. A. MacGregor G. R. Wallace D. C. (1997). A mouse model for mitochondrial myopathy and cardiomyopathy resulting from a deficiency in the heart/muscle isoform of the adenine nucleotide translocator. *Nat. Genet.* 16 226–234. 10.1038/ng0797-226 9207786

[B65] Guarani V. Jardel C. Chrétien D. Lombès A. Bénit P. Labasse C. (2016). QIL1 mutation causes MICOS disassembly and early onset fatal mitochondrial encephalopathy with liver disease. *eLife* 5:e17163. 10.7554/eLife.17163 27623147 PMC5021520

[B66] Guarani V. McNeill E. M. Paulo J. A. Huttlin E. L. Fröhlich F. Gygi S. P. (2015). QIL1 is a novel mitochondrial protein required for MICOS complex stability and cristae morphology. *eLife* 4:e06265. 10.7554/eLife.06265 25997101 PMC4439739

[B67] Guo H. Bueler S. A. Rubinstein J. L. (2017). Atomic model for the dimeric F0 region of mitochondrial ATP synthase. *Science* 358 936–940. 10.1126/science.aao4815 29074581 PMC6402782

[B68] Guzy R. D. Sharma B. Bell E. Chandel N. S. Schumacker P. T. (2008). Loss of the SdhB, but not the SdhA subunit of complex II triggers reactive oxygen species-dependent hypoxia-inducible factor activation and tumorigenesis. *Mol. Cell Biol.* 28 718–731. 10.1128/MCB.01338-07 17967865 PMC2223429

[B69] Hadrava Vanova K. Kraus M. Neuzil J. Rohlena J. (2020). Mitochondrial complex II and reactive oxygen species in disease and therapy. *Redox Rep.* 25 26–32. 10.1080/13510002.2020.1752002 32290794 PMC7178880

[B70] Hahn A. Parey K. Bublitz M. Mills, Deryck J. Zickermann V. (2016). Structure of a complete ATP synthase dimer reveals the molecular basis of inner mitochondrial membrane morphology. *Mol. Cell* 63 445–456. 10.1016/j.molcel.2016.05.037 27373333 PMC4980432

[B71] Hahn I. Voelzmann A. Parkin J. Fuelle J. B. Slater P. G. Lowery L. A. (2021). Tau, XMAP215 and Eb co-operatively regulate microtubule polymerisation and bundle formation in axons. *PLoS Genet.* 17:e1009647. 10.1371/journal.pgen.1009647 34228717 PMC8284659

[B72] Halfon M. S. Gisselbrecht S. Lu J. Estrada B. Keshishian H. Michelson A. M. (2002). New fluorescent protein reporters for use with the *Drosophila* Gal4 expression system and for vital detection of balancer chromosomes. *Genesis* 34 135–138. 10.1002/gene.10136 12324968

[B73] Hartmann B. Wai T. Hu H. MacVicar T. Musante L. Fischer-Zirnsak B. (2016). Homozygous YME1L1 mutation causes mitochondriopathy with optic atrophy and mitochondrial network fragmentation. *eLife* 5:e16078. 10.7554/eLife.16078 27495975 PMC4991934

[B74] Held N. M. Houtkooper R. H. (2015). Mitochondrial quality control pathways as determinants of metabolic health. *BioEssays* 37 867–876. 10.1002/bies.201500013 26010263 PMC5053262

[B75] Hirabayashi Y. Lewis T. L. Du Y. Virga D. M. Decker A. M. Coceano G. (2024). Most axonal mitochondria in cortical pyramidal neurons lack mitochondrial DNA and consume ATP. *bioRxiv [Preprint]* 10.1101/2024.02.12.579972 38405915 PMC10888904

[B76] Houldsworth A. (2024). Role of oxidative stress in neurodegenerative disorders: A review of reactive oxygen species and prevention by antioxidants. *Brain Commun.* 6:fcad356. 10.1093/braincomms/fcad356 38214013 PMC10783645

[B77] Huang Y. Wan Z. Tang Y. Xu J. Laboret B. Nallamothu S. (2022). Pantothenate kinase 2 interacts with PINK1 to regulate mitochondrial quality control via acetyl-CoA metabolism. *Nat. Commun.* 13:2412. 10.1038/s41467-022-30178-x 35504872 PMC9065001

[B78] Ishii T. Miyazawa M. Onodera A. Yasuda K. Kawabe N. Kirinashizawa M. (2011). Mitochondrial reactive oxygen species generation by the SDHC V69E mutation causes low birth weight and neonatal growth retardation. *Mitochondrion* 11 155–165. 10.1016/j.mito.2010.09.006 20870041

[B79] Jaiswal M. Haelterman N. A. Sandoval H. Xiong B. Donti T. Kalsotra A. (2015). Impaired mitochondrial energy production causes light-induced photoreceptor degeneration independent of oxidative stress (corrections in 2018). *PLoS Biol.* 13:e1002197. 10.1371/journal.pbio.1002197 26176594 PMC4503542

[B80] Janc O. Müller M. (2014). The free radical scavenger Trolox dampens neuronal hyperexcitability, reinstates synaptic plasticity, and improves hypoxia tolerance in a mouse model of Rett syndrome. *Front. Cell Neurosci.* 8:56. 10.3389/fncel.2014.00056 24605086 PMC3932407

[B81] Ježek J. Cooper K. F. Strich R. (2018). Reactive oxygen species and mitochondrial dynamics: The yin and yang of mitochondrial dysfunction and cancer progression. *Antioxidants* 7:13. 10.3390/antiox7010013 29337889 PMC5789323

[B82] Kan K. T. Nelson M. G. Grant C. M. Hubbard S. J. Lu H. (2022). Understanding the role of yeast Yme1 in mitochondrial function using biochemical and proteomics analyses. *Int. J. Mol. Sci.* 23:13694. 10.3390/ijms232213694 36430179 PMC9694332

[B83] Kan K. T. Wilcock J. Lu H. (2024). Role of Yme1 in mitochondrial protein homeostasis: From regulation of protein import, OXPHOS function to lipid synthesis and mitochondrial dynamics. *Biochem. Soc. Trans.* 52 1539–1548. 10.1042/BST20240450 38864432 PMC11346431

[B84] Karp P. D. Billington R. Caspi R. Fulcher C. A. Latendresse M. Kothari A. (2017). The BioCyc collection of microbial genomes and metabolic pathways. *Brief. Bioinform.* 20 1085–1093. 10.1093/bib/bbx085 29447345 PMC6781571

[B85] Khatun J. Gelles J. D. Chipuk J. E. (2024). Dynamic death decisions: How mitochondrial dynamics shape cellular commitment to apoptosis and ferroptosis. *Dev. Cell* 59 2549–2565. 10.1016/j.devcel.2024.09.004 39378840 PMC11469553

[B86] Kim G. Gautier O. Tassoni-Tsuchida E. Ma X. R. Gitler A. D. (2020). ALS genetics: Gains, losses, and implications for future therapies. *Neuron* 108 822–842. 10.1016/j.neuron.2020.08.022 32931756 PMC7736125

[B87] Kishita Y. Shimura M. Kohda M. Akita M. Imai-Okazaki A. Yatsuka Y. (2020). A novel homozygous variant in MICOS13/QIL1 causes hepato-encephalopathy with mitochondrial DNA depletion syndrome. *Mol. Genet. Genom. Med.* 8:e1427. 10.1002/mgg3.1427 32749073 PMC7549589

[B88] Klionsky D. J. Abdalla F. C. Abeliovich H. Abraham R. T. Acevedo-Arozena A. Adeli K. (2012). Guidelines for the use and interpretation of assays for monitoring autophagy. *Autophagy* 8 445–544. 10.4161/auto.19496 22966490 PMC3404883

[B89] Kuèková K. Thakker A. Vettore L. Escribano-Gonzalez C. Hindshaw R. L. Tearle J. L. E. (2020). Succinate dehydrogenase deficiency in a chromaffin cell model retains metabolic fitness through the maintenance of mitochondrial NADH oxidoreductase function. *FASEB J.* 34 303–315. 10.1096/fj.201901456R 31914648

[B90] Klumpe I. Savvatis K. Westermann D. Tschöpe C. Rauch U. Landmesser U. (2016). Transgenic overexpression of adenine nucleotide translocase 1 protects ischemic hearts against oxidative stress. *J. Mol. Med.* 94 645–653. 10.1007/s00109-016-1413-4 27080394

[B91] Koch J. Broeks M. H. Gautschi M. Jans J. Laemmle A. (2024). Inborn errors of the malate aspartate shuttle – Update on patients and cellular models. *Mol. Genet. Metab.* 142:108520. 10.1016/j.ymgme.2024.108520 38945121

[B92] Kokoszka J. E. Coskun P. Esposito L. A. Wallace D. C. (2001). Increased mitochondrial oxidative stress in the Sod2 (+/-) mouse results in the age-related decline of mitochondrial function culminating in increased apoptosis. *Proc. Natl. Acad. Sci. U. S. A.* 98 2278–2283. 10.1073/pnas.051627098 11226230 PMC30129

[B93] Kokoszka J. E. Waymire K. G. Levy S. E. Sligh J. E. Cai J. Jones D. P. (2004). The ADP/ATP translocator is not essential for the mitochondrial permeability transition pore. *Nature* 427 461–465. 10.1038/nature02229 14749836 PMC3049806

[B94] Kolac U. K. Donmez Yalcin G. Yalcin A. (2023). Chemical inhibition of mitochondrial fission improves insulin signaling and subdues hyperglycemia induced stress in placental trophoblast cells. *Mol. Biol. Rep.* 50 493–506. 10.1007/s11033-022-07959-0 36352179

[B95] Kretova M. Sabova L. Hodny Z. Bartek J. Kollarovic G. Nelson B. D. (2014). TGF-β/NF1/Smad4-mediated suppression of ANT2 contributes to oxidative stress in cellular senescence. *Cell Signal.* 26 2903–2911. 10.1016/j.cellsig.2014.08.029 25220407

[B96] La Rosa P. Petrillo S. Turchi R. Berardinelli F. Schirinzi T. Vasco G. (2021). The Nrf2 induction prevents ferroptosis in Friedreich’s Ataxia. *Redox Biol.* 38:101791. 10.1016/j.redox.2020.101791 33197769 PMC7677700

[B97] Le T. Liang Z. Patel H. Yu M. H. Sivasubramaniam G. Slovitt M. (2006). A new family of *Drosophila* balancer chromosomes with a w- Dfd-GMR yellow fluorescent protein marker. *Genetics* 174 2255–2257. 10.1534/genetics.106.063461 17057238 PMC1698648

[B98] Lee H. Lee T. J. Galloway C. A. Zhi W. Xiao W. de Mesy Bentley K. L. (2023). The mitochondrial fusion protein OPA1 is dispensable in the liver and its absence induces mitohormesis to protect liver from drug-induced injury. *Nat. Commun.* 14:6721. 10.1038/s41467-023-42564-0 37872238 PMC10593833

[B99] Lee J. Schriner S. E. Wallace D. C. (2009). Adenine nucleotide translocator 1 deficiency increases resistance of mouse brain and neurons to excitotoxic insults. *Biochim Biophys. Acta - Bioenerg.* 1787 364–370. 10.1016/j.bbabio.2009.01.014 19366611 PMC3245720

[B100] Lemarie A. Huc L. Pazarentzos E. Mahul-Mellier A. L. Grimm S. (2011). Specific disintegration of complex II succinate:ubiquinone oxidoreductase links pH changes to oxidative stress for apoptosis induction. *Cell Death Diff.* 18 338–349. 10.1038/cdd.2010.93 20706275 PMC3044456

[B101] Li H. Janssens J. De Waegeneer M. Kolluru S. S. Davie K. Gardeux V. (2022). Fly cell atlas: A single-nucleus transcriptomic atlas of the adult fruit fly. *Science* 375:eabk2432. 10.1126/science.abk2432 35239393 PMC8944923

[B102] Li J. Liang N. Long X. Zhao J. Yang J. Du X. (2019). SDHC-related deficiency of SDH complex activity promotes growth and metastasisy promotes growth and metastasis of hepatocellular carcinoma via ROS/NFκB signaling. *Cancer Lett.* 461 44–55. 10.1016/j.canlet.2019.07.001 31278950

[B103] Li Y. Huang T.-T. Carlson E. J. Melov S. Ursell P. C. Olson J. L. (1995). Dilated cardiomyopathy and neonatal lethality in mutant mice lacking manganese superoxide dismutase. *Nat. Genet.* 11 376–381. 10.1038/ng1295-376 7493016

[B104] Liang F. G. Zandkarimi F. Lee J. Axelrod J. L. Pekson R. Yoon Y. (2024). OPA1 promotes ferroptosis by augmenting mitochondrial ROS and suppressing an integrated stress response. *Mol. Cell* 84 3098–3114.e6. 10.1016/j.molcel.2024.07.020. 39142278 PMC11373561

[B105] Liew Y.-T. Voelzmann A. Owens M. Day M. Cairns W. Jones E. (2025). Different mechanisms link gain and loss of kinesin functions to axonal degeneration. *bioRxiv [Preprint]* 10.1101/2024.12.31.630930

[B106] Liu L. MacKenzie K. R. Putluri N. Maletić-Savatić M. Bellen H. J. (2017). The glia-neuron lactate shuttle and elevated ROS promote lipid synthesis in neurons and lipid droplet accumulation in glia via APOE/D. *Cell Metabol.* 26 719–737.e6. 10.1016/j.cmet.2017.08.024. 28965825 PMC5677551

[B107] Llorens J. V. Navarro J. A. Martínez-Sebastián M. J. Baylies M. K. Schneuwly S. Botella J. A. (2007). Causative role of oxidative stress in a *Drosophila* model of Friedreich ataxia. *FASEB J.* 21 333–344. 10.1096/fj.05-5709com 17167074

[B108] Lord S. J. Velle K. B. Mullins R. D. Fritz-Laylin L. K. (2020). SuperPlots: Communicating reproducibility and variability in cell biology. *J. Cell Biol.* 219:e202001064. 10.1083/jcb.202001064 32346721 PMC7265319

[B109] Lovero D. Giordano L. Marsano R. M. Sanchez-Martinez A. Boukhatmi H. Drechsler M. (2018). Characterization of *Drosophila* ATPsynC mutants as a new model of mitochondrial ATP synthase disorders. *PLoS One* 13:e0201811. 10.1371/journal.pone.0201811 30096161 PMC6086398

[B110] Lussey-Lepoutre C. Hollinshead K. E. R. Ludwig C. Menara M. Morin A. Castro-Vega L.-J. (2015). Loss of succinate dehydrogenase activity results in dependency on pyruvate carboxylation for cellular anabolism. *Nat. Commun.* 6:8784. 10.1038/ncomms9784 26522426 PMC4632646

[B111] Magistretti P. J. Allaman I. (2018). Lactate in the brain: From metabolic end-product to signalling molecule. *Nat. Rev. Neurosci.* 19 235–249. 10.1038/nrn.2018.19 29515192

[B112] Marelja Z. Leimkühler S. Missirlis F. (2018). Iron sulfur and molybdenum cofactor enzymes regulate the *Drosophila* life cycle by controlling cell metabolism. *Front. Physiol.* 9:50. 10.3389/fphys.2018.00050 29491838 PMC5817353

[B113] Marlar-Pavey M. Tapias-Gomez D. Mettlen M. Friedman J. R. (2025). Compositionally unique mitochondria in filopodia support cellular migration. *Curr. Biol.* 35 1227–1241.e6. 10.1016/j.cub.2025.01.062. 39978347 PMC11945552

[B114] Marquez M. D. Greth C. Buzuk A. Liu Y. Blinn C. M. Beller S. (2023). Cytosolic iron–sulfur protein assembly system identifies clients by a C-terminal tripeptide. *Proc. Natl. Acad. Sci. U. S. A.* 120:e2311057120. 10.1073/pnas.2311057120 37883440 PMC10623007

[B115] Mast J. D. Tomalty K. M. H. Vogel H. Clandinin T. R. (2008). Reactive oxygen species act remotely to cause synapse loss in a *Drosophila* model of developmental mitochondrial encephalopathy. *Development* 135 2669–2679. 10.1242/dev.020644 18599508 PMC2892278

[B116] Mattedi F. Lloyd-Morris E. Hirth F. Vagnoni A. (2023). Optogenetic cleavage of the Miro GTPase reveals the direct consequences of real-time loss of function in *Drosophila*. *PLoS Biol.* 21:e3002273. 10.1371/journal.pbio.3002273 37590319 PMC10465005

[B117] Melov S. Coskun P. Patel M. Tuinstra R. Cottrell B. Jun A. S. (1999). Mitochondrial disease in superoxide dismutase 2 mutant mice. *Proc. Natl. Acad. Sci. U. S. A.* 96 846–851. 10.1073/pnas.96.3.846 9927656 PMC15313

[B118] Melov S. Schneider J. A. Day B. J. Hinerfeld D. Coskun P. Mirra S. S. (1998). A novel neurological phenotype in mice lacking mitochondrial manganese superoxide dismutase. *Nat. Genet.* 18 159–163. 10.1038/ng0298-159 9462746

[B119] Millet A. M. C. Bertholet A. M. Daloyau M. Reynier P. Galinier A. Devin A. (2016). Loss of functional OPA1 unbalances redox state: Implications in dominant optic atrophy pathogenesis. *Ann. Clin. Transl. Neurol.* 3 408–421. 10.1002/acn3.305 27547769 PMC4891995

[B120] Misgeld T. Schwarz T. L. (2017). Mitostasis in neurons: Maintaining mitochondria in an extended cellular architecture. *Neuron* 96 651–666. 10.1016/j.neuron.2017.09.055 29096078 PMC5687842

[B121] Monfort B. Want K. Gervason S. D’Autréaux B. (2022). Recent advances in the elucidation of Frataxin biochemical function: Open novel perspectives for the treatment of Friedreich’s ataxia. *Front. Neurosci.* 16:838335. 10.3389/fnins.2022.838335 35310092 PMC8924461

[B122] Mukherjee I. Ghosh M. Meinecke M. (2021). MICOS and the mitochondrial inner membrane morphology – when things get out of shape. *FEBS Lett.* 595 1159–1183. 10.1002/1873-3468.14089 33837538

[B123] Murphy M. P. (2008). How mitochondria produce reactive oxygen species. *Biochem. J.* 417 1–13. 10.1042/BJ20081386 19061483 PMC2605959

[B124] Naifeh J. Dimri M. Varacallo M. (2024). “Biochemistry, aerobic glycolysis,” in *StatPearls*, (Treasure Island, FL: StatPearls Publishing). Available online at: https://www.ncbi.nlm.nih.gov/books/NBK470170 29262043

[B125] Navarro J. A. Botella J. A. Metzendorf C. Lind M. I. Schneuwly S. (2015). Mitoferrin modulates iron toxicity in a *Drosophila* model of Friedreich’s ataxia. *Free Rad. Biol. Med.* 85 71–82. 10.1016/j.freeradbiomed.2015.03.014 25841783

[B126] Neilson D. E. Zech M. Hufnagel R. B. Slone J. Wang X. Homan S. (2022). A novel variant of ATP5MC3 associated with both dystonia and spastic paraplegia. *Mov. Disord.* 37 375–383. 10.1002/mds.28821 34636445 PMC8840961

[B127] Nemeria N. S. Zhang X. Leandro J. Zhou J. Yang L. Houten S. M. (2021). Toward an understanding of the structural and mechanistic aspects of protein-protein interactions in 2-oxoacid dehydrogenase complexes. *Life* 11:407. 10.3390/life11050407 33946784 PMC8146983

[B128] Nulton-Persson A. C. Starke D. W. Mieyal J. J. Szweda L. I. (2003). Reversible inactivation of α-ketoglutarate dehydrogenase in response to alterations in the mitochondrial glutathione status. *Biochemistry* 42 4235–4242. 10.1021/bi027370f 12680778

[B129] Nulton-Persson A. C. Szweda L. I. (2001). Modulation of mitochondrial function by hydrogen peroxide. *J. Biol. Chem.* 276 23357–23361. 10.1074/jbc.M100320200 11283020

[B130] O’Hanlon M. E. Tweedy C. Scialo F. Bass R. Sanz A. Smulders-Srinivasan T. K. (2022). Mitochondrial electron transport chain defects modify Parkinson’s disease phenotypes in a *Drosophila* model. *Neurobiol. Dis.* 171:105803. 10.1016/j.nbd.2022.105803 35764292

[B131] Oh S. S. Sullivan K. A. Wilkinson J. E. Backus C. Hayes J. M. Sakowski S. A. (2012). Neurodegeneration and early lethality in superoxide dismutase 2-deficient mice: A comprehensive analysis of the central and peripheral nervous systems. *Neuroscience* 212 201–213. 10.1016/j.neuroscience.2012.03.026 22516022 PMC3367053

[B132] Okenve-Ramos P. Gosling R. Chojnowska-Monga M. Gupta K. Shields S. Alhadyian H. (2024). Neuronal ageing is promoted by the decay of the microtubule cytoskeleton. *PLoS Biol.* 22:e3002504. 10.1371/journal.pbio.3002504 38478582 PMC10962844

[B133] Owens K. M. Aykin-Burns N. Dayal D. Coleman M. C. Domann F. E. Spitz D. R. (2012). Genomic instability induced by mutant succinate dehydrogenase subunit D (SDHD) is mediated by O2- and H2O2. *Free Rad. Biol. Med.* 52 160–166. 10.1016/j.freeradbiomed.2011.10.435 22041456 PMC3249516

[B134] Palma F. R. He C. Danes J. M. Paviani V. Coelho D. R. Gantner B. N. (2020). Mitochondrial superoxide dismutase: What the established, the intriguing, and the novel reveal about a key cellular redox switch. *Antioxid Redox Signal* 32 701–714. 10.1089/ars.2019.7962 31968997 PMC7047081

[B135] Papanicolaou K. N. Ngoh G. A. Dabkowski E. R. O’Connell K. A. Ribeiro R. F. Stanley W. C. (2012). Cardiomyocyte deletion of mitofusin-1 leads to mitochondrial fragmentation and improves tolerance to ROS-induced mitochondrial dysfunction and cell death. *Am. J. Physiol. Heart Circ. Physiol.* 302 H167–H179. 10.1152/ajpheart.00833.2011 22037195 PMC3334239

[B136] Parks A. L. Cook K. R. Belvin M. Dompe N. A. Fawcett R. Huppert K. (2004). Systematic generation of high-resolution deletion coverage of the *Drosophila melanogaster* genome. *Nat. Genet.* 36 288–292. 10.1038/ng1312 14981519

[B137] Patel K. P. O’Brien T. W. Subramony S. H. Shuster J. Stacpoole P. W. (2012). The spectrum of pyruvate dehydrogenase complex deficiency: Clinical, biochemical and genetic features in 371 patients. *Mol. Genet. Metab.* 106 385–394. 10.1016/j.ymgme.2012.03.017 22896851 PMC4003492

[B138] Patel M. S. Nemeria N. S. Furey W. Jordan F. (2014). The pyruvate dehydrogenase complexes: Structure-based function and regulation. *J. Biol. Chem.* 289 16615–16623. 10.1074/jbc.R114.563148 24798336 PMC4059105

[B139] Patergnani S. Morciano G. Carinci M. Leo S. Pinton P. Rimessi A. (2022). The “mitochondrial stress responses”: The “Dr. Jekyll and Mr. Hyde” of neuronal disorders. *Neural Regenerat. Res.* 17 2563–2575. 10.4103/1673-5374.339473 35662183 PMC9165365

[B140] Paul A. Belton A. Nag S. Martin I. Grotewiel M. S. Duttaroy A. (2007). Reduced mitochondrial SOD displays mortality characteristics reminiscent of natural aging. *Mech. Ageing Dev.* 128 706–716. 10.1016/j.mad.2007.10.013 18078670 PMC2675272

[B141] Perkins L. A. Holderbaum L. Tao R. Hu Y. Sopko R. McCall K. (2015). The transgenic RNAi project at harvard medical school: Resources and validation. *Genetics* 201 843–852. 10.1534/genetics.115.180208 26320097 PMC4649654

[B142] Pfanner N. Warscheid B. Wiedemann N. (2019). Mitochondrial proteins: From biogenesis to functional networks. *Nat. Rev. Mol. Cell Biol.* 20 267–284. 10.1038/s41580-018-0092-0 30626975 PMC6684368

[B143] Piazza N. Hayes M. Martin I. Duttaroy A. Grotewiel M. Wessells R. (2009). Multiple measures of functionality exhibit progressive decline in a parallel, stochastic fashion in *Drosophila* Sod2 null mutants. *Biogerontology* 10 637–648. 10.1007/s10522-008-9210-2 19148770 PMC2800787

[B144] Picard M. McManus M. J. Csordás G. Várnai P. Dorn, Ii G. W. (2015). Trans-mitochondrial coordination of cristae at regulated membrane junctions. *Nat. Commun.* 6:6259. 10.1038/ncomms7259 25687472 PMC4332397

[B145] Picca A. Guerra F. Calvani R. Coelho-Júnior H. J. Landi F. Bucci C. (2023). Mitochondrial-derived vesicles: The good, the bad, and the ugly. *Int. J. Mol. Sci.* 24:13835. 10.3390/ijms241813835 37762138 PMC10531235

[B146] Prokop A. (2013). A rough guide to *Drosophila* mating schemes. *Figshare* 6, 1–39. 10.6084/m9.figshare.106631

[B147] Prokop A. (2021). A common theme for axonopathies? The dependency cycle of local axon homeostasis. *Cytoskeleton* 78 52–63. 10.1002/cm.21657 33713552

[B148] Prokop A. Beaven R. Qu Y. Sánchez-Soriano N. (2013). Using fly genetics to dissect the cytoskeletal machinery of neurons during axonal growth and maintenance. *J. Cell Sci.* 126 2331–2341. 10.1242/jcs.126912 23729743

[B149] Prokop A. Küppers-Munther B. Sánchez-Soriano N. (2012). Using primary neuron cultures of *Drosophila* to analyse neuronal circuit formation and function. *Mak. Un-mak. Neuronal Circuits Drosophila* 69 225–247. 10.1007/978-1-61779-830-6_10

[B150] Qi X. Lewin A. S. Hauswirth W. W. Guy J. (2003). Optic neuropathy induced by reductions in mitochondrial superoxide dismutase. *Invest Ophthalmol. Vis. Sci.* 44 1088–1096. 10.1167/iovs.02-0864 12601034

[B151] Qi Y. Liu H. Daniels M. P. Zhang G. Xu H. (2016). Loss of *Drosophila* i-AAA protease, dYME1L, causes abnormal mitochondria and apoptotic degeneration. *Cell Death Different.* 23 291–302. 10.1038/cdd.2015.94 26160069 PMC4716308

[B152] Qu Y. Alves-Silva J. Gupta K. Hahn I. Parkin J. Sánchez-Soriano N. (2022). Re-evaluating the actin-dependence of spectraplakin functions during axon growth and maintenance. *Dev. Neurobiol.* 82 288–307. 10.1002/dneu.22873 35333003 PMC9320987

[B153] Qu Y. Hahn I. Webb S. E. D. Pearce S. P. Prokop A. (2017). Periodic actin structures in neuronal axons are required to maintain microtubules. *Mol. Biol. Cell* 28 296–308. 10.1091/mbc.e16-10-0727 27881663 PMC5231898

[B154] Quintana-Cabrera R. Scorrano L. (2023). Determinants and outcomes of mitochondrial dynamics. *Mol. Cell* 83 857–876. 10.1016/j.molcel.2023.02.012 36889315

[B155] Rana A. Oliveira M. P. Khamoui A. V. Aparicio R. Rera M. Rossiter H. B. (2017). Promoting Drp1-mediated mitochondrial fission in midlife prolongs healthy lifespan of *Drosophila melanogaster*. *Nat. Commun.* 8:448. 10.1038/s41467-017-00525-4 28878259 PMC5587646

[B156] Rossi A. Pizzo P. Filadi R. (2019). Calcium, mitochondria and cell metabolism: A functional triangle in bioenergetics. *Biochim. Biophys. Acta Mol. Cell Res.* 1866 1068–1078. 10.1016/j.bbamcr.2018.10.016 30982525

[B157] Russell B. E. Whaley K. G. Bove K. E. Labilloy A. Lombardo R. C. Hopkin R. J. (2019). Expanding and underscoring the hepato−encephalopathic phenotype of QIL1/MIC13. *Hepatology* 70 1066–1070. 10.1002/hep.30627 30912852 PMC11108097

[B158] Rutter J. Winge D. R. Schiffman J. D. (2010). Succinate dehydrogenase – assembly, regulation and role in human disease. *Mitochondrion* 10 393–401. 10.1016/j.mito.2010.03.001 20226277 PMC2874626

[B159] Ryder E. Ashburner M. Bautista-Llacer R. Drummond J. Webster J. Johnson G. (2007). The DrosDel deletion collection: A *Drosophila* genomewide chromosomal deficiency resource. *Genetics* 177 615–629. 10.1534/genetics.107.076216 17720900 PMC2013729

[B160] Sánchez-Soriano N. Gonçalves-Pimentel C. Beaven R. Haessler U. Ofner L. Ballestrem C. (2010). *Drosophila* growth cones: A genetically tractable platform for the analysis of axonal growth dynamics. *Dev. Neurobiol.* 70 58–71. 10.1002/dneu.20762 19937774

[B161] Sandoval H. Yao C.-K. Chen K. Jaiswal M. Donti T. Lin Y. Q. (2014). Mitochondrial fusion but not fission regulates larval growth and synaptic development through steroid hormone production. *eLife* 3:e03558. 10.7554/eLife.03558 25313867 PMC4215535

[B162] Schneider I. (1964). Differentiation of larval *Drosophila* eye-antennal discs in vitro. *J. Exp. Zool.* 156 91–104. 10.1002/jez.1401560107 14189923

[B163] Shi R. Hou W. Wang Z.-Q. Xu X. (2021). Biogenesis of iron–sulfur clusters and their role in DNA metabolism. *Front. Cell Dev. Biol.* 9:735678. 10.3389/fcell.2021.735678 34660592 PMC8514734

[B164] Shidara Y. Hollenbeck P. J. (2010). Defects in mitochondrial axonal transport and membrane potential without increased reactive oxygen species production in a *Drosophila* model of Friedreich ataxia. *J. Neurosci.* 30 11369–11378. 10.1523/jneurosci.0529-10.2010 20739558 PMC2943153

[B165] Shields S. Gregory E. Wilkes O. Gozes I. Sánchez-Soriano N. (2025). Oxidative stress promotes axonal atrophy through alterations in microtubules and EB1 function. *Aging Dis.* 10.14336/AD.2024.0839 [Epub ahead of print]. 39908272 PMC12539546

[B166] Simon D. Seznec H. Gansmuller A. Carelle N. Weber P. Metzger D. (2004). Friedreich ataxia mouse models with progressive cerebellar and sensory ataxia reveal autophagic neurodegeneration in dorsal root ganglia. *J. Neurosci.* 24 1987–1995. 10.1523/jneurosci.4549-03.2004 14985441 PMC6730414

[B167] Smith G. Sweeney S. O’Kane C. J. Prokop A. (2023). How neurons maintain their axons long-term: An integrated view of axon biology and pathology. *Front. Neurosci.* 17:1236815. 10.3389/fnins.2023.1236815 37564364 PMC10410161

[B168] Spradling A. C. Stern D. Beaton A. Rhem E. J. Laverty T. Mozden N. (1999). The berkeley *Drosophila* genome project gene disruption project. single P-element insertions mutating 25% of vital *Drosophila genes*. *Genetics* 153 135–177. 10.1093/genetics/153.1.135 10471706 PMC1460730

[B169] Sprenger H. G. Wani G. Hesseling A. König T. Patron M. MacVicar T. (2019). Loss of the mitochondrial i-AAA protease YME1L leads to ocular dysfunction and spinal axonopathy. *EMBO Mol. Med.* 11:e9288. 10.15252/emmm.201809288 30389680 PMC6328943

[B170] Sturm G. Hake K. Lefebvre A. E. Y. T. Rux C. J. Ivanova D. Millett-Sikking A. (2024). The biophysical mechanism of mitochondrial pearling. *bioRxiv [Preprint]* 10.1101/2024.12.21.629509 PMC12586885 41060791

[B171] Tang S. Le P. K. Tse S. Wallace D. C. Huang T. (2009). Heterozygous mutation of Opa1 in *Drosophila* shortens lifespan mediated through increased reactive oxygen species production. *PLoS One* 4:e4492. 10.1371/journal.pone.0004492 19221591 PMC2637430

[B172] Tapia A. Palomino-Schätzlein M. Roca M. Lahoz A. Pineda-Lucena A. López (2021). Mild muscle mitochondrial fusion distress extends *Drosophila* lifespan through an early and systemic metabolome reorganization. *Int. J. Mol. Sci.* 22:12133. 10.3390/ijms222212133 34830014 PMC8618903

[B173] Terhzaz S. Cabrero P. Chintapalli V. R. Davies S.-A. Dow J. A. T. (2010). Mislocalization of mitochondria and compromised renal function and oxidative stress resistance in *Drosophila* SesB mutants. *Physiol. Genom.* 41 33–41. 10.1152/physiolgenomics.00147.2009 20009008 PMC2841493

[B174] Tsakiri E. N. Gumeni S. Iliaki K. K. Benaki D. Vougas K. Sykiotis G. P. (2019a). Hyperactivation of Nrf2 increases stress tolerance at the cost of aging acceleration due to metabolic deregulation. *Aging Cell* 18:e12845. 10.1111/acel.12845 30537423 PMC6351879

[B175] Tsakiri E. N. Gumeni S. Vougas K. Pendin D. Papassideri I. Daga A. (2019b). Proteasome dysfunction induces excessive proteome instability and loss of mitostasis that can be mitigated by enhancing mitochondrial fusion or autophagy. *Autophagy* 15 1757–1773. 10.1080/15548627.2019.1596477 31002009 PMC6735541

[B176] Tur J. Pereira-Lopes S. Vico T. Marín E. A. Muoz J. P. Hernández-Alvarez M. (2020). Mitofusin 2 in macrophages links mitochondrial ROS production, cytokine release, phagocytosis, autophagy, and bactericidal activity. *Cell Rep.* 32:108079. 10.1016/j.celrep.2020.108079 32846136

[B177] Uo T. Dworzak J. Kinoshita C. Inman D. M. Kinoshita Y. Horner P. J. (2009). Drp1 levels constitutively regulate mitochondrial dynamics and cell survival in cortical neurons. *Exp. Neurol.* 218 274–285. 10.1016/j.expneurol.2009.05.010 19445933 PMC2733949

[B178] Vallières C. Benoit O. Guittet O. Huang M.-E. Lepoivre M. Golinelli-Cohen M.-P. (2024). Iron-sulfur protein odyssey: Exploring their cluster functional versatility and challenging identification. *Metallomics* 16:mfae025. 10.1093/mtomcs/mfae025 38744662 PMC11138216

[B179] Van Vranken J. G. Bricker, Daniel K. Dephoure N. Gygi, Steven P. (2014). SDHAF4 promotes mitochondrial succinate dehydrogenase activity and prevents neurodegeneration. *Cell Metab.* 20 241–252. 10.1016/j.cmet.2014.05.012 24954416 PMC4126880

[B180] Vartiainen S. Chen S. George J. Tuomela T. Luoto K. R. O’Dell K. M. C. (2014). Phenotypic rescue of a *Drosophila* model of mitochondrial ANT1 disease. *Dis. Models Mech.* 7 635–648. 10.1242/dmm.016527 24812436 PMC4036471

[B181] Verstreken P. Ly C. V. Venken K. J. Koh T. W. Zhou Y. Bellen H. J. (2005). Synaptic mitochondria are critical for mobilization of reserve pool vesicles at *Drosophila* neuromuscular junctions. *Neuron* 47 365–378. 10.1016/j.neuron.2005.06.018 16055061

[B182] Voeltz G. K. Sawyer E. M. Hajnóczky G. Prinz W. A. (2024). Making the connection: How membrane contact sites have changed our view of organelle biology. *Cell* 187 257–270. 10.1016/j.cell.2023.11.040 38242082 PMC11830234

[B183] Voelzmann A. Nuhu-Soso L. Roof A. Patel S. Bennett H. Adamson A. (2024). Mis-regulation of GSK-3β causes axonal microtubule curling through Shot and Tau. *bioRxiv [Preprint]* 10.1101/2024.09.08.611864 40654684 PMC12247919

[B184] Voelzmann A. Sánchez-Soriano N. (2022). “*Drosophila* primary neuronal cultures as a useful cellular model to study and image axonal transport (Chapter 23),” in *Axonal transport. methods in molecular biology*, ed. Vagnoni A. (New York, NY: Humana), 10.1007/978-1-0716-1990-2_23 35412291

[B185] Vrailas-Mortimer A. del Rivero T. Mukherjee S. Nag S. Gaitanidis A. Kadas D. (2011). A muscle-specific p38 MAPK/Mef2/MnSOD pathway regulates stress, motor function, and life span in *Drosophila*. *Dev. Cell* 21 783–795. 10.1016/j.devcel.2011.09.002 22014527 PMC3199449

[B186] Wai T. García-Prieto J. Baker M. J. Merkwirth C. Benit P. Rustin P. (2015). Imbalanced OPA1 processing and mitochondrial fragmentation cause heart failure in mice. *Science* 350:aad0116. 10.1126/science.aad0116 26785494

[B187] Walters G. C. Usachev Y. M. (2023). Mitochondrial calcium cycling in neuronal function and neurodegeneration. *Front. Cell Dev. Biol.* 11:1094356. 10.3389/fcell.2023.1094356 36760367 PMC9902777

[B188] Waltz F. Righetto R. D. Lamm L. Salinas-Giegé T. Kelley R. Zhang X. (2024). In-cell architecture of the mitochondrial respiratory chain. *Science* 387 1296–1301. 10.1126/science.ads8738 40112058

[B189] Wang B. Huang M. Shang D. Yan X. Zhao B. Zhang X. (2021). Mitochondrial behavior in axon degeneration and regeneration. *Front. Aging Neurosci.* 13:650038. 10.3389/fnagi.2021.650038 33762926 PMC7982458

[B190] Wang H. Lu J. Kulkarni S. Zhang W. Gorka J. E. Mandel J. A. (2019). Metabolic and oncogenic adaptations to pyruvate dehydrogenase inactivation in fibroblasts. *J. Biol. Chem.* 294 5466–5486. 10.1074/jbc.RA118.005200 30755479 PMC6462518

[B191] Wang L.-J. Hsu T. Lin H.-L. Fu C.-Y. (2020). *Drosophila* MICOS knockdown impairs mitochondrial structure and function and promotes mitophagy in muscle tissue. *Biol Open* 9:bio054262. 10.1242/bio.054262 33268479 PMC7725604

[B192] Wang M.-Y. Zhou Y. Li W.-L. Zhu L.-Q. Liu D. (2024). Friend or foe: Lactate in neurodegenerative diseases. *Ageing Res. Rev.* 101:102452. 10.1016/j.arr.2024.102452 39127445

[B193] Wicks S. Bain N. Duttaroy A. Hilliker A. J. Phillips J. P. (2009). Hypoxia rescues early mortality conferred by superoxide dismutase deficiency. *Free Rad. Biol. Med.* 46 176–181. 10.1016/j.freeradbiomed.2008.09.036 18983909

[B194] Williams C. T. De Jesus O. (2024). “Friedreich ataxia,” in *StatPearls*, (Treasure Island, FL: StatPearls Publishing). Available online at: https://www.ncbi.nlm.nih.gov/books/NBK563199/

[B195] Yamamoto S. Jaiswal M. Charng W. L. Gambin T. Karaca E. Mirzaa G. (2014). A *Drosophila* genetic resource of mutants to study mechanisms underlying human genetic diseases. *Cell* 159 200–214. 10.1016/j.cell.2014.09.002 25259927 PMC4298142

[B196] Yang C. Pan R.-Y. Guan F. Yuan Z. (2024). Lactate metabolism in neurodegenerative diseases. *Neur. Regen. Res.* 19 69–74. 10.4103/1673-5374.374142 37488846 PMC10479854

[B197] Yap Z. Y. Efthymiou S. Seiffert S. Vargas Parra K. Lee S. Nasca A. (2021a). Bi-allelic variants in OGDHL cause a neurodevelopmental spectrum disease featuring epilepsy, hearing loss, visual impairment, and ataxia. *Am. J. Hum. Genet.* 108 2368–2384. 10.1016/j.ajhg.2021.11.003 34800363 PMC8715183

[B198] Yap Z. Y. Strucinska K. Matsuzaki S. Lee S. Si Y. Humphries K. (2021b). A biallelic pathogenic variant in the gene results in a neurological disorder with features of a mitochondrial disease. *J. Inherit. Metab. Dis.* 44 388–400. 10.1002/jimd.12248 32383294 PMC7647956

[B199] Yarosh W. Monserrate J. Tong J. J. Tse S. Le P. K. Nguyen K. (2008). The molecular mechanisms of OPA1-Mediated optic atrophy in *Drosophila* model and prospects for antioxidant treatment. *PLoS Genet.* 4:e6. 10.1371/journal.pgen.0040006 18193945 PMC2174975

[B200] Yoon W. H. Sandoval H. Nagarkar-Jaiswal S. Jaiswal M. Yamamoto S. Haelterman N. A. (2017). Loss of nardilysin, a mitochondrial co-chaperone for α-ketoglutarate dehydrogenase, promotes mTORC1 activation and neurodegeneration. *Neuron* 93 115–131. 10.1016/j.neuron.2016.11.038 28017472 PMC5242142

[B201] Yu W. Sun Y. Guo S. Lu B. (2011). The PINK1/Parkin pathway regulates mitochondrial dynamics and function in mammalian hippocampal and dopaminergic neurons. *Hum. Mol. Genet.* 20 3227–3240. 10.1093/hmg/ddr235 21613270 PMC3140825

[B202] Zeharia A. Friedman J. R. Tobar A. Saada A. Konen O. Fellig Y. (2016). Mitochondrial hepato-encephalopathy due to deficiency of QIL1/MIC13 (C19orf70), a MICOS complex subunit. *Eur. J. Hum. Genet.* 24 1778–1782. 10.1038/ejhg.2016.83 27485409 PMC5117932

[B203] Zhang J. Liu X. Liang X. Lu Y. Zhu L. Fu R. (2017). A novel ADOA-associated OPA1 mutation alters the mitochondrial function, membrane potential, ROS production and apoptosis. *Sci. Rep.* 7:5704. 10.1038/s41598-017-05571-y 28720802 PMC5515948

[B204] Zhou A. Rohou A. Schep D. G. Bason J. V. Montgomery M. G. Walker J. E. (2015). Structure and conformational states of the bovine mitochondrial ATP synthase by cryo-EM. *eLife* 4:e10180. 10.7554/eLife.10180 26439008 PMC4718723

[B205] Zhu J.-Y. Duan J. van de Leemput J. Han Z. (2024). Dysfunction of mitochondrial dynamics induces endocytosis defect and cell damage in *Drosophila* nephrocytes. *Cells* 13:1253. 10.3390/cells13151253 39120284 PMC11312102

[B206] Zorov D. B. Juhaszova M. Sollott S. J. (2014). Mitochondrial reactive oxygen species (ROS) and ROS-induced ROS release. *Phys. Rev.* 94 909–950. 10.1152/physrev.00026.2013 24987008 PMC4101632

