# Supplementary material for: Impacts of mitochondrial dysfunction on axonal microtubule bundles as a potential mechanism of neurodegeneration
Source: Front Neurosci. 2025 Aug 19;19:1631752. doi: 10.3389/fnins.2025.1631752 (PMC12402001; doi:10.3389/fnins.2025.1631752)
Supplement: Supplementary file 1 [file Data_Sheet_1.zip › SupplTable1.pdf]

gene	human orthologue	function	manipulation	MDI	neurological symptoms (OMIM ref. no.)	*in vivo* data in fly	effect on ETC or ROS
Pdha1	PDHA1	catalytic component of PDC (pyruvate + CoA-SH → acetyl-CoA)	**KO** (*A*); **IR** (*HMC04032*)	5 DIV ↑ **** (1.73-1.82) Trolox rescue	neurological dysfunction (lactic acidosis/Leigh disease; XD#312170)	↑CoA levels (*HMC04032*); ↓photoreceptors	mouse/human: increased ROS in cultured cells
Ogdh1	OGDH	Krebs cycle: α-ketoglutarate → succinyl-CoA	**KO** (*MI06026-TG4.1*), **KO/Df**	5 DIV → (0.91-1.01)	movement disorders, ataxia, seizure (AR#203740)	embryonic lethal (*MI06026-TG4.1*); KD in photoreceptors: gradual loss of synaptic transmission	loss of OGDH: reduced O_2 consumption
SdhA	SDHA	Krebs cycle: succinate → fumarate; ETC: complex II component	**KO** (*1404*, *1110*)	5 DIV ↑ **** (1.83-2.13) Trolox rescue	ND with ataxia & OA (AD#619259)	gradual synapse loss (*1110* &*1404*); enhances ND phenotypes of *park* and *Sirup*/SDHAF4	SDH complex deficiency causes ROS; synapse loss in fly is ROS-mediated; fly SdhA is upregulated by Cnc/Nrf2
AtpSynC	ATP5MC1/2/3	F_0 components of ATP synthase; potential mPTP components	**KO** (*KG01914*); **KO/Df**	1/3/5 DIV ↓→ (1.03-1.32)	ND: dystonia/SP (AD#619681)	larval lethal (*KG01914*)	mouse: loss of OXPHOS, protective against cellular stressors; fly: loss of cristae (*KG01914*); loss of OXPHOS
mt:ATPase6	MT-ATP6		**KO** (*1*)	5 DIV → (1.04)	severe neurological conditions (e.g. ataxia)	young flies: rounded cristae, ↓locomotion, seizure & lethality (all *1*)	humans: ROS overproduction (mPTP-related?); fly: loss of OXPHOS, glycolysis compensates (*1*)
QIL1	MICOS13	key component required for MICOS complex formation	**IR** (*GLC01383*)	5/6 DIV → (1.11)	ND with severe atrophies (#618329)	↓QIL expression (>25%), aberrant mitochondria, ↑mitophagy, no cell death (all *GLC01383*)	reduced O_2 consumption; no reported ROS increase
Opa1	OPA1	fusion of inner mito membrane; cristae formation	**KO** (*s3475*), **IR** (*HMS00349*)	5 DIV/pre → (0.94-1.30)	NDs: Behr syndrome (AR#210000); OA (AD#165500: AD#125250)	↓lifespan, but also rescue of tau-mediated ND (all *s3475*); ↑lifespan, ↑locomotion (*HMS00349*)	mouse: ↑ROS & ↑mtDNA damage, but also ↓ROS; fly: ↑ROS-mediated necrosis in eye (*s3475*); but also beneficial
Marf	MFN1/2	fusion of outer mito membrane	**KO** (*B*), **IR** (*HMC03883*)	5 DIV/pre → (0.60-1.05)	NDs: 2 CMTs (AD#609260; AR#617087); 2 NPs (AD#601152; AR#151800)	enhanced decline in HSP models, ↑ROS, ↑ER stress, ↑axon degeneration (*HMC03883*); but also improving frataxin,	human: ↑ROS; mouse: improved ROS tolerance, ↓ROS; fly: ↑ROS in nephrocytes (*HMC03883*), but also ameliorating

						Huntington's & PD models, ↑lifespan, ↑locomotion	phenotypes in disease models
Drp1	DNM1L	mitochondrial fission	**KO** (*T26*)	5 DIV/pre → (1.03-1.32)	NDs: EncP (AD/R#614388); OA (AD#610708)	↓lifespan; but also ↑lifespan in proteasome-deficient model; rescue of ND in ALS model	human/mouse: ↓ROS
YME1L	YME1L1	i-AAA protease (IMM)	**KO** (*del*)	5 DIV ↓** (0.53)	ND: OA (AR#617302); late onset of ND in conditional mouse models	adults: mitos with aberrant cristae & aggregates, ↓locomotion, precocious death (all *del*)	fly: increased ROS at late stages; yeast: reduced OXPHOS; human: increased lactate/pyruvate levels
sesB	SLC25A4/5/6/31 aka ANT1-4	ADP/ATP antiporter (IMM)	**IR1** (*HMS01549*), **IR2** (*JF01528*)	4/5 DIV ↑ **** (2.54-3.36) Trolox rescue	loose links to ND in humans; mouse myoblasts: ↓ETC dysfunction, ↓mito Ca^{2+} response	↓ATP, ↓mito Ca^{2+} response, ↑autophagy, ↑ND	mouse: ↓glutathione, ↓mito peroxidases; fly: ↑H_2O_2
Sod2	SOD2	Mn superoxide dismutase (superoxide → H_2O_2)	**KO** (*n283*)	3 DIV ↑ **** (2.78) Trolox rescue	loose links to ND in humans; early death & severe ND in mice with CNS-specific SOD2 loss	↓life span, ↑ND/brain apoptosis, ↓locomotion (all *n283*); precocious axon decay, MT curling	fly: life span rescued by hypoxia; mouse: oxidative stress, proton leakage, DNA damage
fh	FXN	iron-sulfur-cluster formation	**KO** (*1*); **IR** (*RNAi.A2*)	3/5/6 DIV ↑ **** (2.90-2.93) Trolox rescue	Friedreich's ataxia (AR; 229300)	↓photoreceptors, ↓ETC, ↓ATP, ↑Fe^{2+} (all *1*); ↓development/life span, ↓ETC (all *RNAi.A2*)	fly: ROS reports contradictory; mouse: ↓ISC, ↑iron, ↓Sod1/2, ↑Fenton react.

Suppl. Tab.1 Summary of findings reported here and by others about the employed genes, their functions, genetic tools and phenotypes. Column 1: the names of studied *Drosophila* genes; column2: their human orthologues; column 3: a crude description of the respective gene's functions; column 4: the genetic manipulations employed (see abbreviations below; names of allele and knock-down lines provided in brackets); column 5: summary of information from Fig.ZZ2 including the length of culture (DIV), the MDI trend (↓ decreased; ↑ increased; → no change), significance (asterisks) and value range from different LOF conditions (in brackets), as well as potential Trolox rescue as reported in Fig.ZZ5; column 6: summary of gene-linked neurological conditions or findings in mammalian models as described and referenced in the main text; column 7: summary of *in vivo* findings in *Drosophila* as described and referenced in the main text (findings obtained with the same tools as listed in column 4 are indicated by brackets); column 8: information relating to ROS upon gene manipulations in fly, mammalian models or humans as described and referenced in the Discussion. Abbreviations: ; AD/R, autosomal dominant/recessive; CMT, Charcot Marie Tooth disease; DIV, days *in vitro*; EncP, encephalopathy; i-AAA proteases, ATPase associated with various cellular activities exposed to the inter-membrane space; IMM, inner mitochondrial membrane; ISC, iron-sulfur cluster; KD, knock-down (elav-Gal4-driven siRNA expression); KO, knock-out (complete or severe loss of function); ND, neurodegenerative disorder; NP, neuropathy; OA, optic atrophy; PD, Parkinson's disease; PDC, pyruvate dehydrogenase complex; vs., versus; XD, X-chromosomal-linked dominant.
