# Supplementary material for: Impacts of mitochondrial dysfunction on axonal microtubule bundles as a potential mechanism of neurodegeneration
Source: Front Neurosci. 2025 Aug 19;19:1631752. doi: 10.3389/fnins.2025.1631752 (PMC12402001; doi:10.3389/fnins.2025.1631752)
Supplement: Supplementary file 1 [file Data_Sheet_1.zip › SupplMat1.pdf]

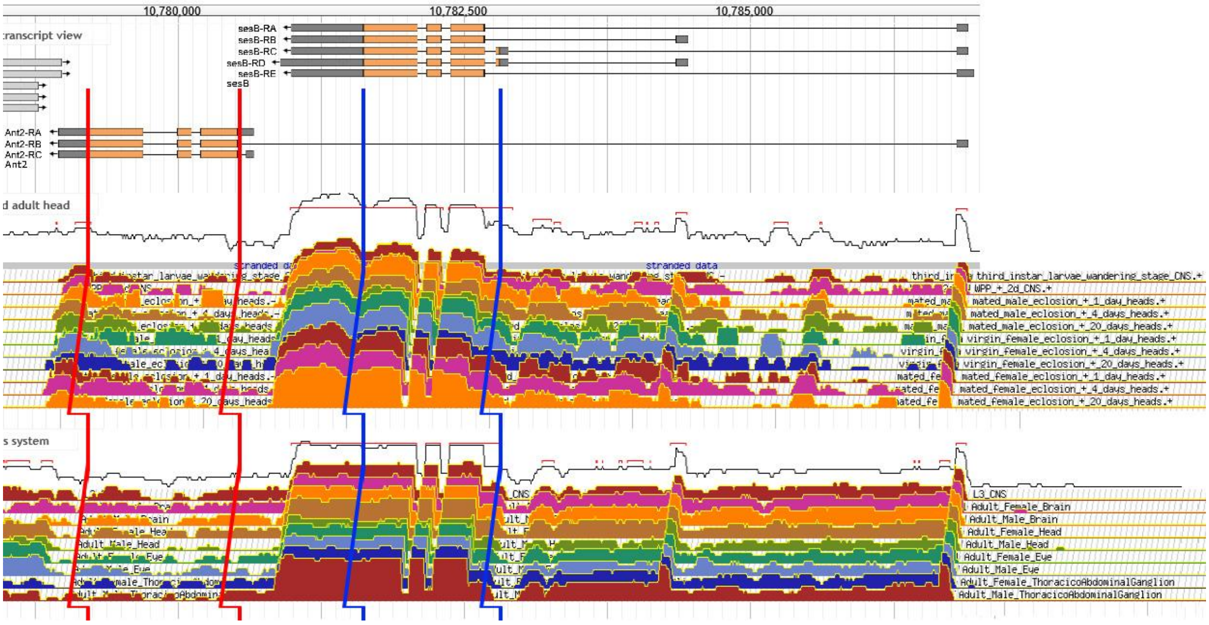

Suppl. Mat. 1. Expression profiles of SesB and Ant2 in the *Drosophila* nervous system. Data are taken from flybase.org (FBgn0003360 and FBgn0025111). The top shows the 5 different transcripts of *sesB* with coding sequence shown in orange, and the 3 RNA isoforms of Ant2. Images below show the expression profiles as determined by RNAseq, with a group of CNS- and adult head-specific modENCODE transcriptomes shown in the middle and a group of nervous system-specific FlyAtlas2 transcriptomes at the bottom. Red lines demarcate the coding region of *Ant2* and blue lines of *sesB*.
