# Supplementary material for: Impacts of mitochondrial dysfunction on axonal microtubule bundles as a potential mechanism of neurodegeneration
Source: Front Neurosci. 2025 Aug 19;19:1631752. doi: 10.3389/fnins.2025.1631752 (PMC12402001; doi:10.3389/fnins.2025.1631752)
Supplement: Supplementary file 1 [file Data_Sheet_1.zip › SupplMat2.pdf]

> ATPsynC (FBtr0085773, ATPsynC-RC, 1794nt, 138aa)

MFVSTVSRIAPVARSAFLANSKQYLRPLSSAIISQSQTLAAQNTTPVALLPQIRSFQTSPVTRDIDSA
AKFIGAGAATVGVAGSGAGIGTVFGSLIIGYARNPSLKQQLFSYAILGFALSEAMGLFCLMMAFLLLF
AF

> ATP5MC2 (ENST00000394349.9, ATP5MC2-202, 632nt, 141aa)

MFACSKFVSTPSLVKSTSQLLSRPLSAVVLKRPEILTDESLSSLAVSCPLTSLVSSRSFQTSAISRDI
DTAAKFIGAGAATVGVAGSGAGIGTVFGSLIIGYARNPSLKQQLFSYAILGFALSEAMGLFCLMVAFL
ILFAM

DIOPT alignment:
https://www.flyrnai.org/cgi-bin/DRSC_prot_align.pl?geneid1=43693&geneid2=517

- Alignment Length: 142
- Identity: 91/142 - (**64%**)
- Similarity: 108/142 - (**76%**)
- Gaps: 14/142 - (9%)

EMBOSS alignment:

- Matrix: EBLOSUM62
- Gap_penalty: 10.0
- Extend_penalty: 0.5
- Length: 149
- Identity: 91/149 (**61.1%**)
- Similarity: 108/149 (**72.5%**)
- Gaps: 19/149 (12.8%)
- Score: 424.0

```
ATPsynC    1 -----MFVSTVSRIAPVARSAFLANSKQYLRPLSSAIISQSQTLAAQN--
43
                  .||||.|.:.        :.|:...||||:.::.:.:.|..::
ATP5MC2    1 MFACSKFVSTPSLVK--------STSQLLSRPLSAVVLKRPEILTDESLS
42

ATPsynC   44 ----TTPVALLPQIRSFQTSPVTRDIDSAAKFIGAGAATVGVAGSGAGIG
89
                 :.|:..|...||||||.::||||:||||||||||||||||||||||
ATP5MC2   43 SLAVSCPLTSLVSSRSFQTSAISRDIDTAAKFIGAGAATVGVAGSGAGIG
92

ATPsynC   90 TVFGSLIIGYARNPSLKQQLFSYAILGFALSEAMGLFCLMMAFLLLFAF     138
             ||||||||||||||||||||||||||||||||||||||||:|||:|||.
ATP5MC2   93 TVFGSLIIGYARNPSLKQQLFSYAILGFALSEAMGLFCLMVAFLILFAM     141
```

> Drp1 (FBtr0335173 Drp1-RB 2868 735aa)

MEALIPVINKLQDVFNTVGSDSIQLPQIVVLGSQSSGKSSVIESVVGRSFLPRGTGIVTR
RPLVLQLIYSPLDDRENRSAENGTSNAEEWGRFLHTKKCFTDFDEIRKEIENETERAAGS
NKGICPEPINLKIFSTHVVNLTLVDLPGITKVPVGDQPEDIEAQIKELVLKYIENPNSII
LAVTAANTDMATSEALKLAKDVDPDGRRTLAVVTKLDLMDAGTDAIDILCGRVIPVKLGI
IGVMNRSQKDIMDQKHIDDQMKDEAAFLQRKYPTLATRNGTPYLAKTLNRLLMHHIRDCL
PDLKTRVNIMATQFQSLLNSYGEDVSDKSQTLLQIITKFSSAYCCTIEGTARNIETTELC
GGARMGYIFHETFGRTLDSIHPLAGLSKMDILTAIRNATGPRPALFVPEVSFELLVKRQI
RRLEEPSLRCVELIHEEMQRIVQHCGNEVQQEMLRFPKLHEKIVDVVTQLLRRRLPHTNV
MVENIVAIELAYINTKHPDFHKDAALVPSLLKTDSDPYSQINLGQRRANTPRNHMSPQIS
SHSAGSQQPQQQQPPQPNSSQQQYSQVHEQNHVAENSTPSMASTWLSNILPPAPTRPDSI
ENSTNNTPVHNNIVSPVKPVNLLPDVPANHNPRRLTDKEQKDCDVIEHLIKSYFYIVRKS
IQDSVPKAIMHFLVNYVKDNLQSELVTHLYKSDKAETLLNESDHIAVRRKEAADMLKALT
RANHIISEIRETHMW

> DNM1L (ENST00000553257.6 DNM1L-229 4553 749aa)

MEALIPVINKLQDVFNTVGADIIQLPQIVVVGTQSSGKSSVLESLVGRDLLPRGTGIVTR
RPLILQLVHVSQEDKRKTTGEENDPATWKNSRHLSKGVEAEEWGKFLHTKNKLYTDFDEI
RQEIENETERISGNNKGVSPEPIHLKIFSPNVVNLTLVDLPGMTKVPVGDQPKDIELQIR
ELILRFISNPNSIILAVTAANTDMATSEALKISREVDPDGRRTLAVITKLDLMDAGTDAM
DVLMGRVIPVKLGIIGVVNRSQLDINNKKSVTDSIRDEYAFLQKKYPSLANRNGTKYLAR
TLNRLLMHHIRDCLPELKTRINVLAAQYQSLLNSYGEPVDDKSATLLQLITKFATEYCNT
IEGTAKYIETSELCGGARICYIFHETFGRTLESVDPLGGLNTIDILTAIRNATGPRPALF
VPEVSFELLVKRQIKRLEEPSLRCVELVHEEMQRIIQHCSNYSTQELLRFPKLHDAIVEV
VTCLLRKRLPVTNEMVHNLVAIELAYINTKHPDFADACGLMNNNIEEQRRNRLARELPSA
VSRDKSSKVPSALAPASQEPSPAASAEADGKLIQDSRRETKNVASGGGGVGDGVQEPTTG
NWRGMLKTSKAEELLAEEKSKPIPIMPASPQKGHAVNLLDVPVPVARKLSAREQRDCEVI
ERLIKSYFLIVRKNIQDSVPKAVMHFLVNHVKDTLQSELVGQLYKSSLLDDLLTESEDMA
QRRKEAADMLKALQGASQIIAEIRETHLW

DIOPT alignment:
https://www.flyrnai.org/cgi-bin/DRSC_prot_align.pl?geneid1=33445&geneid2=10059

- Alignment Length: 772
- Identity: 490/772 - (**63%**)
- Similarity: 589/772 - (**76%**)
- Gaps: 60/772 - (7%)

EMBOSS alignment:

- Matrix: EBLOSUM62
- Gap_penalty: 10.0
- Extend_penalty: 0.5
- Length: 772
- Identity: 490/772 (**63.5%**)
- Similarity: 589/772 (**76.3%**)
- Gaps: 60/772 (7.8%)
- Score: 2386.5

```
Drp1     1 MEALIPVINKLQDVFNTVGSDSIQLPQIVVLGSQSSGKSSVIESVVGRSF     50
             |||||||||||||||||||:|.||||||||:|:||||||||:||:|||..
DNM1L    1 MEALIPVINKLQDVFNTVGADIIQLPQIVVVGTQSSGKSSVLESLVGRDL     50

Drp1    51 LPRGTGIVTRRPLVLQLIY-SPLDDRENRSAENGTS------------NA     87
             |||||||||||||:|||:: |..|.|:....||..:            .|
DNM1L   51 LPRGTGIVTRRPLILQLVHVSQEDKRKTTGEENDPATWKNSRHLSKGVEA    100

Drp1    88 EEWGRFLHTK-KCFTDFDEIRKEIENETERAAGSNKGICPEPINLKIFST    136
             ||||:||||| |.:|||||||:||||||||.:|:|||:.||||:|||||.
DNM1L  101 EEWGKFLHTKNKLYTDFDEIRQEIENETERISGNNKGVSPEPIHLKIFSP    150

Drp1   137 HVVNLTLVDLPGITKVPVGDQPEDIEAQIKELVLKYIENPNSIILAVTAA    186
             :|||||||||||:|||||||||:|||.||:||:|::|.||||||||||||
DNM1L  151 NVVNLTLVDLPGMTKVPVGDQPKDIELQIRELILRFISNPNSIILAVTAA    200

Drp1   187 NTDMATSEALKLAKDVDPDGRRTLAVVTKLDLMDAGTDAIDILCGRVIPV    236
             |||||||||||::::|||||||||||:||||||||||||:|:|.||||||
DNM1L  201 NTDMATSEALKISREVDPDGRRTLAVITKLDLMDAGTDAMDVLMGRVIPV    250

Drp1   237 KLGIIGVMNRSQKDIMDQKHIDDQMKDEAAFLQRKYPTLATRNGTPYLAK    286
             |||||||:||||.||.::|.:.|.::||.||||:|||:||.||||.|||:
DNM1L  251 KLGIIGVVNRSQLDINNKKSVTDSIRDEYAFLQKKYPSLANRNGTKYLAR    300

Drp1   287 TLNRLLMHHIRDCLPDLKTRVNIMATQFQSLLNSYGEDVSDKSQTLLQII    336
             |||||||||||||||:||||:|::|.|:|||||||||.|.|||.||||:|
DNM1L  301 TLNRLLMHHIRDCLPELKTRINVLAAQYQSLLNSYGEPVDDKSATLLQLI    350

Drp1   337 TKFSSAYCCTIEGTARNIETTELCGGARMGYIFHETFGRTLDSIHPLAGL    386
             |||::.||.||||||:.|||:|||||||:.|||||||||||:|:.||.||
DNM1L  351 TKFATEYCNTIEGTAKYIETSELCGGARICYIFHETFGRTLESVDPLGGL    400

Drp1   387 SKMDILTAIRNATGPRPALFVPEVSFELLVKRQIRRLEEPSLRCVELIHE    436
             :.:|||||||||||||||||||||||||||||||:||||||||||||:||
DNM1L  401 NTIDILTAIRNATGPRPALFVPEVSFELLVKRQIKRLEEPSLRCVELVHE    450

Drp1   437 EMQRIVQHCGNEVQQEMLRFPKLHEKIVDVVTQLLRRRLPHTNVMVENIV    486
```

```
           |||||:|||.|...||:|||||||:.||:|||.|||:|||.||.||.|:|
DNM1L 451  EMQRIIQHCSNYSTQELLRFPKLHDAIVEVVTCLLRKRLPVTNEMVHNLV    500

Drp1  487  AIELAYINTKHPDFHKDAALVPSLLKTDSDPYSQINLGQRRANTPRNHMS    536
           ||||||||||||||.....|:.:            |:.::|.|.....:
DNM1L 501  AIELAYINTKHPDFADACGLMNN------------NIEEQRRNRLAREL-    537

Drp1  537  PQISSHSAGSQQPQ----QQQPPQPNSSQQQYSQVHEQNHVAENSTPSMA    582
           |...|....|:.|.    ..|.|.|.:|.:...::.:.   :...|.::|
DNM1L 538  PSAVSRDKSSKVPSALAPASQEPSPAASAEADGKLIQD---SRRETKNVA    584

Drp1  583  S---------------TWLSNILPPAPTRPDSIENSTNNTPVHNNIVSPV    617
           |               .|...:   ..::.:.:.....:.|:.....||.
DNM1L 585  SGGGGVGDGVQEPTTGNWRGML---KTSKAEELLAEEKSKPIPIMPASPQ    631

Drp1  618  K--PVNLLPDVPANHNP--RRLTDKEQKDCDVIEHLIKSYFYIVRKSIQD    663
           |  .|||| |||.   |  |:|:.:||:||:|||.||||||.||||:|||
DNM1L 632  KGHAVNLL-DVPV---PVARKLSAREQRDCEVIERLIKSYFLIVRKNIQD    677

Drp1  664  SVPKAIMHFLVNYVKDNLQSELVTHLYKSDKAETLLNESDHIAVRRKEAA    713
           |||||:||||||:|||.||||||..||||...:.||.||:.:|.||||||
DNM1L 678  SVPKAVMHFLVNHVKDTLQSELVGQLYKSSLLDDLLTESEDMAQRRKEAA    727

Drp1  714  DMLKALTRANHIISEIRETHMW    735
           ||||||..|:.||:|||||||:|
DNM1L 728  DMLKALQGASQIIAEIRETHLW    749
```

> Fh (FBtr0071348, fh-RA, 923nt, 190aa)

MFAGRLMVRSIVGRACLATMGRWSKPQAHASQVILPSTPAIAAVAIQCEEFTANRRLFSSQIETESTL
DGATYERVCSDTLDALCDYFEELTENASELQGTDVAYSDGVLTVNLGGQHGTYVINRQTPNKQIWLSS
PTSGPKRYDFVGTVAAGRWIYKHSGQSLHELLQQEIPGILKSQSVDFLRLPYCS

> FXN (ENST00000484259.3, FXN-203, 6978nt, 210aa)

MWTLGRRAVAGLLASPSPAQAQTLTRVPRPAELAPLCGRRGLRTDIDATCTPRRASSNQRGLNQIWNV
KKQSVYLMNLRKSGTLGHPGSLDETTYERLAEETLDSLAEFFEDLADKPYTFEDYDVSFGSGVLTVKL
GGDLGTYVINKQTPNKQIWLSSPSSGPKRYDWTGKNWVYSHDGVSLHELLAAELTKALKTKLDLSSLA
YSGKDA

DIOPT alignment:
https://www.flyrnai.org/cgi-bin/DRSC_prot_align.pl?geneid1=31845&geneid2=2395

- Alignment Length: 185
- Identity: 77/185 - (**41%**)
- Similarity: 105/185 - (**56%**)
- Gaps: 17/185 - (9%)

EMBOSS alignment:

- Matrix: EBLOSUM62
- Gap_penalty: 10.0
- Extend_penalty: 0.5
- Length: 227
- Identity: 77/227 (**33.9%**)
- Similarity: 105/227 (**46.3%**)
- Gaps: 54/227 (23.8%)
- Score: 337.0

```
Fh     1 ----------------------------------MFAGRLMVRSIVGRAC     16
                                           ...||..:|:.:...|
FXN    1 MWTLGRRAVAGLLASPSPAQAQTLTRVPRPAELAPLCGRRGLRTDIDATC     50

Fh    17 LATMGRWSKPQAHASQVILPSTPAIAAVAIQCEEFTANRRLFSSQIETES     66
           |..|.|..|...:|:......::         :..|.|. |..:....
FXN   51 --TPRRASSNQRGLNQIWNVKKQSV---------YLMNLRK-SGTLGHPG     88

Fh    67 TLDGATYERVCSDTLDALCDYFEELTENASELQGTDVAYSDGVLTVNLGG    116
         :||..||||:..:|||:|.::||:|.:.....:..||::..|||||.|||
FXN   89 SLDETTYERLAEETLDSLAEFFEDLADKPYTFEDYDVSFGSGVLTVKLGG    138

Fh   117 QHGTYVINRQTPNKQIWLSSPTSGPKRYDFVGTVAAGRWIYKHSGQSLHE    166
         ..||||||:||||||||||||:|||||||:.|.    .|:|.|.|.||||
FXN  139 DLGTYVINKQTPNKQIWLSSPSSGPKRYDWTGK----NWVYSHDGVSLHE    184

Fh   167 LLQQEIPGILKSQSVDFLRLPYCS---    190
         ||..|:...||:: :|...|.|..
FXN  185 LLAAELTKALKTK-LDLSSLAYSGKDA    210
```

> Marf (FBtr0343373 Marf-RDn, 3617nt, 814aa)

MAAYLNRTISMVTGQTGPADDDRHASSTDTVDKSGPGSPLSRFNSSLQQSGSTMAANLLPESRLYQSN
DKSPLQIFVRAKKKINDIYGEIEEYVHETTTFINALHAEAEIVDKAERELFESYVYKVAAIREVLQRD
HMKVAFFGRTSNGKSSVINAMLREKILPSGIGHTTNCFCQVEGSNGGEAYLMTEGSEEKLNVVNIKQL
ANALCQEKLCESSLVRIFWPRERCSLLRDDVVFVDSPGVDVSANLDDWIDNHCLNADVFVLVLNAEST
MTRAEKQFFHTVSQKLSKPNIFILNNRWDASANEPECQESELAKVKSQHTERCIDFLTKELKVSNEKE
AAERVFFVSARETLQARIEEAKGNPPHMGAIAEGFQIRYFEFQDFERKFEECISQSAVKTKFQQHSSR
GKSVSGDMKSMLDNIYERITIFRNLKQDQKNLLTERIQGTETQMMQVTREMKMKIHNMVEEVEEKVSK
ALNEEIWRLGVLIDEFNMPFHPERLVLNIYKKELNAHVESGLGSNLRARLSMALAMNVESAQTEMTDR
MHALVPNEQLLATSTKMVVRTQPFEMLYSLNCQNLCADFQEDLEFKFSWGIAAMIQRFTGKVRERSKK
GQPALVNRQSSIGHSVSTPTTTPVEATPVCLLPAPVVAGITPEQLSLISRFAVSSIGSQGTVGGLVVA

GVMLKTIGWRVLVGVGALYGCIYLYERLSWTNSAKERTFKSQYVRHATKKLKMIVDLTSANCSHQVQQ
ELSSTFARLCRTVDTATTDMNDELKTLDSQLNILEANQKQLKLLRNKANYIQNELDIFEHNYISPQ

> MFN2 (ENST00000675817.1, MFN2-225, 4766 nt, 801aa)

MSLLFSRCNSIVTVKKNKRHMAEVNASPLKHFVTAKKKINGIFEQLGAYIQESATFLEDTYRNAELDP
VTTEEQVLDVKGYLSKVRGISEVLARRHMKVAFFGRTSNGKSTVINAMLWDKVLPSGIGHTTNCFLRV
EGTDGHEAFLLTEGSEEKRSAKTVNQLAHALHQDKQLHAGSLVSVMWPNSKCPLLKDDLVLMDSPGID
VTTELDSWIDKFCLDADVFVLVANSESTLMQTEKHFFHKVSERLSRPNIFILNNRWDASASEPEYMEE
VRRQHMERCTSFLVDELGVVDRSQAGDRIFFVSAKEVLNARIQKAQGMPEGGGALAEGFQVRMFEFQN
FERRFEECISQSAVKTKFEQHTVRAKQIAEAVRLIMDSLHMAAREQHALPVLCPPPDPHRRLKSAWLA
AGSSSYLGRGRPCPWVRVCSPARVYCEEMREERQDRLKFIDKQLELLAQDYKLRIKQITEEVERQVST
AMAEEIRRLSVLVDDYQMDFHPSPVVLKVYKNELHRHIEEGLGRNMSDRCSTAITNSLQTMQQDMIDG
LKPLLPVSVRSQIDMLVPRQCFSLNYDLNCDKLCADFQEDIEFHFSLGWTMLVNRFLGPKNSRRALMG
YNDQVQRPIPLTPANPSMPPLPQGSLTQEEFMVSMVTGLASLTSRTSMGILVVGGVVWKAVGWRLIAL
SFGLYGLLYVYERLTWTTKAKERAFKRQFVEHASEKLQLVISYTGSNCSHQVQQELSGTFAHLCQQVD
VTRENLEQEIAAMNKKIEVLDSLQSKAKLLRNKAGWLDSELNMFTHQYLQPSR

DIOPT alignment:
https://www.flyrnai.org/cgi-bin/DRSC_prot_align.pl?geneid1=31581&geneid2=9927

- Alignment Length: 752
- Identity: 354/752 - (**47%**)
- Similarity: 508/752 - (**67%**)
- Gaps: 32/752 - (4%)

EMBOSS alignment:

- Matrix: EBLOSUM62
- Gap_penalty: 10.0
- Extend_penalty: 0.5
- Length: 864
- Identity: 361/864 (**41.8%**)
- Similarity: 515/864 (**59.6%**)
- Gaps: 113/864 (13.1%)
- Score: 1773.5

```
Marf    1 MAAYLNRTISMVTGQTGPADDDRHASSTDTVDKSGPGSPLSRFNSSLQQS     50
                                                      .||..|
MFN2    1 --------------------------------------------MSLLFS      6

Marf   51 GSTMAANLLPESRLYQSNDKSPLQIFVRAKKKINDIYGEIEEYVHETTTF    100
          .......:....|.....:.|||:.||.||||||.|:.::..|:.|:.||
MFN2    7 RCNSIVTVKKNKRHMAEVNASPLKHFVTAKKKINGIFEQLGAYIQESATF     56

Marf  101 INALHAEAEI--VDKAEREL-FESYVYKVAAIREVLQRDHMKVAFFGRTS    147
          :...:..||:  |...|:.| .:.|:.||..|.|||.|.|||||||||||
MFN2   57 LEDTYRNAELDPVTTEEQVLDVKGYLSKVRGISEVLARRHMKVAFFGRTS    106
```

```
Marf 148 NGKSSVINAMLREKILPSGIGHTTNCFCQVEGSNGGEAYLMTEGSEEKLN    197
         ||||:||||||.:|:||||||||||||.:|||::|.||:|:|||||||.:
MFN2 107 NGKSTVINAMLWDKVLPSGIGHTTNCFLRVEGTDGHEAFLLTEGSEEKRS    156

Marf 198 VVNIKQLANALCQEK-LCESSLVRIFWPRERCSLLRDDVVFVDSPGVDVS    246
         ...:.|||:||.|:| |...|||.:.||..:|.||:||:|.:||||:||:
MFN2 157 AKTVNQLAHALHQDKQLHAGSLVSVMWPNSKCPLLKDDLVLMDSPGIDVT    206

Marf 247 ANLDDWIDNHCLNADVFVLVLNAESTMTRAEKQFFHTVSQKLSKPNIFIL    296
         ..||.|||..||:|||||||.|:||||:.:.||.|||.||::||:|||||
MFN2 207 TELDSWIDKFCLDADVFVLVANSESTLMQTEKHFFHKVSERLSRPNIFIL    256

Marf 297 NNRWDASANEPECQESELAKVKSQHTERCIDFLTKELKVSNEKEAAERVF    346
         ||||||||:||||..|    :|:.||.|||..||..||.|.:..:|.:|:|
MFN2 257 NNRWDASASEPEYME----EVRRQHMERCTSFLVDELGVVDRSQAGDRIF    302

Marf 347 FVSARETLQARIEEAKGNPPHMGAIAEGFQIRYFEFQDFERKFEECISQS    396
         ||||:|.|.|||::|:|.|...||:||||||:|.||||:|||:||||||||
MFN2 303 FVSAKEVLNARIQKAQGMPEGGGALAEGFQVRMFEFQNFERRFEECISQS    352

Marf 397 AVKTKFQQHSSRGKSVSGDMKSMLDNIY----ERITI---------FRNL    433
         ||||||:||:.|.|.::..::.::|:::    |:..:         .|.|
MFN2 353 AVKTKFEQHTVRAKQIAEAVRLIMDSLHMAAREQHALPVLCPPPDPHRRL    402

Marf 434 K------------------------------QDQKNLLTERIQGTETQM    452
         |                              ::.:....:|::..:.|:
MFN2 403 KSAWLAAGSSSYLGRGRPCPWVRVCSPARVYCEEMREERQDRLKFIDKQL    452

Marf 453 MQVTREMKMKIHNMVEEVEEKVSKALNEEIWRLGVLIDEFNMPFHPERLV    502
         ..:.::.|::|..:.||||.:||.|:.|||.||.||:|::.|.|||..:|
MFN2 453 ELLAQDYKLRIKQITEEVERQVSTAMAEEIRRLSVLVDDYQMDFHPSPVV    502

Marf 503 LNIYKKELNAHVESGLGSNLRARLSMALAMNVESAQTEMTDRMHALVPNE    552
         |.:||.||:.|:|.|||.|:..|.|.|:..::::.|.:|.|.:..|:|..
MFN2 503 LKVYKNELHRHIEEGLGRNMSDRCSTAITNSLQTMQQDMIDGLKPLLPVS    552

Marf 553 QLLATSTKMVVRTQPFEMLYSLNCQNLCADFQEDLEFKFSWGIAAMIQRF    602
           :.:...|:|..|.|.:.|.|||..||||||||:||.||.|...::.||
MFN2 553 --VRSQIDMLVPRQCFSLNYDLNCDKLCADFQEDIEFHFSLGWTMLVNRF    600

Marf 603 TGKVRERSKKGQPALVNRQSSIGHSVSTPTTTPVEATPVCLLPAPVVAG-    651
         .|     .|..:.||:.....:        ..|:..||......|:..|
MFN2 601 LG-----PKNSRRALMGYNDQV--------QRPIPLTPANPSMPPLPQGS    637

Marf 652 ITPEQLSLISRFAVSSIGSQGTVGGLVVAGVMLKTIGWRVLVGVGALYGC    701
         :|.|:..:....::|:.|:.::|.|||.||:.|.:|||::.....|||.
MFN2 638 LTQEEFMVSMVTGLASLTSRTSMGILVVGGVVWKAVGWRLIALSFGLYGL    687
```

```
Marf 702 IYLYERLSWTNSAKERTFKSQYVRHATKKLKMIVDLTSANCSHQVQQELS    751
         :|:||||:||..||||.||.|:|.||::||::::..|.:|||||||||||
MFN2 688 LYVYERLTWTTKAKERAFKRQFVEHASEKLQLVISYTGSNCSHQVQQELS    737

Marf 752 STFARLCRTVDTATTDMNDELKTLDSQLNILEANQKQLKLLRNKANYIQN    801
         .|||.||:.||....::..|:..::.::.:|::.|.:.|||||||.::.:
MFN2 738 GTFAHLCQQVDVTRENLEQEIAAMNKKIEVLDSLQSKAKLLRNKAGWLDS    787

Marf 802 ELDIFEHNYISPQ-    814
         ||::|.|.|:.|.
MFN2 788 ELNMFTHQYLQPSR    801
```

> mt:ATPase6 (FBtr0433498 mt:ATPase6-RB 675 224aa)

MMTNLFSVFDPSAIFNFSLNWLSTFLGLLMIPSIYWLMPSRYNIMWNSILLTLHKEFKTL
LGPSGHNGSTFIFISLFSLILFNNFMGLFPYIFTSTSHLTLTLSLALPLWLCFMLYGWIN
HTQHMFAHLVPQGTPAILMPFMVCIETISNIIRPGTLAVRLTANMIAGHLLLTLLGNTGP
SMSYILVTFLLMAQIALLVLESAVAMIQSYVFAVLSTLYSSEVN

> MT-ATP6 (ENST00000361899.2 MT-ATP6-201 681 226aa)

MNENLFASFIAPTILGLPAAVLIILFPPLLIPTSKYLINNRLITTQQWLIKLTSKQMMTM
HNTKGRTWSLMLVSLIIFIATTNLLGLLPHSFTPTTQLSMNLAMAIPLWAGTVIMGFRSK
IKNALAHFLPQGTPTPLIPMLVIIETISLLIQPMALAVRLTANITAGHLLMHLIGSATLA
MSTINLPSTLIIFTILILLTILEIAVALIQAYVFTLLVSLYLHDNT

DIOPT alignment:
https://www.flyrnai.org/cgi-bin/DRSC_prot_align.pl?geneid1=19893539&geneid2=4508

- Alignment Length: 227
- Identity: 87/227 - (**38%**)
- Similarity: 131/227 - (**57%**)
- Gaps: 14/227 - (6%)

EMBOSS alignment:
- Matrix: EBLOSUM62
- Gap_penalty: 10.0
- Extend_penalty: 0.5
- Length: 232
- Identity: 87/232 (**37.5%**)
- Similarity: 132/232 (**56.9%**)
- Gaps: 14/232 (6.0%)
- Score: 355.0

```
ATPase6    1 MMTNLFSVFDPSAIFNFSLNWLSTFLGLLMIPSIYWLMPSRYNIMWNSIL
50
             |..|||:.|....|.......|......|:||:..:|:.:|.......::
MT-ATP6    1 MNENLFASFIAPTILGLPAAVLIIILFPPLLIPTSKYLINNRLITTQQWLI
50

ATPase6   51 LTLHKEFKTLLGPSGHNGSTFIFISLFSLILF---NNFMGLFPYIFTSTS
97
             ....|:..|:....|...|    :.|.|||:|   .|.:||.|:.||.|:
MT-ATP6   51 KLTSKQMMTMHNTKGRTWS----LMLVSLIIFIATTNLLGLLPHSFTPTT
96

ATPase6   98 HLTLTLSLALPLWLCFMLYGWINHTQHMFAHLVPQGTPAILMPFMVCIET
147
             .|::.|::|:|||...::.|:.:..::..||.:|||||..|:|.:|.|||
MT-ATP6   97 QLSMNLAMAIPLWAGTVIMGFRSKIKNALAHFLPQGTPTPLIPMLVIIET
146

ATPase6  148 ISNIIRPGTLAVRLTANMIAGHLLLTLLGNTGPSMSYI-----LVTFLLM
192
             ||.:|:|..||||||||:.|||||:.|:|:...:||.|     |:.|.::
MT-ATP6  147 ISLLIQPMALAVRLTANITAGHLLMHLIGSATLAMSTINLPSTLIIFTIL
196

ATPase6  193 AQIALLVLESAVAMIQSYVFAVLSTLYSSEVN    224
               |.|.:||.|||:||:|||.:|.:||..:..
MT-ATP6  197 --ILLTILEIAVALIQAYVFTLLVSLYLHDNT    226
```

> Ogdh (FBtr0481643, Ogdh-RK, 4387 nt, 1010aa)

MHRAHTAFSLALSPMAHKNFATWLLKSSSSQQMAKVTAAAAVRTYNSAAAEPFANGSTASYVEEMYNA
WLRDPTSVHTSWDAYFRSNSYVSPPNLAPVQANTLPLTAFNFGGAVAGAAPDSKTIDDHLAVQAIIRS
YQIRGHNIAHLDPLEINTPELPGNSSTKSIYANFSFEPSEQDMDRQFKLPSTTFIGGDEASLPLKEIL
NRLENVYCNKIGVEFMFINSLEQCNWIRKRFETPGVLNFSPEEKRLILARLTRATGFEAFLAKKYSSE
KRFGLEGCEIMIPALKEIIDVSTELGVESVIMGMPHRGRLNTLANVCRKPLNQIFTQFAGLEAADDGS
GDVKYHLGTYIERLNRVTNKNIRLAVVANPSHLEAVDPVVQGKTRAEQFYRGDQEGKKVMSILIHGDA
AFCGQGVVYETMHLSDLPDYTTHGTIHVVANNQIGFTTDPRFSRSSPYCTDVARVVNAPIFHVNADDP
EAVMHVCKVAAEWRATFHKDCVIDLVGYRRNGHNEIDEPMFTQPLMYQKIRKHKNCLDLYADKLIAEG
TVTAEEVKSVAAKYENICEEAFALAKTETHVKYKDWLDSPWSGFFEGKDPLKVAPTGVKEETLIHIGN
RFSSPPPNAAEFVIHKGLLRVLAARKAMVDEKVADWALGEAMAFGSLLKEGIHVRLSGQDVERGTFSH
RHHVLHHQLVDKATYNSLQHMYPDQAPYSVSNSSLSEYAVLGFEHGYSMTNPNALVLWEAQFGDFSNT
AQSIIDQFISSGQSKWVRQSGLVMLLPHGMEGMGPEHSSCRVERFLQMSSDDPDYFPPESDEFGVRQL
HDINWIVANCSTPANYYHILRRQIALPFRKPLILCTPKSLLRHPEAKSPFSEMSEGSEFQRIIPDNGP

AGQNPSNVKKVVFCSGRVYYDLTKTRREKQLEGEIAIVRVEQISPFPFDLVKEQANLYKNAELVWAQE
EHKNQGSWTYVQPRFLTALNHSRDVSYVGRACGASTATGSKAQHIRELNALLNDAIST

> OGDH (ENST00000222673.6, OGDH-201, 4193nt, 1023aa)

MFHLRTCAAKLRPLTASQTVKTFSQNRPAAARTFQQIRCYSAPVAAEPFLSGTSSNYVEEMYCAWLEN
PKSVHKSWDIFFRNTNAGAPPGTAYQSPLPLSRGSLAAVAHAQSLVEAQPNVDKLVEDHLAVQSLIRA
YQIRGHHVAQLDPLGILDADLDSSVPADIISSTDKLGFYGLDESDLDKVFHLPTTTFIGGQESALPLR
EIIRRLEMAYCQHIGVEFMFINDLEQCQWIRQKFETPGIMQFTNEEKRTLLARLVRSTRFEEFLQRKW
SSEKRFGLEGCEVLIPALKTIIDKSSENGVDYVIMGMPHRGRLNVLANVIRKELEQIFCQFDSKLEAA
DEGSGDVKYHLGMYHRRINRVTDRNITLSLVANPSHLEAADPVVMGKTKAEQFYCGDTEGKKVMSILL
HGDAAFAGQGIVYETFHLSDLPSYTTHGTVHVVVNNQIGFTTDPRMARSSPYPTDVARVVNAPIFHVN
SDDPEAVMYVCKVAAEWRSTFHKDVVVDLVCYRRNGHNEMDEPMFTQPLMYKQIRKQKPVLQKYAELL
VSQGVVNQPEYEEEISKYDKICEEAFARSKDEKILHIKHWLDSPWPGFFTLDGQPRSMSCPSTGLTED
ILTHIGNVASSVPVENFTIHGGLSRILKTRGEMVKNRTVDWALAEYMAFGSLLKEGIHIRLSGQDVER
GTFSHRHHVLHDQNVDKRTCIPMNHLWPNQAPYTVCNSSLSEYGVLGFELGFAMASPNALVLWEAQFG
DFHNTAQCIIDQFICPGQAKWVRQNGIVLLLPHGMEGMGPEHSSARPERFLQMCNDDPDVLPDLKEAN
FDINQLYDCNWVVVNCSTPGNFFHVLRRQILLPFRKPLIIFTPKSLLRHPEARSSFDEMLPGTHFQRV
IPEDGPAAQNPENVKRLLFCTGKVYYDLTRERKARDMVGQVAITRIEQLSPFPFDLLLKEVQKYPNAE
LAWCQEEHKNQGYYDYVKPRLRTTISRAKPVWYAGRDPAAAPATGNKKTHLTELQRLLDTAFDLDVFK
NFS

DIOPT alignment:
https://www.flyrnai.org/cgi-bin/DRSC_prot_align.pl?geneid1=39899&geneid2=4967

- Alignment Length: 1122
- Identity: 627/1122 - (**55%**)
- Similarity: 768/1122 - (**68%**)
- Gaps: 135/1122 - (12%)

EMBOSS alignment:

- Matrix: EBLOSUM62
- Gap_penalty: 10.0
- Extend_penalty: 0.5
- Length: 1033
- Identity: 627/1033 (**60.7%**)
- Similarity: 769/1033 (**74.4%**)
- Gaps: 33/1033 (3.2%)
- Score: 3256.5

```
Ogdh    1 MHRAHTAFSLALSPMAHKNFATWLLKSSSSQQMAKVTAAAAVRTYNS-AA     49
          |....|. :..|.|:.    |:..:|:.|..:.|.......:|.|:: .|
OGDH    1 MFHLRTC-AAKLRPLT----ASQTVKTFSQNRPAAARTFQQIRCYSAPVA     45

Ogdh   50 AEPFANGSTASYVEEMYNAWLRDPTSVHTSWDAYFRSNSYVSPPNLA---     96
          ||||.:|::::||||||.|||.:|.|||.|||.:||:.:..:||..|
OGDH   46 AEPFLSGTSSNYVEEMYCAWLENPKSVHKSWDIFFRNTNAGAPPGTAYQS     95
```

```
Ogdh   97 PVQANTLPLTAFNFGGAVAGAAPD-SKTIDDHLAVQAIIRSYQIRGHNIA    145
          |:..:...|.|.....::..|.|: .|.::||||||::||:||||||::|
OGDH   96 PLPLSRGSLAAVAHAQSLVEAQPNVDKLVEDHLAVQSLIRAYQIRGHHVA    145

Ogdh  146 HLDPLEINTPELPGN------SSTKSIYANFSFEPSEQDMDRQFKLPSTT    189
          .||||.|...:|..:      |||..:  .| :...|.|:|:.|.||:||
OGDH  146 QLDPLGILDADLDSSVPADIISSTDKL--GF-YGLDESDLDKVFHLPTTT    192

Ogdh  190 FIGGDEASLPLKEILNRLENVYCNKIGVEFMFINSLEQCNWIRKRFETPG    239
          ||||.|::|||:||:.|||..||..|||||||||.||||.|||::|||||
OGDH  193 FIGGQESALPLREIIRRLEMAYCQHIGVEFMFINDLEQCQWIRQKFETPG    242

Ogdh  240 VLNFSPEEKRLILARLTRATGFEAFLAKKYSSEKRFGLEGCEIMIPALKE    289
          ::.|:.||||.:||||.|:|.||.||.:|:||||||||||||::|||||.
OGDH  243 IMQFTNEEKRTLLARLVRSTRFEEFLQRKWSSEKRFGLEGCEVLIPALKT    292

Ogdh  290 IIDVSTELGVESVIMGMPHRGRLNTLANVCRKPLNQIFTQF-AGLEAADD    338
          |||.|:|.||:.||||||||||||.||||.||.|.|||.|| :.|||||:
OGDH  293 IIDKSSENGVDYVIMGMPHRGRLNVLANVIRKELEQIFCQFDSKLEAADE    342

Ogdh  339 GSGDVKYHLGTYIERLNRVTNKNIRLAVVANPSHLEAVDPVVQGKTRAEQ    388
          ||||||||||.|..|:||||::||.|::|||||||.||||.|||:|||
OGDH  343 GSGDVKYHLGMYHRRINRVTDRNITLSLVANPSHLEAADPVVMGKTKAEQ    392

Ogdh  389 FYRGDQEGKKVMSILIHGDAAFCGQGVVYETMHLSDLPDYTTHGTIHVVA    438
          ||.||.|||||||||:||||||.|||:||||.||||||.||||||:|||.
OGDH  393 FYCGDTEGKKVMSILLHGDAAFAGQGIVYETFHLSDLPSYTTHGTVHVVV    442

Ogdh  439 NNQIGFTTDPRFSRSSPYCTDVARVVNAPIFHVNADDPEAVMHVCKVAAE    488
          |||||||||||.:|||||.|||||||||||||||:|||||||:|||||||
OGDH  443 NNQIGFTTDPRMARSSPYPTDVARVVNAPIFHVNSDDPEAVMYVCKVAAE    492

Ogdh  489 WRATFHKDCVIDLVGYRRNGHNEIDEPMFTQPLMYQKIRKHKNCLDLYAD    538
          ||:|||||.|:|||.||||||||:|||||||||||::|||.|..|..||:
OGDH  493 WRSTFHKDVVVDLVCYRRNGHNEMDEPMFTQPLMYKQIRKQKPVLQKYAE    542

Ogdh  539 KLIAEGTVTAEEVKSVAAKYENICEEAFALAKTETHVKYKDWLDSPWSGF    588
          .|:::|.|...|.:...:||:.|||||||.:|.|..:..|.||||||.||
OGDH  543 LLVSQGVVNQPEYEEEISKYDKICEEAFARSKDEKILHIKHWLDSPWPGF    592

Ogdh  589 F--EGKDPLKVAP-TGVKEETLIHIGNRFSSPPPNAAEFVIHKGLLRVLA    635
          |  :|:......| ||:.|:.|.||||..||.|  ...|.||.||.|:|.
OGDH  593 FTLDGQPRSMSCPSTGLTEDILTHIGNVASSVP--VENFTIHGGLSRILK    640

Ogdh  636 ARKAMVDEKVADWALGEAMAFGSLLKEGIHVRLSGQDVERGTFSHRHHVL    685
          .|..||..:..||||.|.||||||||||||:|||||||||||||||||||
OGDH  641 TRGEMVKNRTVDWALAEYMAFGSLLKEGIHIRLSGQDVERGTFSHRHHVL    690
```

```
Ogdh 686 HHQLVDKATYNSLQHMYPDQAPYSVSNSSLSEYAVLGFEHGYSMTNPNAL     735
         |.|.|||.|...:.|::|:||||:|.|||||||.|||||.|::|.:||||
OGDH 691 HDQNVDKRTCIPMNHLWPNQAPYTVCNSSLSEYGVLGFELGFAMASPNAL     740

Ogdh 736 VLWEAQFGDFSNTAQSIIDQFISSGQSKWVRQSGLVMLLPHGMEGMGPEH     785
         ||||||||||.||||.||||||..||:|||||:|:|:|||||||||||||
OGDH 741 VLWEAQFGDFHNTAQCIIDQFICPGQAKWVRQNGIVLLLPHGMEGMGPEH     790

Ogdh 786 SSCRVERFLQMSSDDPDYFPP-ESDEFGVRQLHDINWIVANCSTPANYYH     834
         ||.|.||||||.:||||..|. :...|.:.||:|.||:|.|||||.|::|
OGDH 791 SSARPERFLQMCNDDPDVLPDLKEANFDINQLYDCNWVVVNCSTPGNFFH     840

Ogdh 835 ILRRQIALPFRKPLILCTPKSLLRHPEAKSPFSEMSEGSEFQRIIPDNGP     884
         :|||||.||||||||:.|||||||||||:|.|.||..|:.|||:||::||
OGDH 841 VLRRQILLPFRKPLIIFTPKSLLRHPEARSSFDEMLPGTHFQRVIPEDGP     890

Ogdh 885 AGQNPSNVKKVVFCSGRVYYDLTKTRREKQLEGEIAIVRVEQISPFPFDL     934
         |.|||.|||:::||:|:||||||:.|:.:.:.:.|::||.|:||:|||||||
OGDH 891 AAQNPENVKRLLFCTGKVYYDLTRERKARDMVGQVAITRIEQLSPFPFDL     940

Ogdh 935 VKEQANLYKNAELVWAQEEHKNQGSWTYVQPRFLTALNHSRDVSYVGRAC     984
         :.::...|.||||.|.||||||||.:.||:||..|.::.::.|.|.||..
OGDH 941 LLKEVQKYPNAELAWCQEEHKNQGYYDYVKPRLRTTISRAKPVWYAGRDP     990

Ogdh 985 GASTATGSKAQHIRELNALLNDAIST-------   1010
         .|:.|||:|..|:.||..||:.|...
OGDH 991 AAAPATGNKKTHLTELQRLLDTAFDLDVFKNFS   1023
```

> Opa1 (FBtr0087574, Opa1-RA, 3217nt, 972aa)

```
MLRIYQNTYRRTARKAVVYSTKVACCNHSTLCGITSHPRRAQDSGSSSSNGRHRGHEEFLLAGNPARG
WQMPPPSRGYGMLVVRILRGALKLRYIVLGGAIGGGVSLSKKYEEWKDGLPNFKWLEDAMPQGERWSQ
FSRNLIEVGSLVKNAIEVDPKLKQLGEDKLSEWRNWFDSRLDDAIEAADYQGVQIVETKDDLKAKTTV
AALGITSDESRKKYEKLQSQVETLQTEIMNVQIKYQKELEKMEKENRELRQQYLILKTNKKTTAKKIK
KSLIDMYSEVLDELSGYDTGYTMADHLPRVVVVGDQSSGKTSVLESIAKARIFPRGSGEMMTRAPVKV
TLAEGPYHVAQFRDSDREYDLTKESDLQDLRRDVEFRMKASVRGGKTVSNEVIAMTVKGPGLQRMVLV
DLPGIISTMTVDMASDTKDSIHQMTKHYMSNPNAIILCIQDGSVDAERSNVTDLVMQCDPLGRRTIFV
LTKVDLAEELADPDRIRKILSGKLFPMKALGYYAVVTGRGRKDDSIDAIRQYEEDFFKNSKLFHRRGV
IMPHQVTSRNLSLAVSDRFWKMVRETIEQQADAFKATRFNLETEWKNNFPRLRESGRDELFDKAKGEI
LDEVVTLSQISAKKWDDALSTKLWEKLSNYVFESIYLPAAQSGSQNSFNTMVDIKLRQWAEQALPAKS
VEAGWEALQQEFISLMERSKKAQDHDGIFDQLKSAVVDEAIRRHSWEDKAIDMLRVIQLNTLEDRFVH
DKQEWDSAVKFLESSVNAKLVQTEETLAQMFGPGQMRRITHWQYLTQDQQKRRSVKNELDKILKNDTK
HLPTLTHDELTTVRKNLQRDNVDVDTDYIRQTWFPVYRKHFLQQALQRAKDCRKAYYLYTQQGAECEI
SCSDVVLFWRIQQVIKITGNALRQQVINREARRLDKEIKAVLDEFSDDEEKKGYLLTGKRVLLAEELI
KVRQIQEKLEEFINSLNQEK
```

> OPA1 (ENST00000361715.6, OPA1-203, 6384nt, 979aa)

```
MWRLRRAAVACEVCQSLVKHSSGIKGSLPLQKLHLVSRSIYHSHHPTLKLQRPQLRTSFQQFSSLTNL
PLRKLKFSPIKYGYQPRRNFWPARLATRLLKLRYLILGSAVGGGYTAKKTFDQWKDMIPDLSEYKWIV
PDIVWEIDEYIDFGHKLVSEVIGASDLLLLLGSPEETAFRATDRGSESDKHFRKGLLGELILLQQQIQ
EHEEEARRAAGQYSTSYAQQKRKVSDKEKIDQLQEELLHTQLKYQRILERLEKENKELRKLVLQKDDK
GIHHRKLKKSLIDMYSEVLDVLSDYDASYNTQDHLPRVVVVGDQSAGKTSVLEMIAQARIFPRGSGEM
MTRSPVKVTLSEGPHHVALFKDSSREFDLTKEEDLAALRHEIELRMRKNVKEGCTVSPETISLNVKGP
GLQRMVLVDLPGVINTVTSGMAPDTKETIFSISKAYMQNPNAIILCIQDGSVDAERSIVTDLVSQMDP
HGRRTIFVLTKVDLAEKNVASPSRIQQIIEGKLFPMKALGYFAVVTGKGNSSESIEAIREYEEEFFQN
SKLLKTSMLKAHQVTTRNLSLAVSDCFWKMVRESVEQQADSFKATRFNLETEWKNNYPRLRELDRNEL
FEKAKNEILDEVISLSQVTPKHWEEILQQSLWERVSTHVIENIYLPAAQTMNSGTFNTTVDIKLKQWT
DKQLPNKAVEVAWETLQEEFSRFMTEPKGKEHDDIFDKLKEAVKEESIKRHKWNDFAEDSLRVIQHNA
LEDRSISDKQQWDAAIYFMEEALQARLKDTENAIENMVGPDWKKRWLYWKNRTQEQCVHNETKNELEK
MLKCNEEHPAYLASDEITTVRKNLESRGVEVDPSLIKDTWHQVYRRHFLKTALNHCNLCRRGFYYYQR
HFVDSELECNDVVLFWRIQRMLAITANTLRQQLTNTEVRRLEKNVKEVLEDFAEDGEKKIKLLTGKRV
QLAEDLKKVREIQEKLDAFIEALHQEK
```

DIOPT alignment:
https://www.flyrnai.org/cgi-bin/DRSC_prot_align.pl?geneid1=36578&geneid2=4976

- Alignment Length: 966
- Identity: 503/966 - (**52%**)
- Similarity: 667/966 - (**69%**)
- Gaps: 69/966 - (7%)

EMBOSS alignment:

- Matrix: EBLOSUM62
- Gap_penalty: 10.0
- Extend_penalty: 0.5
- Length: 1007
- Identity: 515/1007 (**51.1%**)
- Similarity: 678/1007 (**67.3%**)
- Gaps: 63/1007 (6.3%)
- Score: 2463.0

```
Opa1    1 MLRIYQNTYRRTARKAVVYSTKVACCNHSTLCGIT-SHPRRAQDSGSSSS     49
                  ..|..|.||......:...||:  ||. |.|.:.....|.|.
OPA1    1 --------MWRLRRAAVACEVCQSLVKHSS--GIKGSLPLQKLHLVSRSI     40

Opa1   50 NGRHR------------GHEEF-LLAGNPARGWQMPPPSRGY----GMLV     82
          ...|.            ..::| .|...|.|..:..|...||    ....
OPA1   41 YHSHHPTLKLQRPQLRTSFQQFSSLTNLPLRKLKFSPIKYGYQPRRNFWP     90

Opa1   83 VRILRGALKLRYIVLGGAIGGGVSLSKKYEEWKDGLPN---FKWL-EDAM    128
          .|:....|||||::||.|:|||.:..|.:::|||.:|:   :||: .|.:
OPA1   91 ARLATRLLKLRYLILGSAVGGGYTAKKTFDQWKDMIPDLSEYKWIVPDIV    140
```

```
Opa1 129 PQGERWSQFSRNLIEVGSLVKNAIEVDPKLKQLGEDKLSEWRNWFDSRLD    178
         .:.:.:..|..      .||...|.....|..||..:.:.:|     ..|
OPA1 141 WEIDEYIDFGH------KLVSEVIGASDLLLLLGSPEETAFR-----ATD    179

Opa1 179 DAIEAADY--QGV---------QIVETKDDLKAKTTVAALGITSDESRKK    217
         ...|:..:  :|:         ||.|.:::.:   ..|....||...:|:
OPA1 180 RGSESDKHFRKGLLGELILLQQQIQEHEEEAR---RAAGQYSTSYAQQKR    226

Opa1 218 YEKLQSQVETLQTEIMNVQIKYQKELEKMEKENRELRQQYLIL-KTNKKT    266
         ....:.:::.||.|:::.|:|||:.||::||||:|||:  |:| |.:|..
OPA1 227 KVSDKEKIDQLQEELLHTQLKYQRILERLEKENKELRK--LVLQKDDKGI    274

Opa1 267 TAKKIKKSLIDMYSEVLDELSGYDTGYTMADHLPRVVVVGDQSSGKTSVL    316
         ..:|:|||||||||||||.||.||..|...|||||||||||||:||||||
OPA1 275 HHRKLKKSLIDMYSEVLDVLSDYDASYNTQDHLPRVVVVGDQSAGKTSVL    324

Opa1 317 ESIAKARIFPRGSGEMMTRAPVKVTLAEGPYHVAQFRDSDREYDLTKESD    366
         |.||:||||||||||||||:||||||:|||:|||.|:||.||:|||||.|
OPA1 325 EMIAQARIFPRGSGEMMTRSPVKVTLSEGPHHVALFKDSSREFDLTKEED    374

Opa1 367 LQDLRRDVEFRMKASVRGGKTVSNEVIAMTVKGPGLQRMVLVDLPGIIST    416
         |..||.::|.||:.:|:.|.|||.|.|::.||||||||||||||||:|:|
OPA1 375 LAALRHEIELRMRKNVKEGCTVSPETISLNVKGPGLQRMVLVDLPGVINT    424

Opa1 417 MTVDMASDTKDSIHQMTKHYMSNPNAIILCIQDGSVDAERSNVTDLVMQC    466
         :|..||.|||::|..::|.||.|||||||||||||||||||.|||||.|.
OPA1 425 VTSGMAPDTKETIFSISKAYMQNPNAIILCIQDGSVDAERSIVTDLVSQM    474

Opa1 467 DPLGRRTIFVLTKVDLAEE-LADPDRIRKILSGKLFPMKALGYYAVVTGR    515
         ||.|||||||||||||||: :|.|.||::|:.|||||||||||:|||||:
OPA1 475 DPHGRRTIFVLTKVDLAEKNVASPSRIQQIIEGKLFPMKALGYFAVVTGK    524

Opa1 516 GRKDDSIDAIRQYEEDFFKNSKLFHRRGVIMPHQVTSRNLSLAVSDRFWK    565
         |...:||:|||:|||:||:||||. :..::..||||:|||||||||.|||
OPA1 525 GNSSESIEAIREYEEEFFQNSKLL-KTSMLKAHQVTTRNLSLAVSDCFWK    573

Opa1 566 MVRETIEQQADAFKATRFNLETEWKNNFPRLRESGRDELFDKAKGEILDE    615
         ||||::|||||:|||||||||||||||:|||||..|:|||:|||.|||||
OPA1 574 MVRESVEQQADSFKATRFNLETEWKNNYPRLRELDRNELFEKAKNEILDE    623

Opa1 616 VVTLSQISAKKWDDALSTKLWEKLSNYVFESIYLPAAQSGSQNSFNTMVD    665
         |::|||::.|.|::.|...|||::|.:|.|:|||||||:.:..:|||.||
OPA1 624 VISLSQVTPKHWEEILQQSLWERVSTHVIENIYLPAAQTMNSGTFNTTVD    673

Opa1 666 IKLRQWAEQALPAKSVEAGWEALQQEFISLMERSKKAQDHDGIFDQLKSA    715
         |||:||.::.||.|:||..||.||:||...| ...|.::||.|||:||.|
OPA1 674 IKLKQWTDKQLPNKAVEVAWETLQEEFSRFM-TEPKGKEHDDIFDKLKEA    722
```

```
Opa1 716 VVDEAIRRHSWEDKAIDMLRVIQLNTLEDRFVHDKQEWDSAVKFLESSVN    765
         |.:|:|:||.|.|.|.|.|||||.|.||||.:.|||:||:|:.|:|.::.
OPA1 723 VKEESIKRHKWNDFAEDSLRVIQHNALEDRSISDKQQWDAAIYFMEEALQ    772

Opa1 766 AKLVQTEETLAQMFGPGQMRRITHWQYLTQDQQKRRSVKNELDKILKNDT    815
         |:|..||..:..|.||...:|..:|:..||:|......||||:|:||.:.
OPA1 773 ARLKDTENAIENMVGPDWKKRWLYWKNRTQEQCVHNETKNELEKMLKCNE    822

Opa1 816 KHLPTLTHDELTTVRKNLQRDNVDVDTDYIRQTWFPVYRKHFLQQALQRA    865
         :|...|..||:|||||||:...|:||...|:.||..|||:|||:.||...
OPA1 823 EHPAYLASDEITTVRKNLESRGVEVDPSLIKDTWHQVYRRHFLKTALNHC    872

Opa1 866 KDCRKAYYLYTQQGAECEISCSDVVLFWRIQQVIKITGNALRQQVINREA    915
         ..||:.:|.|.:...:.|:.|:|||||||||:::.||.|.||||:.|.|.
OPA1 873 NLCRRGFYYYQRHFVDSELECNDVVLFWRIQRMLAITANTLRQQLTNTEV    922

Opa1 916 RRLDKEIKAVLDEFSDDEEKKGYLLTGKRVLLAEELIKVRQIQEKLEEFI    965
         |||:|.:|.||::|::|.|||..|||||||.|||:|.|||:|||||:.||
OPA1 923 RRLEKNVKEVLEDFAEDGEKKIKLLTGKRVQLAEDLKKVREIQEKLDAFI    972

Opa1 966 NSLNQEK    972
         .:|:|||
OPA1 973 EALHQEK    979
```

> Pdha1 (FBtr0070711, Pdha-RC, 2034nt, 443aa)

MLRTLSRVSELPIIVKQLQKRCHNNNNNNIIISGCRILSNNNNNSSIDIIRNGRYSSSFFGLFQNAAQ
AGVSKTNNYATEATVQVNRPFKLHRLDEGPATEVKLTKDQALKYYTQMQTIRRLETAAGNLYKEKIIR
GFCHLYSGQEACAVGMKAAMRDVDNIISAYRVHGWTYLMGVSPSGVLAELTGVQGGCARGKGGSMHMY
APNFYGGNGIVGAQVPLGAGVGLACKYKGNGGMCLALYGDGAANQGQVFEAYNMAYLWKLPVIFVCEN
NNYGMGTSSERASCNTDYYTRGDALPGIWVDGMDVLAVRSATEFAINYVNTHGPLVMETNTYRYSGHS
MSDPGTSYRTREEIQEVRQKRDPITSFKELCIELGLITTDEVKAIDLKVRKEVDEATAFAKSDAELGV
SHLWTDVYSNNLEPKLRGTIAYDIDHIQERKGVNH

> PDHA2 (ENST00000295266.6, PDHA2-201, 1372nt, 388aa)

MLAAFISRVLRRVAQKSARRVLVASRNSSNDATFEIKKCDLYLLEEGPPVTTVLTRAEGLKYYRMMLT
VRRMELKADQLYKQKFIRGFCHLCDGQEACCVGLEAGINPSDHVITSYRAHGVCYTRGLSVRSILAEL
TGRRGGCAKGKGGSMHMYTKNFYGGNGIVGAQGPLGAGIALACKYKGNDEICLTLYGDGAANQGQIAE
AFNMAALWKLPCVFICENNLYGMGTSTERAAASPDYYKRGNFIPGLKVDGMDVLCVREATKFAANYCR
SGKGPILMELQTYRYHGHSMSDPGVSYRTREEIQEVRSKRDPIIILQDRMVNSKLATVEELKEIGAEV
RKEIDDAAQFATTDPEPHLEELGHHIYSSDSSFEVRGANPWIKFKSVS

> PDHA1 (ENST00000422285.7, PDHA1-205, 3349nt, 390aa)

MRKMLAAVSRVLSGASQKPASRVLVASRNFANDATFEIKKCDLHRLEEGPPVTTVLTREDGLKYYRMM
QTVRRMELKADQLYKQKIIRGFCHLCDGQEACCVGLEAGINPTDHLITAYRAHGFTFTRGLSVREILA

ELTGRKGGCAKGKGGSMHMYAKNFYGGNGIVGAQVPLGAGIALACKYNGKDEVCLTLYGDGAANQGQI
FEAYNMAALWKLPCIFICENNRYGMGTSVERAAASTDYYKRGDFIPGLRVDGMDILCVREATRFAAAY
CRSGKGPILMELQTYRYHGHSMSDPGVSYRTREEIQEVRSKSDPIMLLKDRMVNSNLASVEELKEIDV
EVRKEIEDAAQFATADPEPPLEELGYHIYSSDPPFEVRGANQWIKFKSVS

DIOPT v.9 alignment:
with PDH2: https://www.flyrnai.org/cgi-bin/DRSC_prot_align.pl?geneid1=31406&geneid2=5161

- Alignment Length: 364
- Identity: 206/364 - (**56%**)
- Similarity: 264/364 - (**72%**)
- Gaps: 2/364 - (0%)

with PDH1: https://www.flyrnai.org/cgi-bin/DRSC_prot_align.pl?geneid1=31406&geneid2=5160

- Alignment Length: 427
- Identity: 228/427 - (**53%**)
- Similarity: 291/427 - (**68%**)
- Gaps: 14/427 - (3%)

EMBOSS alignments:

- Matrix: EBLOSUM62
- Gap_penalty: 10.0
- Extend_penalty: 0.5
- Length: 457
- Identity: 207/457 (**45.3%**)
- Similarity: 271/457 (**59.3%**)
- Gaps: 83/457 (18.2%)
- Score: 1076.5

```
Pdha1    1 MLRTLSRVSELPIIVKQLQKRCHNNNNNNIIISGCRILSNNNNNSSIDII     50

PDHA2    1 --------------------------------------------------      0

Pdha1   51 RNGRYSSSFFG-----LFQNAAQAGVSKTNNYATEATVQVNRPFKLHRLD     95
               ..::|..     :.|.:|:..:..:.|.:.:||.::.: ..|:.|:
PDHA2    1 ----MLAAFISRVLRRVAQKSARRVLVASRNSSNDATFEIKK-CDLYLLE     45

Pdha1   96 EGPATEVKLTKDQALKYYTQMQTIRRLETAAGNLYKEKIIRGFCHLYSGQ    145
           |||.....||:.:.||||..|.|:||:|..|..|||:|.||||||||..||
PDHA2   46 EGPPVTTVLTRAEGLKYYRMMLTVRRMELKADQLYKQKFIRGFCHLCDGQ     95

Pdha1  146 EACAVGMKAAMRDVDNIISAYRVHGWTYLMGVSPSGVLAELTGVQGGCAR    195
           |||.||::|.:...|::|::||.||..|..|:|...:||||||.:||||:
PDHA2   96 EACCVGLEAGINPSDHVITSYRAHGVCYTRGLSVRSILAELTGRRGGCAK    145

Pdha1  196 GKGGSMHMYAPNFYGGNGIVGAQVPLGAGVGLACKYKGNGGMCLALYGDG    245
```

```
          |||||||||..||||||||||||.|||||:.||||||||..:||.|||||
PDHA2 146 GKGGSMHMYTKNFYGGNGIVGAQGPLGAGIALACKYKGNDEICLTLYGDG     195

Pdha1 246 AANQGQVFEAYNMAYLWKLPVIFVCENNNYGMGTSSERASCNTDYYTRGD     295
          ||||||:.||:|||.|||||.:|:||||.||||||:|||:.:.|||.||:
PDHA2 196 AANQGQIAEAFNMAALWKLPCVFICENNLYGMGTSTERAAASPDYYKRGN     245

Pdha1 296 ALPGIWVDGMDVLAVRSATEFAINYVNT-HGPLVMETNTYRYSGHSMSDP     344
          .:||:.|||||||.||.||:||.||..: .||::||..||||.|||||||
PDHA2 246 FIPGLKVDGMDVLCVREATKFAANYCRSGKGPILMELQTYRYHGHSMSDP     295

Pdha1 345 GTSYRTREEIQEVRQKRDPITSFKELCIELGLITTDEVKAIDLKVRKEVD     394
          |.||||||||||||.|||||...::..:...|.|.:|:|.|..:||||:|
PDHA2 296 GVSYRTREEIQEVRSKRDPIIILQDRMVNSKLATVEELKEIGAEVRKEID     345

Pdha1 395 EATAFAKSDAELGVSHLWTDVYSNNLEPKLRG--------TIAYDIDHIQ     436
          :|..||.:|.|..:..|...:||::...::||        :::
PDHA2 346 DAAQFATTDPEPHLEELGHHIYSSDSSFEVRGANPWIKFKSVS-------     388

Pdha1 437 ERKGVNH    443

PDHA2 389 -------    388
```

- Matrix: EBLOSUM62
- Gap_penalty: 10.0
- Extend_penalty: 0.5
- Length: 452
- Identity: 220/452 (**48.7%**)
- Similarity: 277/452 (**61.3%**)
- Gaps: 71/452 (15.7%)
- Score: 1133.0

```
Pdha1   1 MLRTLSRVSELPIIVKQLQKRCHNNNNNNIIISGCRILSNNNNNSSIDII      50
                                    ...::.:..|:||
PDHA1   1 --------------------------MRKMLAAVSRVLS-----------      13

Pdha1  51 RNGRYSSSFFGLFQNAAQAGVSKTNNYATEATVQVNRPFKLHRLDEGPAT     100
                    |..|..|...:..:.|:|.:||.::.: ..||||:|||..
PDHA1  14 ----------GASQKPASRVLVASRNFANDATFEIKK-CDLHRLEEGPPV      52

Pdha1 101 EVKLTKDQALKYYTQMQTIRRLETAAGNLYKEKIIRGFCHLYSGQEACAV     150
          ...||::..||||..|||:||:|..|..|||:|||||||||..|||||.|
PDHA1  53 TTVLTREDGLKYYRMMQTVRRMELKADQLYKQKIIRGFCHLCDGQEACCV     102

Pdha1 151 GMKAAMRDVDNIISAYRVHGWTYLMGVSPSGVLAELTGVQGGCARGKGGS     200
          |::|.:...|::|:|||.||:|:..|:|...:||||||.:||||:|||||
PDHA1 103 GLEAGINPTDHLITAYRAHGFTFTRGLSVREILAELTGRKGGCAKGKGGS     152
```

```
Pdha1 201 MHMYAPNFYGGNGIVGAQVPLGAGVGLACKYKGNGGMCLALYGDGAANQG    250
          |||||.||||||||||||||||||:.|||||.|...:||.||||||||||
PDHA1 153 MHMYAKNFYGGNGIVGAQVPLGAGIALACKYNGKDEVCLTLYGDGAANQG    202

Pdha1 251 QVFEAYNMAYLWKLPVIFVCENNNYGMGTSSERASCNTDYYTRGDALPGI    300
          |:|||||||.|||||.||:||||.||||||.|||:.:||||.|||.:||:
PDHA1 203 QIFEAYNMAALWKLPCIFICENNRYGMGTSVERAAASTDYYKRGDFIPGL    252

Pdha1 301 WVDGMDVLAVRSATEFAINYVNT-HGPLVMETNTYRYSGHSMSDPGTSYR    349
          .|||||:|.||.||.||..|..: .||::||..||||.||||||||.|||
PDHA1 253 RVDGMDILCVREATRFAAAYCRSGKGPILMELQTYRYHGHSMSDPGVSYR    302

Pdha1 350 TREEIQEVRQKRDPITSFKELCIELGLITTDEVKAIDLKVRKEVDEATAF    399
          |||||||||.|.|||...|:..:...|.:.:|:|.||::||||:::|..|
PDHA1 303 TREEIQEVRSKSDPIMLLKDRMVNSNLASVEELKEIDVEVRKEIEDAAQF    352

Pdha1 400 AKSDAELGVSHLWTDVYSNNLEPKLRGTIAYDIDHIQERKGVNH------    443
          |.:|.|..:..|...:||:  :|..            |.:|.|.
PDHA1 353 ATADPEPPLEELGYHIYSS--DPPF------------EVRGANQWIKFKS    388

Pdha1 444 --     443

PDHA1 389 VS     390
```

> QIL1 (FBtr0075220 QIL1-RA, 762nt, 122aa)

MVLGFLVRGGLVAATVYYTQKVGIWGDSDQTDKLYNDIKSELRPHVQKLEKQLPFEVPQLPKTGEMRF
LAKHYYNEGVKNTFRFIHMLPCYAGRGLKKVKDTFQDFAQSPAIAGGAESSPPK

> MICOS13 (ENST00000590389.5, MICOS13-206, 769nt, 110aa)

MRSECVLGAASDSGQEAPRDTWFLQGWKASRRFLIKGSVAGGAVYLVYDQELLGPSDKSQAALQKAGE
VVPPAMYQFSQYVCQQTGLQIPQLPAPPKIYFPIRDSWNAGI

DIOPT alignment:
https://www.flyrnai.org/cgi-bin/DRSC_prot_align.pl?geneid1=39948&geneid2=125988

- Alignment Length: 104
- Identity: 23/104 - (**22%**)
- Similarity: 44/104 - (**42%**)
- Gaps: 8/104 - (7%)

EMBOSS alignment:
Matrix: EBLOSUM62
Gap_penalty: 10.0

```
Extend_penalty: 0.5
#
Length: 154
Identity:      18/154 (11.7%)
Similarity:    35/154 (22.7%)
Gaps:          76/154 (49.4%)
Score: 64.5

QIL1       1 ----------------------------MVLGFLVRGGLVAATVYYTQKV
22
                                         ....||::|.:....||.....
MICOS13    1 MRSECVLGAASDSGQEAPRDTWFLQGWKASRRFLIKGSVAGGAVYLVYDQ
50

QIL1      23 GIWGDSDQTDKLYNDIKSELRPHV----QKLEKQLPFEVPQLPKTGEMRF
68
             .:.|.||::..........:.|.:    |.:.:|...::||||...::.|
MICOS13   51 ELLGPSDKSQAALQKAGEVVPPAMYQFSQYVCQQTGLQIPQLPAPPKIYF
100

QIL1      69 LAKHYYNEGVKNTFRFIHMLPCYAGRGLKKVKDTFQDFAQSPAIAGGAES
118
             ..:..:|.|:
MICOS13  101 PIRDSWNAGI----------------------------------------
110

QIL1     119 SPPK    122

MICOS13  111 ----    110
```

> SdhA (FBtr0086553, SdhA-RA, 2750nt, 661aa)

MSGIMRVPSILAKNAVASMQRAAAVGVQRSYHITHGRQQASAANPDKISKQYPVVDHAYDAIVVGAGG
AGLRAAFGLVAEGFRTAVITKLFPTRSHTIAAQGGINAALGNMEEDDWKWHMYDTVKGSDWLGDQDAI
HYMTREAPKAVIELENYGMPFSRTQDGKIYQRAFGGQSLKFGKGGQAHRCCAVADRTGHSLLHTLYGQ
SLSYDCNYFVEYFALDLIFEDGECRGVLALNLEDGTLHRFRAKNTVIATGGYGRAFFSCTSAHTCTGD
GTAMVARQGLPSQDLEFVQFHPTGIYGAGCLITEGCRGEGGYLINGNGERFMERYAPVAKDLASRDVV
SRSMTIEIMEGRGAGPEKDHVYLQLHHLPPKQLAERLPGISETAMIFAGVDVTREPIPVLPTVHYNMG
GVPTNYRGQVITIDKDGKDVIVPGLYAAGEAASSSVHGANRLGANSLLDLVVFGRACAKTIAELNKPG
APAPTLKENAGEASVANLDKLRHANGQITTADLRLKMQKTMQHHAAVFRDGPILQDGVNKMKEIYKQF
KDIKVVDRSLIWNSDLVETLELQNLLANAQMTIVSAEARKESRGAHAREDFKVREDEYDFSKPLDGQQ
KKPMDQHWRKHTLSWVCNDNGDITLDYRNVIDTTLDNEVSTVPPAIRSY

> SDHA (ENST00000264932.11, SDHA-201, 2693nt, 664aa)

MSGVRGLSRLLSARRLALAKAWPTVLQTGTRGFHFTVDGNKRASAKVSDSISAQYPVVDHEFDAVVVG
AGGAGLRAAFGLSEAGFNTACVTKLFPTRSHTVAAQGGINAALGNMEEDNWRWHFYDTVKGSDWLGDQ

DAIHYMTEQAPAAVVELENYGMPFSRTEDGKIYQRAFGGQSLKFGKGGQAHRCCCVADRTGHSLLHTL
YGRSLRYDTSYFVEYFALDLLMENGECRGVIALCIEDGSIHRIRAKNTVVATGGYGRTYFSCTSAHTS
TGDGTAMITRAGLPCQDLEFVQFHPTGIYGAGCLITEGCRGEGGILINSQGERFMERYAPVAKDLASR
DVVSRSMTLEIREGRGCGPEKDHVYLQLHHLPPEQLATRLPGISETAMIFAGVDVTKEPIPVLPTVHY
NMGGIPTNYKGQVLRHVNGQDQIVPGLYACGEAACASVHGANRLGANSLLDLVVFGRACALSIEESCR
PGDKVPPIKPNAGEESVMNLDKLRFADGSIRTSELRLSMQKSMQNHAAVFRVGSVLQEGCGKISKLYG
DLKHLKTFDRGMVWNTDLVETLELQNLMLCALQTIYGAEARKESRGAHAREDYKVRIDEYDYSKPIQG
QQKKPFEEHWRKHTLSYVDVGTGKVTLEYRPVIDKTLNEADCATVPPAIRSY

DIOPT v.9 alignment:
https://www.flyrnai.org/cgi-bin/DRSC_prot_align.pl?geneid1=37228&geneid2=6389

- Alignment Length: 642
- Identity: 474/642 - (**73%**)
- Similarity: 542/642 - (**84%**)
- Gaps: 6/642 - (0%)

EMBOSS alignment:

- Matrix: EBLOSUM62
- Gap_penalty: 10.0
- Extend_penalty: 0.5
- Length: 666
- Identity: 492/666 (**73.9%**)
- Similarity: 561/666 (**84.2%**)
- Gaps: 7/666 (1.1%)
- Score: 2599.0

```
SdhA    1 MSGIMRVPSILAKNAVASMQRAAAVGVQ---RSYHIT-HGRQQASAANPD     46
          |||:..:..:|:...:| :.:|....:|   |.:|.| .|.::|||...|
SDHA    1 MSGVRGLSRLLSARRLA-LAKAWPTVLQTGTRGFHFTVDGNKRASAKVSD     49

SdhA   47 KISKQYPVVDHAYDAIVVGAGGAGLRAAFGLVAEGFRTAVITKLFPTRSH     96
          .||.|||||||.:||:|||||||||||||||...||.||.:|||||||||
SDHA   50 SISAQYPVVDHEFDAVVVGAGGAGLRAAFGLSEAGFNTACVTKLFPTRSH     99

SdhA   97 TIAAQGGINAALGNMEEDDWKWHMYDTVKGSDWLGDQDAIHYMTREAPKA    146
          |:||||||||||||||||:|:||.||||||||||||||||||||.:||.|
SDHA  100 TVAAQGGINAALGNMEEDNWRWHFYDTVKGSDWLGDQDAIHYMTEQAPAA    149

SdhA  147 VIELENYGMPFSRTQDGKIYQRAFGGQSLKFGKGGQAHRCCAVADRTGHS    196
          |:||||||||||||:||||||||||||||||||||||||||.||||||||
SDHA  150 VVELENYGMPFSRTEDGKIYQRAFGGQSLKFGKGGQAHRCCCVADRTGHS    199

SdhA  197 LLHTLYGQSLSYDCNYFVEYFALDLIFEDGECRGVLALNLEDGTLHRFRA    246
          |||||||:||.||.:||||||||||:.|:||||||:||.:|||::||.||
SDHA  200 LLHTLYGRSLRYDTSYFVEYFALDLLMENGECRGVIALCIEDGSIHRIRA    249

SdhA  247 KNTVIATGGYGRAFFSCTSAHTCTGDGTAMVARQGLPSQDLEFVQFHPTG    296
```

```
          ||||:|||||||.:||||||||.|||||||:.|.|||.||||||||||||
SDHA 250 KNTVVATGGYGRTYFSCTSAHTSTGDGTAMITRAGLPCQDLEFVQFHPTG    299

SdhA 297 IYGAGCLITEGCRGEGGYLINGNGERFMERYAPVAKDLASRDVVSRSMTI    346
          |||||||||||||||||.|||..||||||||||||||||||||||||||:
SDHA 300 IYGAGCLITEGCRGEGGILINSQGERFMERYAPVAKDLASRDVVSRSMTL    349

SdhA 347 EIMEGRGAGPEKDHVYLQLHHLPPKQLAERLPGISETAMIFAGVDVTREP    396
          ||.||||.||||||||||||||||:|||.||||||||||||||||||:||
SDHA 350 EIREGRGCGPEKDHVYLQLHHLPPEQLATRLPGISETAMIFAGVDVTKEP    399

SdhA 397 IPVLPTVHYNMGGVPTNYRGQVITIDKDGKDVIVPGLYAAGEAASSSVHG    446
          |||||||||||||:||||:|||:. ..:|:|.|||||||.||||.:||||
SDHA 400 IPVLPTVHYNMGGIPTNYKGQVLR-HVNGQDQIVPGLYACGEAACASVHG    448

SdhA 447 ANRLGANSLLDLVVFGRACAKTIAELNKPGAPAPTLKENAGEASVANLDK    496
          ||||||||||||||||||||.:|.|..:||...|.:|.||||.||.||||
SDHA 449 ANRLGANSLLDLVVFGRACALSIEESCRPGDKVPPIKPNAGEESVMNLDK    498

SdhA 497 LRHANGQITTADLRLKMQKTMQHHAAVFRDGPILQDGVNKMKEIYKQFKD    546
          ||.|:|.|.|::|||.|||:||:||||||.|.:||:|..|:.::|...|.
SDHA 499 LRFADGSIRTSELRLSMQKSMQNHAAVFRVGSVLQEGCGKISKLYGDLKH    548

SdhA 547 IKVVDRSLIWNSDLVETLELQNLLANAQMTIVSAEARKESRGAHAREDFK    596
          :|..||.::||:|||||||||||:..|..||..|||||||||||||||:|
SDHA 549 LKTFDRGMVWNTDLVETLELQNLMLCALQTIYGAEARKESRGAHAREDYK    598

SdhA 597 VREDEYDFSKPLDGQQKKPMDQHWRKHTLSWVCNDNGDITLDYRNVIDTT    646
          ||.||||:|||:.||||||.::||||||||:|....|.:||:||.|||.|
SDHA 599 VRIDEYDYSKPIQGQQKKPFEEHWRKHTLSYVDVGTGKVTLEYRPVIDKT    648

SdhA 647 LDN-EVSTVPPAIRSY    661
          |:. :.:|||||||||
SDHA 649 LNEADCATVPPAIRSY    664
```

> SesB (FBtr0073424, sesB-RD, 1853nt, 312aa)

```
MGNISASITSQSKMGKDFDAVGFVKDFAAGGISAAVSKTAVAPIERVKLLLQVQHISKQISPDKQYKG
MVDCFIRIPKEQGFSSFWRGNLANVIRYFPTQALNFAFKDKYKQVFLGGVDKNTQFWRYFAGNLASGG
AAGATSLCFVYPLDFARTRLAADTGKGGQREFTGLGNCLTKIFKSDGIVGLYRGFGVSVQGIIIYRAA
YFGFYDTARGMLPDPKNTPIYISWAIAQVVTTVAGIVSYPFDTVRRRMMMQSGRKATEVIYKNTLHCW
ATIAKQEGTGAFFKGAFSNILRGTGGAFVLVLYDEIKKVL
```

> SLC25A4 (ENST00000281456.11, SLC25A4-201, 4415nt, 298aa)

MGDHAWSFLKDFLAGGVAAAVSKTAVAPIERVKLLLQVQHASKQISAEKQYKGIIDCVVRIPKEQGFL
SFWRGNLANVIRYFPTQALNFAFKDKYKQLFLGGVDRHKQFWRYFAGNLASGGAAGATSLCFVYPLDF
ARTRLAADVGKGAAQREFHGLGDCIIKIFKSDGLRGLYQGFNVSVQGIIIYRAAYFGVYDTAKGMLPD
PKNVHIFVSWMIAQSVTAVAGLVSYPFDTVRRRMMQSGRKGADIMYTGTVDCWRKIAKDEGAKAFFK
GAWSNVLRGMGGAFVLVLYDEIKKYV

DIOPT alignment:
https://www.flyrnai.org/cgi-bin/DRSC_prot_align.pl?geneid1=32007&geneid2=291

- Alignment Length: 292
- Identity: 236/292 - (**80%**)
- Similarity: 260/292 - (**89%**)
- Gaps: 1/292 - (0%)

EMBOSS alignment:

- Matrix: EBLOSUM62
- Gap_penalty: 10.0
- Extend_penalty: 0.5
- Length: 313
- Identity: 238/313 (**76.0%**)
- Similarity: 263/313 (**84.0%**)
- Gaps: 16/313 (5.1%)
- Score: 1249.5

```
SesB       1 MGNISASITSQSKMGKDFDAVGFVKDFAAGGISAAVSKTAVAPIERVKLL
50
                          ||.  .|..|:|||.|||::|||||||||||||||||
SLC25A4    1 -------------MGD--HAWSFLKDFLAGGVAAAVSKTAVAPIERVKLL
35

SesB      51 LQVQHISKQISPDKQYKGMVDCFIRIPKEQGFSSFWRGNLANVIRYFPTQ
100
             |||||.|||||.:|||||::||.:||||||||.|||||||||||||||||
SLC25A4   36 LQVQHASKQISAEKQYKGIIDCVVRIPKEQGFLSFWRGNLANVIRYFPTQ
85

SesB     101 ALNFAFKDKYKQVFLGGVDKNTQFWRYFAGNLASGGAAGATSLCFVYPLD
150
             ||||||||||||:||||||::.||||||||||||||||||||||||||||
SLC25A4   86 ALNFAFKDKYKQLFLGGVDRHKQFWRYFAGNLASGGAAGATSLCFVYPLD
135

SesB     151 FARTRLAADTGKG-GQREFTGLGNCLTKIFKSDGIVGLYRGFGVSVQGII
199
             |||||||||.||| .||||.|||:|:.|||||||:.|||:||.|||||||
SLC25A4  136 FARTRLAADVGKGAAQREFHGLGDCIIKIFKSDGLRGLYQGFNVSVQGII
185
```

```
SesB     200 IYRAAYFGFYDTARGMLPDPKNTPIYISWAIAQVVTTVAGIVSYPFDTVR
249
             ||||||||.||||:||||||||..|::||.|||.||.|||:|||||||||
SLC25A4  186 IYRAAYFGVYDTAKGMLPDPKNVHIFVSWMIAQSVTAVAGLVSYPFDTVR
235

SesB     250 RRMMMQSGRKATEVIYKNTLHCWATIAKQEGTGAFFKGAFSNILRGTGGA
299
             ||||||||||..:::|..|:.||..|||.||..||||||:||:|||.|||
SLC25A4  236 RRMMMQSGRKGADIMYTGTVDCWRKIAKDEGAKAFFKGAWSNVLRGMGGA
285

SesB     300 FVLVLYDEIKKVL    312
             |||||||||||.:
SLC25A4  286 FVLVLYDEIKKYV    298
```

> Sod2 (FBtr0345492, Sod2-RB, 1015nt, 217aa)

MFVARKISQTASLAVRGKHTLPKLPYDYAALEPIICREIMELHHQKHHQTYVNNLNAAEEQLEEAKSK
SDTTKLIQLAPALRFNGGGHINHTIFWQNLSPNKTQPSDDLKKAIESQWKSLEEFKKELTTLTVAVQG
SGWGWLGFNKKSGKLQLAALPNQDPLEASTGLIPLFGIDVWEHAYYLQYKNVRPSYVEAIWDIANWDD
ISCRFQEAKKLGC

> SOD2 (ENST00000538183.7, SOD2-210, 14167nt, 222aa)

MLSRAVCGTSRQLAPVLGYLGSRQKHSLPDLPYDYGALEPHINAQIMQLHHSKHHAAYVNNLNVTEEK
YQEALAKGDVTAQIALQPALKFNGGGHINHSIFWTNLSPNGGGEPKGELLEAIKRDFGSFDKFKEKLT
AASVGVQGSGWGWLGFNKERGHLQIAACPNQDPLQGTTGLIPLLGIDVWEHAYYLQYKNVRPDYLKAI
WNVINWENVTERYMACKK

DIOPT alignment:
https://www.flyrnai.org/cgi-bin/DRSC_prot_align.pl?geneid1=36878&geneid2=6648

- Alignment Length: 203
- Identity: 126/203 - (**62%**)
- Similarity: 157/203 - (**77%**)
- Gaps: 1/203 - (0%)

EMBOSS alignment:

- Matrix: EBLOSUM62
- Gap_penalty: 10.0
- Extend_penalty: 0.5
- Length: 226
- Identity: 128/226 (**56.6%**)
- Similarity: 163/226 (**72.1%**)

- Gaps: 13/226 (5.8%)
- Score: 703.5

```
Sod2   1 MFVARKISQTAS--------LAVRGKHTLPKLPYDYAALEPIICREIMEL     42
          .::|.:..|:.        |..|.||:||.|||||.||||.|..:||:|
SOD2   1 -MLSRAVCGTSRQLAPVLGYLGSRQKHSLPDLPYDYGALEPHINAQIMQL     49

Sod2  43 HHQKHHQTYVNNLNAAEEQLEEAKSKSDTTKLIQLAPALRFNGGGHINHT     92
         ||.|||..||||||..||:.:||.:|.|.|..|.|.|||:|||||||||:
SOD2  50 HHSKHHAAYVNNLNVTEEKYQEALAKGDVTAQIALQPALKFNGGGHINHS     99

Sod2  93 IFWQNLSPN-KTQPSDDLKKAIESQWKSLEEFKKELTTLTVAVQGSGWGW    141
         |||.||||| ..:|..:|.:||:..:.|.::||::||..:|.||||||||
SOD2 100 IFWTNLSPNGGGEPKGELLEAIKRDFGSFDKFKEKLTAASVGVQGSGWGW    149

Sod2 142 LGFNKKSGKLQLAALPNQDPLEASTGLIPLFGIDVWEHAYYLQYKNVRPS    191
         |||||:.|.||:||.||||||:.:||||||.||||||||||||||||||.
SOD2 150 LGFNKERGHLQIAACPNQDPLQGTTGLIPLLGIDVWEHAYYLQYKNVRPD    199

Sod2 192 YVEAIWDIANWDDISCRFQEAKKLGC    217
         |::|||::.||::::.|:...||
SOD2 200 YLKAIWNVINWENVTERYMACKK---    222
```

> YME1L (FBtr0304924, YME1L-RC, 2515nt, 740aa)

```
MFSTTTHSVPYLYLGNFSRKPHYYSVNRTKLHGSAGAARLSKSTSTSSRSHDLVLDLRNLLSRSSASI
QGMVERAARLNGILDRRLVDDVLAKVTSMLPSMRDVRVTLEESATQIGRVQLQNYQFEVSLTGAAGSV
PTGANVKVIPTITPGLLRPLFSQQQLNQIRGFKTDRSIEAEQKRNPTMTSRLKNALANSPQRLDGDTP
LQAEKLRRLLAKSEEHGFNKAESLKIAFAEGYLAAANSEDSPKSGKTMKYLKTLQTIVVIVVFLGIFL
SFFTTSNGSVFRSIQLGNQVEVDPEEINVTFEDVKGCDEAKQELKEVVEFLKSPEKFSNLGGKLPKGV
LLVGPPGTGKTLLARAVAGEAKVPFFHAAGPEFDEVLVGQGARRVRDLFKAAKARAPCVIFIDEIDSV
GAKRTNSVLHPYANQTINQLLSEMDGFHQNAGVIVLGATNRRDDLDQALLRPGRFDVEVMVSTPDFTG
RKEILSLYLTKILHDEIDLDMLARGTSGFTGADLENMINQAALRAAIDGAETVSMKHLETARDKVLMG
PERKARLPDEEANTITAYHEGGHAIVAFYTKESHPLHKVTIMPRGPSLGHTAYIPEKERYHVTKAQLL
AMMDTMMGGRAAEELVFGTDKITSGASSDLKQATSIATHMVRDWGMSDKVGLRTIEASKGLGTGDTLG
PNTIEAVDAEIKRILSDSYERAKAILRKHTREHKALAEALLKYETLDADDIKAILNESQT
```

> YME1L1 (ENST00000376016.8, YME1L1-202, 4191nt, 716aa)

```
MFSLSSTVQPQVTVPLSHLINAFHTPKNTSVSLSGVSVSQNQHRDVVPEHEAPSSEPSLNLRDLGLSE
LKIGQIDQLVENLLPGFCKGKNISSHWHTSHVSAQSFFENKYGNLDIFSTLRSSCLYRHHSRALQSIC
SDLQYWPVFIQSRGFKTLKSRTRRLQSTSERLAETQNIAPSFVKGFLLRDRGSDVESLDKLMKTKNIP
EAHQDAFKTGFAEGFLKAQALTQKTNDSLRRTRLILFVLLLFGIYGLLKNPFLSVRFRTTTGLDSAVD
PVQMKNVTFEHVKGVEEAKQELQEVVEFLKNPQKFTILGGKLPKGILLVGPPGTGKTLLARAVAGEAD
VPFYYASGSEFDEMFVGVGASRIRNLFREAKANAPCVIFIDELDSVGGKRIESPMHPYSRQTINQLLA
EMDGFKPNEGVIIIGATNFPEALDNALIRPGRFDMQVTVPRPDVKGRTEILKWYLNKIKFDQSVDPEI
```

IARGTVGFSGAELENLVNQAALKAAVDGKEMVTMKELEFSKDKILMGPERRSVEIDNKNKTITAYHES
GHAIIAYYTKDAMPINKATIMPRGPTLGHVSLLPENDRWNETRAQLLAQMDVSMGGRVAEELIFGTDH
ITTGASSDFDNATKIAKRMVTKFGMSEKLGVMTYSDTGKLSPETQSAIEQEIRILLRDSYERAKHILK
THAKEHKNLAEALLTYETLDAKEIQIVLEGKKLEVR

DIOPT alignment:
https://www.flyrnai.org/cgi-bin/DRSC_prot_align.pl?geneid1=37636&geneid2=10730

- Alignment Length: 599
- Identity: 319/599 - (**53%**)
- Similarity: 412/599 - (**68%**)
- Gaps: 51/599 - (8%)

EMBOSS alignment:

- Matrix: EBLOSUM62
- Gap_penalty: 10.0
- Extend_penalty: 0.5
- Length: 788
- Identity: 348/788 (**44.2%**)
- Similarity: 473/788 (**60.0%**)
- Gaps: 120/788 (15.2%)
- Score: 1502.0

```
YME1L      1 MFSTTTH-----SVPYLYLGNFSRKPHYYSVNRTKLHGSAGAARLSKSTS     45
             |||.::.     :||..:|.|....|...||:            ||..:.
YME1L1     1 MFSLSSTVQPQVTVPLSHLINAFHTPKNTSVS------------LSGVSV     38

YME1L     46 TSSRSHDLVLDLRNLLSRSSASIQGMVERAARLNGILDRRL--VDDVLAK     93
             :.::..|:|.:.....|..|.:::.:        |:.:.::  :|.:
YME1L1    39 SQNQHRDVVPEHEAPSSEPSLNLRDL--------GLSELKIGQIDQL---     77

YME1L     94 VTSMLPSM---RDVRVTLEESATQIGRVQLQNYQFEVSLTGAAGSVPTGA    140
             |.::||..   :::     .|......|..|:: ||...          .
YME1L1    78 VENLLPGFCKGKNI-----SSHWHTSHVSAQSF-FENKY----------G    111

YME1L    141 NVKVIPTITPGLLR-----------------PLFSQQQLNQIRGFKTDRS    173
             |:.:..|:....|.                 |:|.|.     |||||
YME1L1   112 NLDIFSTLRSSCLYRHHSRALQSICSDLQYWPVFIQS-----RGFKT---    153

YME1L    174 IEAEQKRNPTMTSRLKNALANSPQRLDG----DTPLQAEKLRRLLAKS--    217
             :::..:|..:.:.||......:|..:.|    |.....|.|.:|:...
YME1L1   154 LKSRTRRLQSTSERLAETQNIAPSFVKGFLLRDRGSDVESLDKLMKTKNI    203

YME1L    218 -EEHGFNKAESLKIAFAEGYLAAANSEDSPKSGKTMKYLKTLQTIVVIVV    266
              |.|    .::.|..||||:|.|     ...:.||...|:..:.|:.:::
YME1L1   204 PEAH----QDAFKTGFAEGFLKA-----QALTQKTNDSLRRTRLILFVLL    244
```

```
YME1L  267 FLGI-------FLS--FFTTSNGSVFRSIQLGNQVEVDPEEI-NVTFEDV    306
           ..||       |||  |.||:          |....|||.:: |||||.|
YME1L1 245 LFGIYGLLKNPFLSVRFRTTT----------GLDSAVDPVQMKNVTFEHV    284

YME1L  307 KGCDEAKQELKEVVEFLKSPEKFSNLGGKLPKGVLLVGPPGTGKTLLARA    356
           ||.:||||||:|||||||:|:||:.|||||||:|||||||||||||||||
YME1L1 285 KGVEEAKQELQEVVEFLKNPQKFTILGGKLPKGILLVGPPGTGKTLLARA    334

YME1L  357 VAGEAKVPFFHAAGPEFDEVLVGQGARRVRDLFKAAKARAPCVIFIDEID    406
           |||||.|||::|:|.||||:.||.||.|:|:||:.|||.|||||||||:|
YME1L1 335 VAGEADVPFYYASGSEFDEMFVGVGASRIRNLFREAKANAPCVIFIDELD    384

YME1L  407 SVGAKRTNSVLHPYANQTINQLLSEMDGFHQNAGVIVLGATNRRDDLDQA    456
           |||.||..|.:|||:.|||||||:|||||..|.|||::||||..:.||.|
YME1L1 385 SVGGKRIESPMHPYSRQTINQLLAEMDGFKPNEGVIIIGATNFPEALDNA    434

YME1L  457 LLRPGRFDVEVMVSTPDFTGRKEILSLYLTKILHDE-IDLDMLARGTSGF    505
           |:||||||::|.|..||..||.|||..||.||..|: :|.:::||||.||
YME1L1 435 LIRPGRFDMQVTVPRPDVKGRTEILKWYLNKIKFDQSVDPEIIARGTVGF    484

YME1L  506 TGADLENMINQAALRAAIDGAETVSMKHLETARDKVLMGPERKARLPDEE    555
           :||:|||::|||||:||:||.|.|:||.||.::||:||||||::...|.:
YME1L1 485 SGAELENLVNQAALKAAVDGKEMVTMKELEFSKDKILMGPERRSVEIDNK    534

YME1L  556 ANTITAYHEGGHAIVAFYTKESHPLHKVTIMPRGPSLGHTAYIPEKERYH    605
           ..|||||||.||||:|:|||::.|::|.|||||||:|||.:.:||.:|::
YME1L1 535 NKTITAYHESGHAIIAYYTKDAMPINKATIMPRGPTLGHVSLLPENDRWN    584

YME1L  606 VTKAQLLAMMDTMMGGRAAEELVFGTDKITSGASSDLKQATSIATHMVRD    655
           .|:|||||.||..||||.||||:||||.||:|||||...||.||..||..
YME1L1 585 ETRAQLLAQMDVSMGGRVAEELIFGTDHITTGASSDFDNATKIAKRMVTK    634

YME1L  656 WGMSDKVGLRTIEASKGLGTGDTLGPNTIEAVDAEIKRILSDSYERAKAI    705
           :|||:|:|:.|..     .|| .|.|.|..|::.||:.:|.|||||||.|
YME1L1 635 FGMSEKLGVMTYS-----DTG-KLSPETQSAIEQEIRILLRDSYERAKHI    678

YME1L  706 LRKHTREHKALAEALLKYETLDADDIKAILNESQT---    740
           |:.|.:|||.||||||.||||||.:|:.:|...:.
YME1L1 679 LKTHAKEHKNLAEALLTYETLDAKEIQIVLEGKKLEVR    716
```

Suppl. Mat. 2. Alignments of *Drosophila* proteins and their human orthologues. Protein sequences were sourced from https://www.ensembl.org with information provided in brackets listing the unique sequence identifiers (fly: FBtr, FlyBase transcript; human: ENST, Ensembl Transcript), the used isoforms and their mRNA (nt) and protein lengths (aa). 'DIOPT alignment' shows the key information of the alignment by the '*Drosophila* Integrative Ortholog Prediction Tool' with further details accessible via the link provided. 'EMBOSS alignment' shows the alignment of the selected and above-shown protein sequences manually carried out using https://www.ebi.ac.uk/jdispatcher/psa/emboss_needle; vertical

lines indicate identity, colons strong similarity (conservative substitution), and single dots weaker similarity (semi-conservative substitution).
